# Supplementary material for: Adapting the FAST-M maternal sepsis intervention for implementation in Pakistan: a qualitative exploratory study
Source: BMJ Open. 2022 Sep 9;12(9):e059273. doi: 10.1136/bmjopen-2021-059273 (PMC9472171; doi:10.1136/bmjopen-2021-059273)
Supplement: Supplementary data [file bmjopen-2021-059273supp004.pdf]

MODIFIED EARLY OBSTETRIC WARNING CHART (MEOWS CHART)

FAST-M

Contact clinical decision maker if patient triggers **ONE RED** or **TWO YELLOW** flags at any one time.

Patient | Patient ID | DOB/Age

Date

Time

Initials

WRITE **VALUES** IN BOXES PROVIDED

Parameter	Value	Flag
Respiratory rate (breaths per minute)	25 or more	RED
	21 - 24	YELLOW
	11 - 20	NORMAL
	10 or less	RED
Temperature (°C)	38 or more	YELLOW
	36.0 to 37.9	NORMAL
	35.9 or less	YELLOW
Heart rate (beats per minute)	120 or more	RED
	100 - 119	YELLOW
	50 - 99	NORMAL
	40 - 49	YELLOW
	39 or less	RED
Systolic blood pressure (mmHg)	160 or more	RED
	140 -159	YELLOW
	100 -139	NORMAL
	90 - 99	YELLOW
	89 or less	RED
Diastolic blood pressure (mmHg)	110 or more	RED
	90 -109	YELLOW
	40 - 89	NORMAL
	39 or less	RED
Urine output Hours since patient passed urine (tick box)	12 hours or less	NORMAL
	12 - 18 hours	YELLOW
	18 hours or more OR less than 0.5 ml/kg/hour	RED
Mental State (tick box)	Alert	NORMAL
	Not Alert	RED
Looks unwell (tick box)	No	NORMAL
	Yes	YELLOW

TOTAL YELLOW FLAGS

TOTAL RED FLAGS

ACTION TAKEN (IF REQUIRED) Yes (Y) / No (N)

ACT NOW if patient triggers ONE RED or TWO YELLOW flags at any time. Escalate to clinical decision maker and start FAST-M decision tool.

UNITED AGAINST MATERNAL SEPSIS

THINK SEPSIS, ACT FAST-M

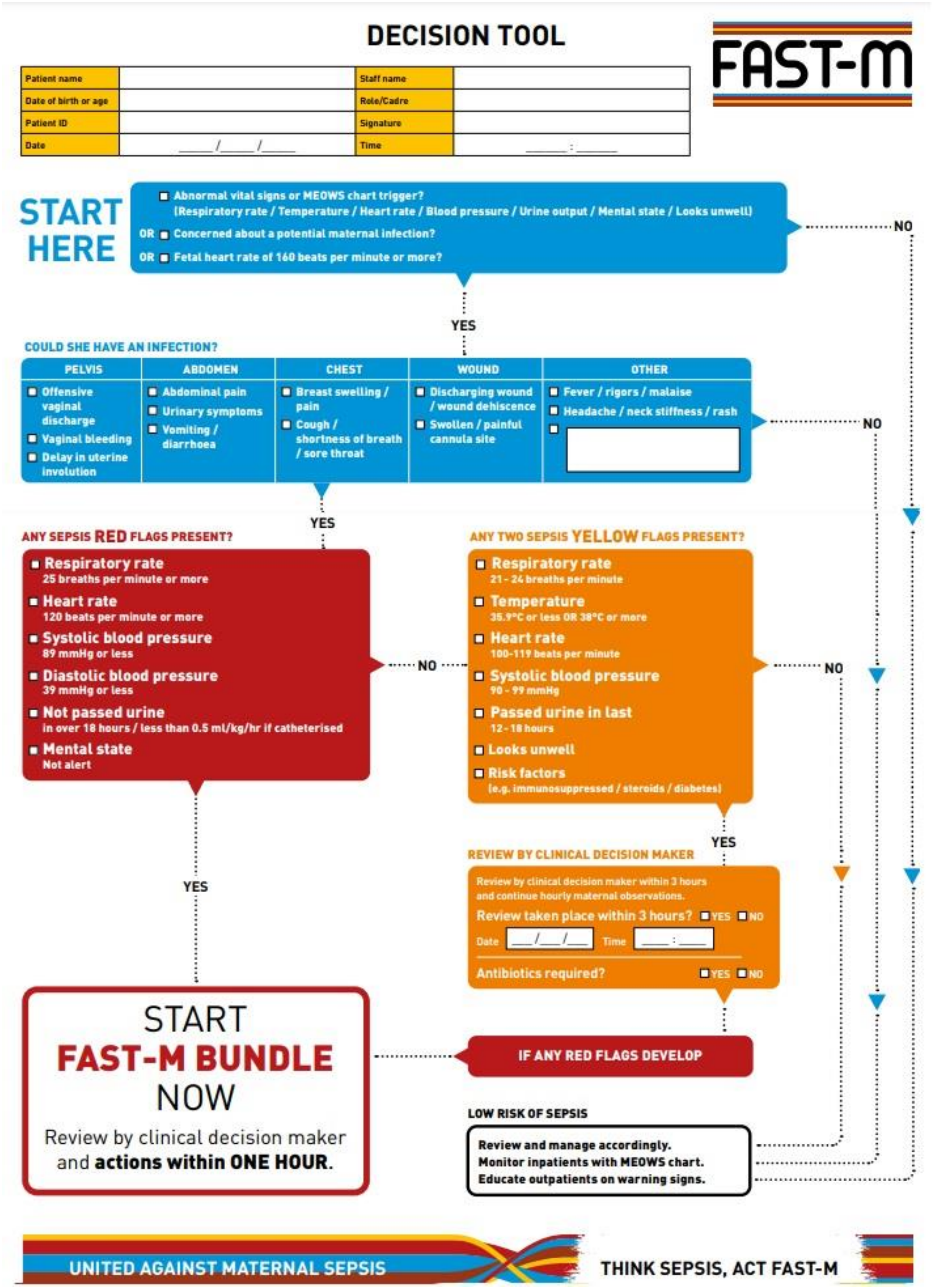
DECISION TOOL
FAST-M
Patient name
Date of birth or age
Patient ID
Date
___/___/___
Staff name
Role/Cadre
Signature
Time
___:___
START HERE
Abnormal vital signs or MEOWS chart trigger?
(Respiratory rate / Temperature / Heart rate / Blood pressure / Urine output / Mental state / Looks unwell)
OR Concerned about a potential maternal infection?
OR Fetal heart rate of 160 beats per minute or more?
NO
YES
COULD SHE HAVE AN INFECTION?
PELVIS
Offensive vaginal discharge
Vaginal bleeding
Delay in uterine involution
ABDOMEN
Abdominal pain
Urinary symptoms
Vomiting / diarrhoea
CHEST
Breast swelling / pain
Cough / shortness of breath / sore throat
WOUND
Discharging wound / wound dehiscence
Swollen / painful cannula site
OTHER
Fever / rigors / malaise
Headache / neck stiffness / rash
NO
YES
ANY SEPSIS RED FLAGS PRESENT?
Respiratory rate
25 breaths per minute or more
Heart rate
120 beats per minute or more
Systolic blood pressure
89 mmHg or less
Diastolic blood pressure
39 mmHg or less
Not passed urine
in over 18 hours / less than 0.5 ml/kg/hr if catheterised
Mental state
Not alert
NO
ANY TWO SEPSIS YELLOW FLAGS PRESENT?
Respiratory rate
21 - 24 breaths per minute
Temperature
35.9°C or less OR 38°C or more
Heart rate
100-119 beats per minute
Systolic blood pressure
90 - 99 mmHg
Passed urine in last
12-18 hours
Looks unwell
Risk factors
(e.g. immunosuppressed / steroids / diabetes)
NO
YES
REVIEW BY CLINICAL DECISION MAKER
Review by clinical decision maker within 3 hours and continue hourly maternal observations.
Review taken place within 3 hours? YES NO
Date ___/___/___ Time ___:___
Antibiotics required? YES NO
YES
START
FAST-M BUNDLE
NOW
Review by clinical decision maker and actions within ONE HOUR.
IF ANY RED FLAGS DEVELOP
LOW RISK OF SEPSIS
Review and manage accordingly.
Monitor inpatients with MEOWS chart.
Educate outpatients on warning signs.
UNITED AGAINST MATERNAL SEPSIS
THINK SEPSIS, ACT FAST-M

TREATMENT BUNDLE

FAST-M

**Patient name**			**Staff name**		
**D.O.B or age**			**Role/Cadre**		
**Patient ID**			**Signature**		
**Date & time of red flag observation**	__/__/__ __:__	**Date & time bundle started**	__/__/__ __:__	**Date & time of review by clinical decision maker**	__/__/__ __:__

REMEMBER TO COMPLETE THESE ACTIONS WITHIN ONE HOUR

F — FLUIDS (caution in pre-eclampsia, severe anaemia and pulmonary oedema)

Date	__/__/__	Time fluids initiated	__:__	Initials		**Give 500 ml crystalloid immediately. Repeat 500 ml boluses to a maximum of 30 ml/kg if hypotension persists.**
Details / reason not completed						

A — ANTIBIOTICS

Date	__/__/__	Time started	__:__	Initials		**See antibiotic guidelines below**
Details / reason not completed						

S — SOURCE – identify and treat the source of infection

Date	__/__/__	Time considered	__:__	Initials		**See source identification and treatment boxes below**
Details / reason not completed						

T — TRANSPORT (to higher level hospital or location within hospital, if required)

Date & time transport considered	__/__/__ __:__	Initials		Transport Required	☐ YES ☐ NO
Date & time transport requested	__/__/__ __:__	Initials		☐ N/A	
Date & time patient left facility	__/__/__ __:__	Initials			
Destination					
Reason for any delay					

M — MONITORING (start MEOWS chart if not already started. Repeat observations every 30 minutes until otherwise decided by clinical decision maker)

Date & time monitoring commenced	__/__/__ __:__		Details / reason not completed
Maternal / fetal monitoring should include	• Respiratory rate<br>• Temperature<br>• Heart rate<br>• Blood pressure	• Urine output<br>• Mental state<br>• Fetal heart rate	
Neonatal monitoring and review commenced	☐ YES ☐ NO ☐ N/A		

ANTIBIOTIC GUIDELINES

Insert local guidance here

Immediate treatment for Maternal Sepsis:

- Ceftriaxone 2 g IV once daily (if no IV access this can be given as 2 IM injections of 1 g in different sites).
- If possible intra-abdominal source add Metronidazole 500 mg IV three times daily or 400 mg PO three times daily.

If above antibiotic regime is not available then give:

- Chloramphenicol 1 g IV/IM four times daily plus Gentamycin 240 mg IV/IM once daily.

If maternal infection source is known, or as soon as it is identified:

- Use specific treatment based on Malawi Standard Treatment Guidelines.

IDENTIFY THE SOURCE

Consider

• Clinical history	• Blood cultures	• Sputum sample
• Clinical examination	• HIV and Malaria testing	• Imaging (abdominal, chest)
• Blood tests (if available) (FBC, U&Es, LFTs, CRP, clotting)	• Urine sample	• Lumbar puncture
	• Swabs (wound, vagina, throat)	• Other ________

REMOVE / TREAT THE SOURCE

Consider

• Malaria treatment	• Removal of infected cannula / line
• Consider delivery of baby	• Hysterectomy
• Removal of retained products of conception	• Targeted antibiotics once source known
• Debridement of wound / drainage of collection	

UNITED AGAINST MATERNAL SEPSIS — **THINK SEPSIS, ACT FAST-M**

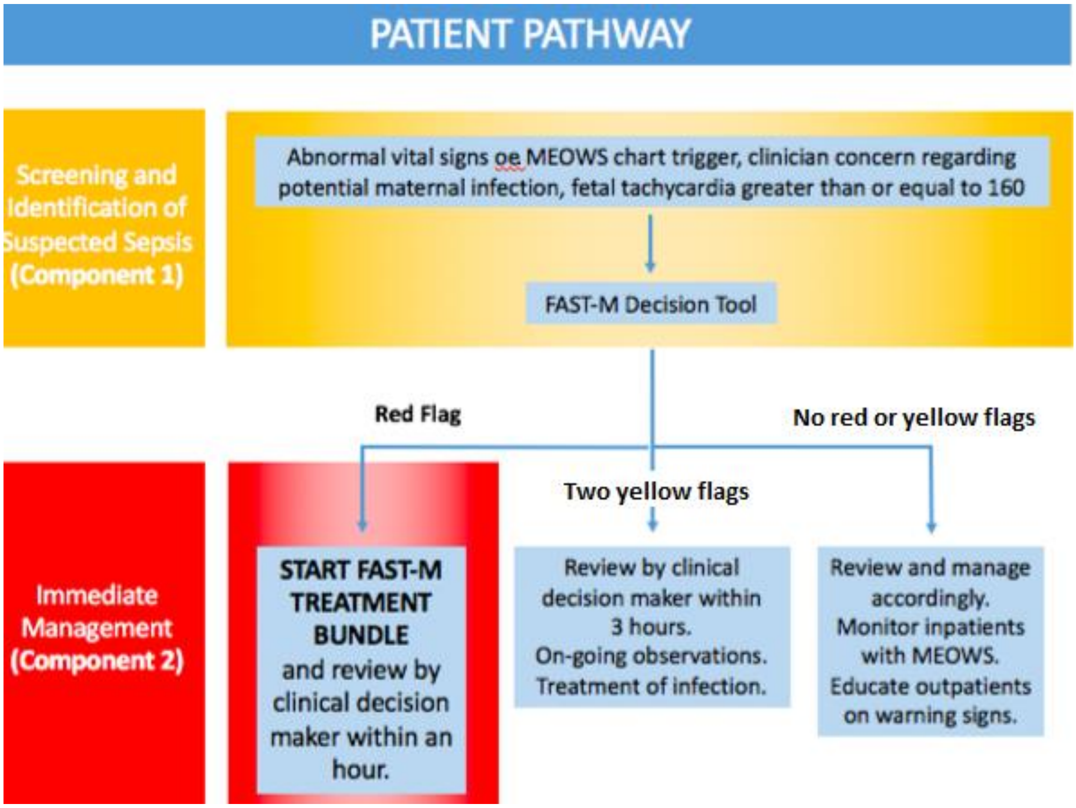
PATIENT PATHWAY
Screening and Identification of Suspected Sepsis (Component 1)
Abnormal vital signs oe MEOWS chart trigger, clinician concern regarding potential maternal infection, fetal tachycardia greater than or equal to 160
FAST-M Decision Tool
Red Flag
No red or yellow flags
Two yellow flags
Immediate Management (Component 2)
START FAST-M TREATMENT BUNDLE and review by clinical decision maker within an hour.
Review by clinical decision maker within 3 hours. On-going observations. Treatment of infection.
Review and manage accordingly. Monitor inpatients with MEOWS. Educate outpatients on warning signs.
