# Supplementary material for: Adapting the FAST-M maternal sepsis intervention for implementation in Pakistan: a qualitative exploratory study
Source: BMJ Open. 2022 Sep 9;12(9):e059273. doi: 10.1136/bmjopen-2021-059273 (PMC9472171; doi:10.1136/bmjopen-2021-059273)
Supplement: Supplementary data [file bmjopen-2021-059273supp005.pdf]

FORM 1: FACILITY AUDIT

FACILITY VIDEO ID: ☐☐☐ – ☐☐☐

Facility ID e.g. UWK, KAB etc. *Visit ID e.g. 001, 002 etc.*

Today's Date: d d – m m – y y y y Time: ☐☐ : ☐☐

Date previous form completed (or if first Visit, date study opened at this site): d d – m m – y y y y

Are you collecting the data during the baseline or intervention phase? **Baseline** ☐ Intervention ☐

3. How many of the following MATERNAL OUTCOMES have you had since the last visit?

Maternal Outcome	Number
Maternal Sepsis	
Maternal Deaths	
Post-Partum haemorrhage (>1L)	
Ante-Partum haemorrhage (>50ml)	
Severe pre-eclampsia/eclampsia (>160/110 and >2+ protein in urine)	
Blood transfusions	

4. How many of the following NEONATAL OUTCOMES have you had since the last visit?

Neonatal Outcome	Number
Live Births	
Neonatal deaths before discharge from hospital	
Babies requiring antibiotics	
Still births	

Ahmed SI, *et al. BMJ Open* 2022; 12:e059273. doi: 10.1136/bmjopen-2021-059273

FORM 1: FACILITY AUDIT

FACILITY VISIT ID: ☐☐☐ – ☐☐☐

Facility ID e.g. UWK, KAB etc.

Visit ID e.g. 001, 002 etc.

5. How many of the following RESOURCES are available today?

Resource	Availability		
	**Good ***	**Limited**	**None**
Types of IV fluid:			
A) 0.9% Saline	☐	☐	☐
B) Ringers Lactate	☐	☐	☐
Type of oral antibiotics:			
A) Amoxicillin	☐	☐	☐
B) Augmentin	☐	☐	☐
C) Cephalosporin (e.g. Cefalexin)	☐	☐	☐
D) Cefixime	☐	☐	☐
E) Ciprofloxacin	☐	☐	☐
F) Clindamycin	☐	☐	☐
G) Doxycycline	☐	☐	☐
H) Erythromycin	☐	☐	☐
I) Metronidazole	☐	☐	☐
J) Other (please state):	☐	☐	
Type of IM / IV antibiotics:			
A) Ampicillin	☐	☐	☐
B) Penicillin G	☐	☐	☐
C) Cefazolin	☐	☐	☐
D) Cephalosporin (e.g. ceftriazone etc.)	☐	☐	☐
E) Ciprofloxacin	☐	☐	☐
F) Clindamycin	☐	☐	☐

Ahmed SI, *et al. BMJ Open* 2022; 12:e059273. doi: 10.1136/bmjopen-2021-059273

FORM 1: FACILITY AUDIT

FACILITY VISIT ID: ☐☐☐ – ☐☐☐

Facility ID e.g. UWK, KAB etc. *Visit ID e.g. 001, 002 etc.*

G) Gentamycin	☐	☐	☐
H) Metronidazole	☐	☐	☐
I) Vancomycin	☐	☐	☐
J) Other (please state):	☐	☐	

* Good availability defined as a supply that is unlikely to run out before the next anticipated delivery

**Resource**	**Availability**		
	**Good ***	**Limited**	**None**
Equipment for IV line (cannula, dressings etc)	☐	☐	☐
Malaria tests	☐	☐	☐
Functioning theatre and staff able to remove source of infection	☐	☐	☐
Adequate means to transport to transfer patients for specialist care	☐	☐	☐
Working thermometers	☐	☐	☐
Working BP machines	☐	☐	☐
Working O2 saturation machines	☐	☐	☐
Fetoscopes / Pinnards / fetal stethoscopes	☐	☐	☐
Clocks / watches	☐	☐	☐
Spare batteries	☐	☐	☐
FAST-M Observation charts *1	☐	☐	☐
FAST-M Decision tools *1	☐	☐	☐
FAST-M Treatment tools *1	☐	☐	☐
FAST-M Referral letter *1	☐	☐	☐

* Good availability defined as a supply that is unlikely to run out before the next anticipated delivery

*[1] only applicable if completing form during the intervention phase

Ahmed SI, *et al. BMJ Open* 2022; 12:e059273. doi: 10.1136/bmjopen-2021-059273

BMJ Publishing Group Limited (BMJ) disclaims all liability and responsibility arising from any reliance placed on this supplemental material which has been supplied by the author(s)

FORM 1: FACILITY AUDIT

FACILITY VISIT ID: ☐☐☐ – ☐☐☐

Facility ID e.g. UWK, KAB etc.

Visit ID e.g. 001, 002 etc.

Completed by: ______________________

Role: ______________________

Signature: ______________________

Date: *DD* / *MMM* / *YYYY*

You must have signed the Site Signature & Delegation Log

Ahmed SI, *et al. BMJ Open* 2022; 12:e059273. doi: 10.1136/bmjopen-2021-059273
