# Supplementary material for: Adapting the FAST-M maternal sepsis intervention for implementation in Pakistan: a qualitative exploratory study
Source: BMJ Open. 2022 Sep 9;12(9):e059273. doi: 10.1136/bmjopen-2021-059273 (PMC9472171; doi:10.1136/bmjopen-2021-059273)
Supplement: Supplementary data [file bmjopen-2021-059273supp006.pdf]

MODIFIED EARLY OBSTETRIC WARNING CHART (MEOWS CHART)

FAST-M

Contact clinical decision maker if patient triggers **ONE RED** or **TWO YELLOW** flags at any one time.

Patient		Patient ID		DOB/Age	

Date															
Time															
Initials															

WRITE **VALUES** IN BOXES PROVIDED

Respiratory rate (breaths per minute)	25 or more	RED															
	21 - 24	YELLOW															
	11 - 20	NORMAL															
	10 or less	RED															
Oxygen saturations (%)	95 or more	NORMAL															
	94 or less OR needing oxygen	RED															
Temperature (°C)	38 or more	YELLOW															
	36.0 to 37.9	NORMAL															
	35.9 or less	YELLOW															
Heart rate (beats per minute)	120 or more	RED															
	100 - 119	YELLOW															
	50 - 99	NORMAL															
	40 - 49	YELLOW															
	39 or less	RED															
Systolic blood pressure (mmHg)	160 or more	RED															
	140 - 159	YELLOW															
	100 - 139	NORMAL															
	90 - 99	YELLOW															
	89 or less	RED															
Diastolic blood pressure (mmHg)	110 or more	RED															
	90 - 109	YELLOW															
	40 - 89	NORMAL															
	39 or less	RED															
Urine output (hours since patient passed urine) (tick box)	12 hours or less	NORMAL															
	12 - 18 hours	YELLOW															
	18 hours or more OR less than 0.5 mL/kg/hour	RED															
Mental State (tick box)	Alert	NORMAL															
	Not Alert	RED															
Looks unwell (tick box)	No	NORMAL															
	Yes	YELLOW															
TOTAL YELLOW FLAGS																	
TOTAL RED FLAGS																	
ACTION TAKEN (IF REQUIRED) Yes (Y) / No (N)																	

DECISION TOOL

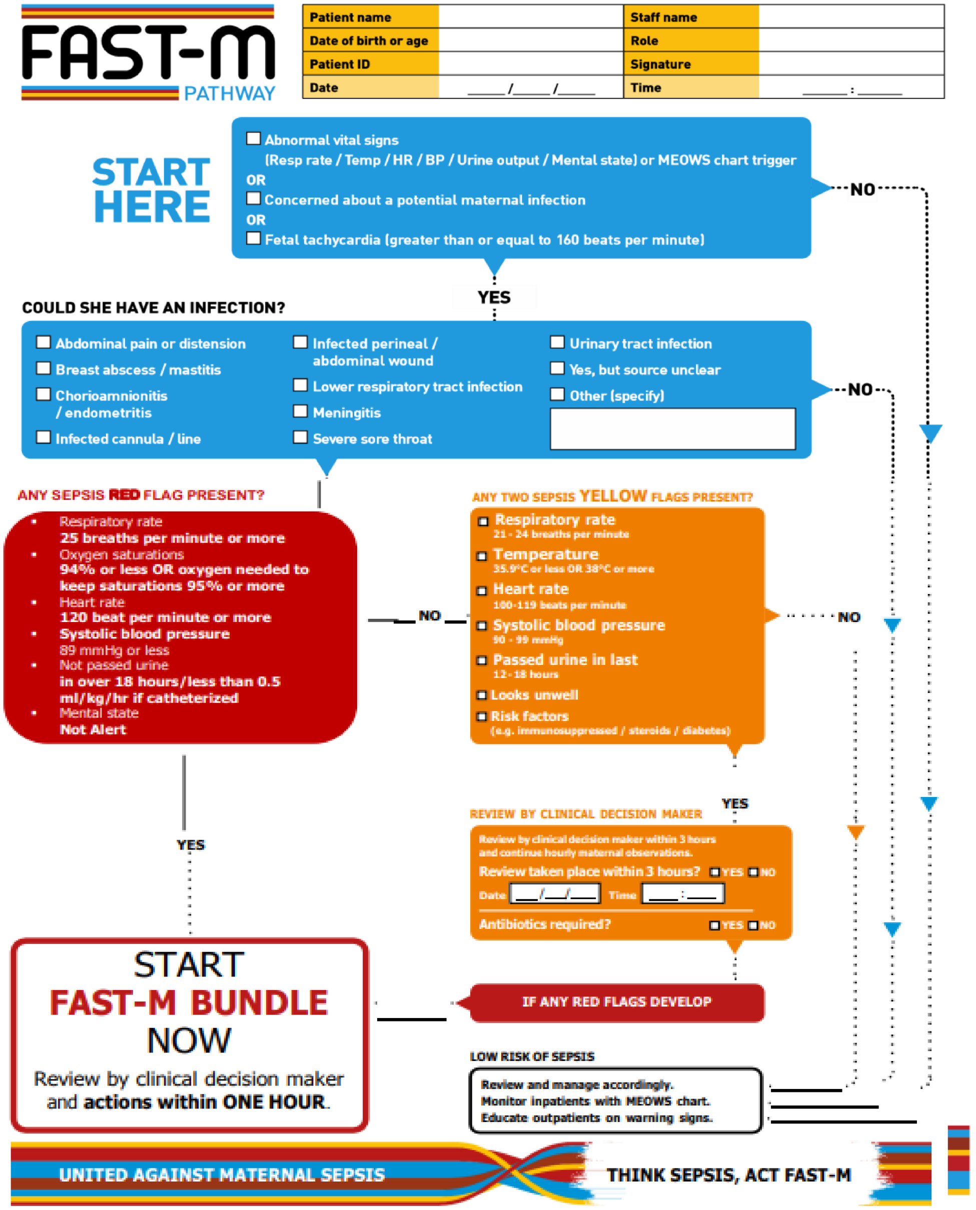

TREATMENT BUNDLE

FAST-M

Patient name		Staff name	
D.O.B or age		Role/Cadre	
Patient ID		Signature	

Date & time of red flag observation	—/— /— —:—	Date & time bundle started	—/—/— —:—	Date & time of review by clinical decision maker	—/— /— —:—

REMEMBER TO COMPLETE THESE ACTIONS WITHIN ONE HOUR

F — FLUIDS (caution in pre-eclampsia, severe anaemia and pulmonary oedema)

Date	__/__/__	Time fluids initiated	__:__	Initials		Give 500 ml crystalloid immediately. Repeat 500 ml boluses to a maximum of 30 ml/kg if hypotension persists.
Details / reason not completed						

A — ANTIBIOTICS

Date	__/__/__	Time started	__:__	Initials		See antibiotic guidelines below
Details / reason not completed						

S — SOURCE – identify and treat the source of infection

Date	__/__/__	Time considered	__:__	Initials		See source identification and treatment boxes below
Details / reason not completed						

T — TRANSPORT (to higher level hospital or location within hospital, if required)

Date & time transport considered	__/__/__ __:__	Initials		Transport Required	☐ YES ☐ NO
Date & time transport requested	__/__/__ __:__	Initials		☐ N/A	
Date & time patient left facility	__/__/__ __:__	Initials			
Destination					
Reason for any delay					

M — MONITORING (start MEOWS chart if not already started. Repeat observations every 30 minutes until otherwise decided by clinical decision maker)

Date & time monitoring commenced	—/__/__ __:__	Details / reason not completed
Maternal / fetal monitoring should include	• Respiratory rate<br>• Oxygen Saturations<br>• Temperature<br>• Heart rate<br>• Blood pressure<br>• Urine output<br>• Mental state<br>• Fetal heart rate	
Neonatal monitoring and review commenced	☐ YES ☐ NO ☐ N/A	

ANTIBIOTIC GUIDELINES

Insert local guidance here

Immediate treatment for Maternal Sepsis:

- Ceftriaxone 2 g IV once daily (if no IV access this can be given as 2 IM injections of 1 g in different sites).
- If possible intra-abdominal source add Metronidazole 500 mg IV three times daily or 400 mg PO three times daily.

If above antibiotic regime is not available then give:

- Tazobactam 4.5 g IV daily two time a day
- Meropenem 1 g IV daily two times a day

IDENTIFY THE SOURCE

Consider

- Clinical history
- Clinical examination
- Blood tests (if available) (FBC, U&Es, LFTs, CRP, clotting)
- Blood cultures
- HIV and Malaria testing
- Urine sample
- Swabs (wound, vagina, throat)
- Sputum sample
- Imaging (abdominal, chest)
- Lumbar puncture
- Other ____________

REMOVE / TREAT THE SOURCE

Consider

- Malaria treatment
- Consider delivery of baby
- Removal of retained products of conception
- Debridement of wound / drainage of collection
- Removal of infected cannula / line
- Hysterectomy
- Targeted antibiotics once source known

UNITED AGAINST MATERNAL SEPSIS

THINK SEPSIS, ACT FAST-M
