# Supplementary material for: Adapting the FAST-M maternal sepsis intervention for implementation in Pakistan: a qualitative exploratory study
Source: BMJ Open. 2022 Sep 9;12(9):e059273. doi: 10.1136/bmjopen-2021-059273 (PMC9472171; doi:10.1136/bmjopen-2021-059273)
Supplement: Author's manuscript [file bmjopen-2021-059273.draft_revisions.pdf]

BMJ Open is committed to open peer review. As part of this commitment we make the peer review history of every article we publish publicly available.

When an article is published we post the peer reviewers' comments and the authors' responses online. We also post the versions of the paper that were used during peer review. These are the versions that the peer review comments apply to.

The versions of the paper that follow are the versions that were submitted during the peer review process. They are not the versions of record or the final published versions. They should not be cited or distributed as the published version of this manuscript.

BMJ Open is an open access journal and the full, final, typeset and author-corrected version of record of the manuscript is available on our site with no access controls, subscription charges or pay-per-view fees (http://bmjopen.bmj.com).

If you have any questions on BMJ Open's open peer review process please email info.bmjopen@bmj.com

BMJ Open

Adapting the FAST-M maternal sepsis intervention for implementation in Pakistan: A qualitative exploratory study

Journal:	*BMJ Open*
Manuscript ID	bmjopen-2021-059273
Article Type:	Original research
Date Submitted by the Author:	16-Nov-2021
Complete List of Authors:	Ahmed, Sheikh; The Aga Khan University, Obstetrics and Gynecology<br>Khowaja, Bakhtawar; The Aga Khan University, Obstetrics and Gynecology<br>Barolia, Rubina; Aga Khan University,<br>Sikandar , Raheel ; Liaquat University of Medical and Health Sciences<br>Rind , Kubra ; The Aga Khan University, Obstetrics and Gynecology<br>Khan , Sehrish ; Liaquat University of Medical and Health Sciences<br>Rani , Raheela ; Liaquat University of Medical and Health Sciences<br>Cheshire , James ; University of Birmingham<br>Dunlop, Catherine; Birmingham Women's Hospital, Academic, 3rd Floor<br>Coomarasamy, Arri; University of Birmingham,<br>Sheikh, Lumaan; The Aga Khan University Hospital, Obstetric &Gynecology<br>Lissauer, David ; University of Liverpool
Keywords:	INFECTIOUS DISEASES, OBSTETRICS, Health & safety < HEALTH SERVICES ADMINISTRATION & MANAGEMENT, Public health < INFECTIOUS DISEASES

SCHOLARONE™
Manuscripts

I, the Submitting Author has the right to grant and does grant on behalf of all authors of the Work (as defined in the below author licence), an exclusive licence and/or a non-exclusive licence for contributions from authors who are: i) UK Crown employees; ii) where BMJ has agreed a CC-BY licence shall apply, and/or iii) in accordance with the terms applicable for US Federal Government officers or employees acting as part of their official duties; on a worldwide, perpetual, irrevocable, royalty-free basis to BMJ Publishing Group Ltd ("BMJ") its licensees and where the relevant Journal is co-owned by BMJ to the co-owners of the Journal, to publish the Work in this journal and any other BMJ products and to exploit all rights, as set out in our licence.

The Submitting Author accepts and understands that any supply made under these terms is made by BMJ to the Submitting Author unless you are acting as an employee on behalf of your employer or a postgraduate student of an affiliated institution which is paying any applicable article publishing charge ("APC") for Open Access articles. Where the Submitting Author wishes to make the Work available on an Open Access basis (and intends to pay the relevant APC), the terms of reuse of such Open Access shall be governed by a Creative Commons licence – details of these licences and which Creative Commons licence will apply to this Work are set out in our licence referred to above.

Other than as permitted in any relevant BMJ Author's Self Archiving Policies, I confirm this Work has not been accepted for publication elsewhere, is not being considered for publication elsewhere and does not duplicate material already published. I confirm all authors consent to publication of this Work and authorise the granting of this licence.

For peer review only

Paper Title:

Adapting the FAST-M maternal sepsis intervention for implementation in Pakistan: A qualitative exploratory study

Author Names:

Sheikh Irfan Ahmed[1]

Bakhtawar M.Hanif Khowaja[1]

Rubina Barolia[1]

Raheel Sikandar[2]

Kubra Rind[1]

Sehrish Khan[2]

Raheela Rani[2]

James Cheshire[3]

Catherine Dunlop [3]

Arri Coomarasamy[3]

Lumaan Sheikh[1]

David Lissauer[4, 5]

Affiliations & full institutional mailing addresses of all authors

[1] Aga Khan University Hospital, National Stadium Road, Karachi city, Pakistan. P.O. Box 3500 Postal code: 74800

[2] LUMHS Hospital Liaquat University of Health and Medical Sciences, Hyderabad city, Pakistan. Postal Code: 76090

[3] Institute of Metabolism and Systems Research, University of Birmingham, Edgbaston, Birmingham, UK, B15 2TT.

For peer review only

[4] Institute of Life Course and Medical Sciences, University of Liverpool, Liverpool, William Henry Duncan Building, Liverpool, UK, L7 8TX.

[5] Malawi-Liverpool-Wellcome Trust Clinical Research Programme, Chichiri, Blantyre 3, Malawi

Email addresses

sheikh.irfan@aku.edu

bakhtawar.hanif@aku.edu

rubina.barolia@aku.edu

raheel.sikandar@lumhs.edu.pk

ghulam.kubra@aku.edu

drkhanhyd@gmail.com

raheela_rani@hotmail.com

james.cheshire@nhs.net

catherinedunlop@nhs.net

A.Coomarasamy@bham.ac.uk

lumaan.sheikh@aku.edu

David.Lissauer@liverpool.ac.uk

Corresponding Author*:

Sheikh Irfan Ahmed

For peer review only

For peer review only - http://bmjopen.bmj.com/site/about/guidelines.xhtml

Abstract

Objective

A maternal sepsis management bundle for resource limited settings has been developed through a synthesis of evidence and international consensus. This bundle, called "FAST-M" consists of five components: **F**luids, **A**ntibiotics, **S**ource control, assessment of the need to **T**ransport/Transfer to a higher level of care and ongoing **M**onitoring (of the mother and neonate). This study aimed to adapt the FAST-M bundle in the context of Pakistan and to identify the potential facilitators and barriers to its implementation in a low resource setting within Pakistan.

Setting

The study was conducted at the Liaquat University of Medical and Health Sciences, Hyderabad.

Design and Participants

A qualitative exploratory study comprising of key-informant interviews and a focus group discussion was conducted with healthcare providers (doctors, nurses and healthcare administrators) working at the study setting.

Results

Four overarching themes were identified, the hindering factors for implementation of the FAST-M intervention were: **(I) Challenges in existing systems** such as a shortage of resources and lack of quality assurance; and **(II) Clinical practice variation** that includes lack of sepsis guidelines and documentation; the facilitating factors identified were: **(III) Health care providers' perceptions about the FAST-M intervention** and their positive views about its

For peer review only

For peer review only - http://bmjopen.bmj.com/site/about/guidelines.xhtml

execution; and **(IV) Development of HCPs readiness for FAST-M implementation** that aided in identifying solutions to potential hindering factors at their clinical setting.

Conclusion

The study has identified potential gaps and their probable solutions prior to implementation of FAST-M intervention. The study also identified facilitators for FAST-M implementation that may help in effective uptake of FAST-M intervention.

Keywords: FAST-M intervention, maternal sepsis, Pakistan, qualitative study, sepsis bundle, care bundle, complex intervention, low-resource setting, feasibility study

Strengths and Limitations of this study

- The major strength of this study is the use of CFIR, which we used to gather data through development of interview guides using CFIR domains.
- We collected data from multiple levels of HCPs using different methods of data collection i.e. individual interviews and focus group discussion to triangulate our findings and establish trustworthiness of the study.
- The key informant interviews focused mainly on the doctor's perspective due to the prominent role of doctors at the study setting which limited us to gain perceptions of other healthcare providers.

For peer review only - http://bmjopen.bmj.com/site/about/guidelines.xhtml

For peer review only

Background

Maternal sepsis is a major contributor to maternal morbidity and mortality worldwide [1]. Maternal sepsis is a life-threatening organ dysfunction caused by a dysregulated host response due to infection during pregnancy, childbirth and in postpartum period [2, 3].

Globally, maternal sepsis accounts for about one tenth of maternal deaths and is the third most common cause of maternal mortality [1, 4]. It was estimated that each year 75,000 maternal deaths occurred in low and middle income countries due to maternal sepsis and approximately 10% of maternal deaths in Africa and Asia occurs due to sepsis [5,6]. The risk of death among women who develop puerperal sepsis was higher in Africa (odds ratio 2.71), Asia (1.91), and Latin America and the Caribbean (2.06) than in developed countries. [6].

Led by the World Health Organization and other partners, a global initiative was commenced in 2015, to develop strategies aimed at improving early recognition and management of maternal sepsis [7]. Strategies to ensure early identification and treatment of sepsis have demonstrated significant improvement in outcomes in high income adult population settings [8] and it was necessary to translate these approaches into the maternity population and make them appropriate for low resource settings [8]. Yet, there is very limited evidence of implementation of such approaches specific to maternity care in low-resource settings.

Thus, a maternal sepsis bundle was developed as part of this process to improve the recognition and management of maternal sepsis in a low-resource setting. A modified Delphi approach was adopted to identify components significant to treatment and monitoring in terms of clinical importance and feasibility in resource-poor settings [9]. The components selected were: **F**luids, **A**ntibiotics, **S**ource control, assessment of the need to **T**ransport/Transfer to a higher level of

For peer review only - http://bmjopen.bmj.com/site/about/guidelines.xhtml

care and ongoing **M**onitoring (of the mother and neonate). The bundle was named “FAST-M” as a memorable acronym for both communication and awareness-raising [9].

Implementation of the FAST-M intervention across 15 government healthcare facilities in Malawi was found to not only be feasible but also resulted in improved clinical care [10], demonstrating that the intervention could assist in the early identification and management of maternal sepsis in low-resource settings [10]. This is now being tested formally as part of a large cluster-randomised trial across Malawi and Uganda.

In Pakistan, complications during pregnancy and childbirth are the leading causes of death in women, accounting for 20% of all deaths of women of child-bearing age [11-13]. National figures show that 15% of maternal deaths are reported due to sepsis [13] and maternal sepsis is established as the 3rd leading cause of maternal mortality [14]. Globally, the incidence of puerperal sepsis is 4.4% [14] whereas in Pakistan the incidence is reported to be 10-15% [15].

There are national sepsis guidelines for Pakistan (SGP) which are designed to aid in the identification and management of sepsis in adults in the local settings and are modeled on the Surviving Sepsis Campaign (SSC) [16]. However, these are inconsistently applied and lack a comprehensive implementation approach. There is still uncertainty about how best to optimise the implementation of evidence based practices around maternal sepsis prevention and management in Pakistan.

It is therefore planned to adapt and implement the FAST-M intervention in Pakistan. However, we recognise that to optimise its use in Pakistani context requires a robust process of adaptation and re-design prior to its field testing. The implementation of FAST-M intervention will be highly context specific and therefore, this study aims to understand the existing sepsis

For peer review only - http://bmjopen.bmj.com/site/about/guidelines.xhtml

management practices and behaviours to adapt the FAST-M bundle care tools in local context. In addition, it will assist in identification of the potential facilitators and barriers to its implementation in a low resource setting within Pakistan.

This qualitative study was conducted in preparation for the implementation of FAST-M intervention in phase II of the study. The study findings obtained in this formative research will aid in the development of feasible methods to improve the processes and implementation of FAST-M intervention in Pakistan.

Methods

Study Design

Our methods, grounded in implementation science, aimed to identify the anticipated facilitators and barriers in implementation of FAST-M intervention at the Liaquat University of Medical Health Sciences (LUMHS), Hyderabad. Implementation research aims to identify the factors that function as barriers and enablers to specific interventions [17]. As our research question is descriptive and exploratory, this formative research adopted a qualitative research design involving both focus group discussion (FGD) and key-informant interviews and a purposive sampling approach.

Focus group discussion (FGD) and key-informant interviews (KIIs) were conducted with health care providers working at the study site using interview guides structured using the CFIR framework [17]. The aim of FGD and KIIs was to engage health practitioners, government officials and other key stakeholders to understand the behavior of existing practices at the study setting for maternal sepsis care, identify various facilitators and barriers that may influence the implementation of FAST-M intervention and inform the adaptation of FAST-M tools and

For peer review only - http://bmjopen.bmj.com/site/about/guidelines.xhtml

implementation approach according to the local context. Data collection through key informant interviews and FGD were to ensure data triangulation through different methods ensuring credibility of the study findings. The present study is being stated as per the guidance provided in consolidated criteria for reporting qualitative research (see online supplemental file 1).

Consolidated Framework for Implementation Research

The CFIR is a 'meta-theoretical' framework that provides an overarching analysis for implementation [17]. It offers an extensive and standardized list of constructs that allow researchers to identify various variables that are most relevant to a particular intervention [18]. The CFIR consists of five major domains: intervention characteristics, outer setting, inner setting, characteristics of the individuals and the process of implementation. These domains are organized into 39 constructs (Table 1).

For peer review only

For peer review only - http://bmjopen.bmj.com/site/about/guidelines.xhtml

Table 1: CFIR domains and associated constructs

Domains	Constructs
**One: Intervention Characteristic**	Intervention Source<br>Evidence Strength and quality<br>Relative Advantage<br>Adaptability<br>Trialability<br>Complexity<br>Design Quality and packaging<br>Cost
**Two: Outer Setting**	Patient Needs and Resources<br>Cosmopolitanism<br>Peer Pressure<br>External Policies and Incentives
**Three: Inner Setting**	Structural characteristics<br>Networks & Communication<br>Culture<br>Implementation Climate<br>Tension for change<br>Compatibility<br>Relative priority<br>Organizational incentives and rewards<br>Goals and feedback<br>Learning climate<br>Readiness for implementation<br>Leadership engagement<br>Available resources<br>Access to knowledge and information
**Four: Characteristics of Individuals**	Knowledge and Beliefs about the intervention<br>Self-efficacy<br>Individual stage of change<br>Individual identification with organization<br>Other personal Attributes
**Five: Process**	Planning<br>Engaging<br>Opinion leaders<br>Formally appointed internal implementation leaders<br>Champions<br>External change agents<br>Executing<br>Reflecting and evaluating

For peer review only - http://bmjopen.bmj.com/site/about/guidelines.xhtml

CFIR has been used in various studies to inform qualitative processes across a range of complex intervention, because this flexible framework can be tailored to different settings across multiple contexts [18,19]. We therefore used the tailored CFIR framework to understand critical barriers and facilitators to implementation of FAST-M intervention that need to be addressed at multiple levels if the FAST-M intervention is to be successfully optimised, and adopted in healthcare practices in Pakistan.

Study setting

Liaquat University of Medical Health Sciences (LUMHS) is located in Hyderabad district, Pakistan. LUHMS is 1300 bed tertiary referral public sector hospital which serves a large number of mostly underprivileged populations. The hospital offers various facilities to both in-patient and out-patient. The hospital has three Obstetrics and Gynecology units and provides 24 hours emergency cover to patients coming from urban and rural areas of Sindh. It manages a high volume of cases with maternal sepsis every month. The current data from the facility shows that a total of approximately 11205 patients were admitted in OBGYN units from the period of January to August 2021; and the maternal mortality rate was recorded as 159/11205 (1.4%). Out of these 159 deaths, 45 were due to confirmed maternal sepsis (28.3%). These indicators direct that there is a need of a robust system to early detect and manage maternal sepsis cases in the hospital.

Patient and public involvement

There was no patient or public involvement in setting the research agenda.

Data collection methods and study participants

For peer review only

For peer review only - http://bmjopen.bmj.com/site/about/guidelines.xhtml

Healthcare providers working at LUMHS hospital were purposively sampled for KIIs and FGD. All types of healthcare providers including Doctors (residents and faculty members), staff nurses and administrators were represented. KIIs with healthcare providers were conducted in the meeting room and faculty offices at LUMHS hospital. A FGD was conducted in the seminar room at LUMHS hospital. A trained moderator facilitated the focus group discussion. The letters of invitation were sent to KIs and FGD participants for the qualitative study prior to interviews. Interviews were scheduled according to participants' preference, and were audio recorded following consent from study participants (Supplemental file 2).

Data collection procedure

A semi-structured interview guide was developed to explore healthcare professionals' views and attitudes towards the FAST-M intervention (Supplemental file 3), with a focus on the views on feasibility and adaptation of FAST-M implementation among healthcare professionals using five major domains of CFIR: intervention characteristics, outer setting, and inner setting, characteristics of the individuals and the process of implementation. Before beginning the interview, the qualitative researchers first described the FAST-M bundle components and the patient referral pathway (supplemental file 4) demonstrating the utilization of FAST-M bundle care tools. The interview guide underwent subsequent modifications and iterations based on interviews conducted.

A free flow of information was encouraged, using probes from these discussions to obtain healthcare professionals' perceptions about the adaptation and feasibility of FAST-M intervention. Interviews were conducted face-to-face in Urdu and English (KIIs = 16; FGD =1). The standards of precautions for control of COVID-19 infection were followed during data collection. All study participants were screened before interviews for COVID-19 infection

For peer review only - http://bmjopen.bmj.com/site/about/guidelines.xhtml

through a series of questions regarding their symptoms. The participants were asked to wear masks at all times during interviews and discussions. The focus group discussion was conducted in a large seminar room to maintain physical distance between participants as a precaution for control of COVID-19 infection.

Interviews and focus group discussion were conducted by RB, SI, BK, and GK, who are part of the investigating team and are trained in qualitative research. The research questions were based on FAST-M intervention characteristics, outer and inner health care setting, and characteristics of the individuals and the process of implementation. Detailed field notes were taken during each interview to capture non-verbal language and cues. KIIs were conducted for 20 minutes to 40 minutes; FGD was conducted for 50 minutes and consisted of 12 participants in a group. Data were collected using interview guides developed on five major domains of CFIR: intervention characteristics, outer setting, inner setting, characteristics of the individuals and the process of implementation. Data were collected and analyzed through an iterative process and data collection was ceased once saturation was achieved.

Data Analysis

Study data were analyzed using conventional qualitative content analysis approach facilitated by NVivo version 10 (QSR International, Pty Ltd) software. First, all the audio recordings were translated and transcribed from the local language (Urdu) into English. Transcripts were read several times to develop an interpretation of the participants' views about feasibility of FAST-M implementation. Focus group and KIIs were coded as one data set. Two investigators coded a subset of transcripts independently using separate coding that were then combined to match codes, and agreement by investigators was sought on a coding framework. Codes were formulated inductively from the transcripts related to research questions and CFIR domains.

For peer review only - http://bmjopen.bmj.com/site/about/guidelines.xhtml

Coding discrepancies were discussed and resolved to reduce researchers' biases. Codes were then analyzed into categories and then the major themes based on the data findings.

The potential barriers and facilitators were identified using the domains of CFIR and the final overarching themes were discussed and reviewed by the research team. To ensure credibility of the research, study data were triangulated by different data sources including doctors, nurses and administrators and through different data collection methods including FGD and KIIs, to compare alternative perspectives and to assess any inconsistencies.

Results

In this qualitative study, one FGD and sixteen KIIs (Table 2) were conducted with HCPs (doctors, nurses and health administrators), between November 2020 and January 2021, to ascertain the potential facilitators and barriers those can influence the implementation of FAST-M intervention at the study site. All the study participants (n = 28) who were approached by the study team agreed to participate in the study.

Table 2: Study participants

Focus group discussion with HCPs	Total FGD=1; n=12
**Doctors (Medicine); (OBGYN)**	n=3; n=5
**Nurses (OBGYN); (labor room)**	n=1; n=1
**Health administrators**	n=2
Key informant interviews	**Total KIIs= 16; n=16**
**Doctors (OBGYN); (Operating room); ICU**	n=8 ; n=1; n=2
**Nurses (OBGYN)**	n= 4

For peer review only - http://bmjopen.bmj.com/site/about/guidelines.xhtml

**Health administrators**	n= 1

Data analysis revealed four overarching themes: (I) Challenges in existing system; (II) Clinical practice variation; (III) Health care providers' perceptions about FAST-M; and (IV) Development of HCPs readiness for FAST-M implementation. Table 3 demonstrates the identified themes and categories.

Table 3: Themes and Categories

Themes	Categories
**Challenges in existing system**	Shortage of HCPs in the hospital
	Lack of adequate resources and quality assurance
**Clinical practice variation**	Sepsis guidelines and documentation
	Individual care practices and HCP comfort levels
**Health care providers' perceptions about FAST-M**	Understanding of the FAST-M bundle
	Perceptions about significance of FAST-M
	Identifying solutions to the application of FAST-M
**Development of HCPs readiness for FAST-M implementation**	Understanding and identifying gaps
	Consensus building for FAST-M implementation

For peer review only

Challenges in existing system

a. Shortage of HCPs in the hospital

A majority of the study participants reported challenges in the existing sepsis management practices. The major challenge reported by HCPs is the increased volume of patients coming to the obstetrics and gynecology inpatient wards and emergency room. The increased number of patients exaggerates workload on health care providers. The issue of a high patient to doctors' ratio that is 6:1; and high patient to nurses' ratio that is 20:1 was raised by a majority of study participants. There is a shortage of health workforce considering the influx of patients in the unit which is a hindering factor for provision of quality healthcare services.

> *"Being a tertiary level hospital, being a civil hospital and the main hospital, we are facing an increase patients flow on daily basis" (KII- Senior Registrar- OBGYN)*

> *"On floor, we have 6 doctors and you think how many patients are there. Sometimes we have 36 admissions; sometimes we have around 40 admissions. So, you can see for doctors to patients ratio it is around 6:1 and for staff, they are sometimes present and sometimes not" (KII- Senior Registrar)*

Health care providers identified that there is a considerable shortage of nurses in the hospital for the care of patients. The importance of nurse's role was acknowledged by all the key informants and focus group participants, and they emphasized the shortage of nurses for sepsis management in the unit as a key challenge, with only one or two nurses assigned to 20 patients in each shift.

For peer review only - http://bmjopen.bmj.com/site/about/guidelines.xhtml

As it was stated:

> *"Yes we are short of staff nurses. Look, if we have around 32 to 40 patients so there is only one nurse for their care or hardly two" (KII- Staff Nurse)*

> *"In emergency room, we do not have staff nurses available, so the doctor is responsible for maintaining IV line and catheterization. If there will be staff nurses available in the ER so they can help us with IV line, sending lab investigations and with catheterization. But this is a bitter truth that we have shortage of staff. No doubt the staff present in wards does work like they do patient's monitoring, IV medications and follow doctor's instructions" (KII- Admin Registrar)*

b. Lack of adequate resources and quality assurance

Health care providers, mainly doctors and nurses working in the unit, voiced concerns over scarcity of resources. All HCPs indicated their workplace as a low-resource setting and described private hospitals as having "*more resources than us*". Despite disparity in resources, HCPs generally believed they were maximizing sepsis management within the limits of what was possible in their unit.

> *"…this is not a private hospital and unit like that. This is civil hospital and we have to face many things. Our surroundings are not that favorable like it seems. We have to struggle a lot and this is the cause of delay of things. But anyways, we are trying our best to manage sepsis cases within our available resources" (KII- Registrar Admin)*

A majority of the patients present with complications and require intensive monitoring. There are High Dependency Units (HDUs) and Intensive Care Units (ICUs) in the hospital for critical

For peer review only - http://bmjopen.bmj.com/site/about/guidelines.xhtml

monitoring of the patients though the shortage of spaces in HDU and ICU is a challenge, as reported by the study participants.

"We have monitors available but not according to the patients need. We cannot monitor all the patients and we do it according to the severity of patient's condition. We have only two HDU beds and this is a challenge for us" (KII- Senior Registrar)

"We have 12 surgical and 12 medicine beds in ICUs altogether in LUMHS for all units. We face constraints of getting ICU beds for critical patients" (FGD- HOD)

The obstetrics and gynecology units has its own set of routines or guidelines that help HCPs organize their practices and influence how and when care is provided. When asked about barriers and enablers in sepsis management, HCPs talked about lack of awareness of policies that made it difficult to identify and manage sepsis cases. This concern was raised by few key informants that a number of HCPs working in the facility are unaware of the hospital policies. Though all the key informants noted the presence of policies and guidelines for sepsis management, only a few (6/16) key informants had detailed knowledge about the policies or guidelines related to sepsis management. The other departments in the hospital example medical ICU, surgical ICU, labor room, emergency room and inpatient wards follow different guidelines for sepsis management. This hinders the care given to patients because of no unified system or protocol exists in the facility for sepsis management.

Few of the people know the correct knowledge of sepsis. People should refresh their knowledge and there should be combined meetings of all units so we have a protocol for CVP lines, high flow oxygen administration and antibiotics. There should be a set vision for this" (KII- Senior Registrar)

For peer review only - http://bmjopen.bmj.com/site/about/guidelines.xhtml

It was also reported by health administrator of the facility that the non-performance and non-seriousness of HCPs towards their job responsibilities is an impeding factor in sepsis management. This non-performance and non-seriousness is the result of frustration and burnout caused due the HCPs workload.

"Our doctors are in a hurry to quickly complete their work and go, because they have a lot of burden" (KII- Healthcare Administrator)

All HCPs stressed on compromised quality of resources available in the facility. They reported that the quality and efficiency of antibiotics is lacking and there are hurdles in obtainability of antibiotics. This delays patients' management and the patient care process.

"The most important is the below standard antibiotics provided here" (FGD- Associate Professor OBGYN)

This is honest truth that the antibiotics we get from outside, from a good company, there is a difference in the quality and efficiency. We are not getting good results with antibiotics as we are supposed to" (KII- Senior Registrar)

HCPs also highlighted the constraints faced from the level of patients. The collection and transport of blood samples to laboratories is a complicated process. The patient's samples are transferred to laboratories by the hospital staff at the selected time of the day. If any patient's investigation is required after that fixed set time, it is transferred to laboratory through patients' attendants. Consequently, this delays patients' investigational process.

"We have developed a system that in morning, the ward boy will collect samples from each ward, it goes to university hospital which doesn't charge anything. If any sample is

For peer review only - http://bmjopen.bmj.com/site/about/guidelines.xhtml

356 *missed and sent later, we send them through patient's attendants and they are charged"*

357 *(KII-Health Administrator)*

358 HCPs also deliberated on patient's ability to afford for lab investigations. Most of the patients
359 coming to the facility belong to the low-income class group considering their socio-economic
360 background. Though LUMHS is a public health facility and a majority of services are provided
361 in the hospital without charge, there are few investigations for which patients are required to pay
362 fee for services for example blood culture and serum lactate tests.

363 *"Our patients are poor and they cannot afford investigations like culture test and serum*
364 *lactate. They are costly so people are reluctant for these blood test" (KII- Registrar)*

365 *"These investigations should be free for patients. Culture bottles are so expensive and*
366 *people are so poor that they go and throw them away" (FGD- Registrar Admin)*

367 **Clinical practice Variation**

368 a. Sepsis guidelines and documentation

369 The interview participants reported that the obstetrics and gynecology units follow Royal
370 College of Gynecology (RCOG) guidelines. The RCOG guiding principles provides information
371 about the risk factors of maternal sepsis, the basic vital signs and identification of maternal
372 sepsis, clinical features suggestive of sepsis, investigations to rule out maternal sepsis, and the
373 specific antimicrobial therapy for management [20]. Despite the presence of guidelines in the
374 hospital, the early identification and management of sepsis is a huge struggle.

For peer review only - http://bmjopen.bmj.com/site/about/guidelines.xhtml

375 *"MEOWS chart was there in RCOG guidelines and we used to do that, but as you have*
376 *these FAST-M tools, we didn't use to do this way. We used to do this very haphazardly"*
377 *(KII- Assistant Professor)*

378 The **F** in the pneumonic of FAST-M denotes fluid resuscitation. This administration of
379 intravenous fluids can be a key intervention for management of sepsis if it is associated with
380 hypotension, however, rapid fluid administration is more complex in pregnant women if there
381 are other co-existing medical problems such as eclampsia. These concerns and delays in fluid
382 administration in the existing system was identified by HCPs. This delay was because of the
383 HCPs anticipated apprehensions and concerns related to complications of fluid therapy as stated:

384 *"In existing practices, we are giving the antibiotics but this fluid therapy sometimes gets*
385 *delayed as we are concerned about development of pulmonary edema in septic patients*
386 *after giving fluids" (KII- Registrar)*

387 *" Sometimes these gynae people get worried that whether it is sepsis or cardiac issue and*
388 *whether we should give fluids or not as patient can have fluid overload" (FGD- Assistant*
389 *Professor- Medicine)*

390 Most of the study participants stated that they are following the similar procedures and
391 guidelines as provided in FAST-M bundle care tools. Yet, they identified lack of documentation
392 in the existing practices.

393 *"We do not follow the step wise procedure and documentation but we follow the same*
394 *thing as we do respiratory rate, BP, GCS and etc." (KII- Fellow-ICU)*

395 b. Individual care practices and HCP comfort levels

For peer review only - http://bmjopen.bmj.com/site/about/guidelines.xhtml

There is a hierarchy of doctors in the hospital from senior to junior level based on their qualification and experience. The hospital units are managed by Professors who are Head of Department of the units. The upper category in the hierarchy of doctors comprises of all the faculty staff including associate professors and assistant professors, the second upper category in the hierarchy covers registrar doctors, who support postgraduate residents and house officers who come for their internship program following completion of medical training. These all categories of doctors have diverse job roles for management of patients as stated:

> *"We have faculties and we have them on senior level, then we have our Registrars, PGs and Hos, so suppose senior level look for all the patients, do patients rounds and check and advice for the patients. Registrars have their assigned patients' beds. The registrars are assigned according to the number of beds present and occupied. These registrars are accompanied by PGs. Suppose, if any registrar is assigned 12 beds, she gets two PGs who can look after 6-6 beds. So the main people who are on floor are registrars and PGs who manage patients according to faculty's advice" (KII- Associate Professor)*

Within the hospital it was observed that HCPs approach towards sepsis management was not consistent. Clinical practice variation refers to patients receiving differing care depending on when, where, and by whom they are being cared for, despite evidence for best practice. One HCP noted that:

> *"Some doctors send lactate and culture test and others don't... this may be because of patient's financial affordability. And this variation is also there when we prescribe antibiotics. Every doctor has their own practice" (KII- Registrar)*

For peer review only - http://bmjopen.bmj.com/site/about/guidelines.xhtml

Some nurses voiced concerns about timely management of patients. HCPs reported that patients monitoring gets delayed based on an individual nurse's levels of comfort to monitor the patients. There are less skilled nurses in the unit to identify and assess the criticality of the patient. The novice nurses are inexpert to take care of the patients and they also lack skills towards sepsis care.

> *"Senior nurse makes the schedule and look after the labor room as well as ward because of their competencies. We have new nurses as well but it is obvious that their understanding and knowledge of the work is less than ours" (KII- Staff nurse)*

> *"We get senior and competent nurses in the morning shift because there is more work in morning shifts" (KII- Senior Registrar)*

Unit practice norms, combined with the HCPs' personal comfort, confidence, and skills, inform their practices about sepsis management. HCPs also have varying definitions and criteria for which patients are transferred to ICUs and to sort this process uninterrupted, HODs decides on the eligibility criteria for admission to ICU.

Health care provider's perceptions about FAST-M

a. Understanding of the FAST-M bundle

HCPs reported that they were informed about FAST-M bundle care tools from their head of departments who are keen to test this intervention in their local setting. Some health care providers had more opportunities to learn about the components of FAST-M bundle, but other HCPs specifically staff nurses did not know about the FAST-M tools. While all doctors reported having a baseline understanding of FAST-M tools and its components including MEOWS chart, decision tool and treatment tool, they expressed the need of additional understanding of FAST-M

For peer review only - http://bmjopen.bmj.com/site/about/guidelines.xhtml

tools before its implementation. All HCPs recommended providing additional education and training sessions to HCPs to address such gaps.

> *"Whatever HCPs are doing, they are doing at their own, they are also trained but they are not very well trained, so training will help them to manage patients well according to the guidelines" (KII- OR Doctor)*

Healthcare administrators and doctors employed at the hospital displayed their interest in support for implementation of FAST-M intervention, whereas nurses most frequently cited satisfaction with their existing practices.

> *"Our OBGYN doctors are already providing us the charts for monitoring of cesarean deliveries, for baby's monitoring and there are different charts for monitoring. We are already managing our patients well" (FGD- Nurse)*

Majority of the key-informants highlighted positive influences of implementation of FAST-M bundle care tools on existing policies of sepsis management in the hospital as one of them stated:

> *"There is no current guideline followed in the hospital and this has come as a sort of guideline that can be used for sepsis management" (KII- OR Doctor)*

b. Perceptions about significance of FAST-M

HCPs attitudes towards FAST-M implementation were positive and supportive. All HCPs shared positive perceptions about timely sepsis identification and management through classification of patients using MEOWS chart's triggers as red and yellow flags. The use of colors such as red flags and yellow flags indicating cutoff values facilitates HCPs in identifying and categorizing

For peer review only - http://bmjopen.bmj.com/site/about/guidelines.xhtml

patients. HCPs identified color demonstration in the MEOWs chart as a major enabler in identification of sepsis patients.

> *"Now we know that there is a red and yellow flag, and if patient is in severe sepsis we have to send the samples within an hour and have to give antibiotic and fluids as described in the protocol" (KII- Registrar)*

> *"It is very easy because of colors we are getting alert on red and yellow flags. This is very easy and understandable" (KII- Senior Registrar)*

HCPs believed that FAST-M tools improve knowledge of HCPs as the tools include everything related to identification and management of the patients with maternal sepsis. The flow of the tools was appreciated by HCPs and they also stated that this organized flow of FAST-M tools will save time in sepsis management.

> *"This tool provides specifications about fluid therapy and antibiotics administration with specific time. It has improved our knowledge" (KII- Nurse)*

HCPs also indicated the significance of FAST-M tools as being initiated by any healthcare provider including the nurse. There is no requirement of a doctor to initiate the bundle care tools. The staff nurses and even the trainee dispensers, who are available in the unit as helpers to staff nurses, can initiate the MEOWs chart for identification of the cases.

> *"The good thing I see in this FAST-M is that even the nurse can start this bundle care" (FGD- HOD Gynae)*

Generally, most HCPs stated that the FAST-M intervention will help in sharing tasks between HCPs and it will increase accountability of HCPs to perform their responsibilities

For peer review only

For peer review only - http://bmjopen.bmj.com/site/about/guidelines.xhtml

"It should be done because from staff till doctor everybody will be responsible for their work and will document each and every thing. We get tired of emphasizing this" (KII- ICU Fellow)

One of the KIs emphasized the quality of this tool as being non-invasive. Patients would easily accept this intervention and HCPs would not hesitate to initiate it. It can be easily accepted and implemented.

"The intervention that has been introduced, it is totally non-invasive and it is the same work that we do in our daily routine, so we will have no problems in its implementation" (KII- ICU Fellow)

All the key-informants and focus group participants articulated patients' benefits through FAST-M implementation. They emphasised that the early identification and management of maternal sepsis through the FAST-M tools may decrease patient's length of stay in hospital, and eventually decreasing the length of stay would benefit patients in providing physical, economic and psychological advantages. Ultimately, this would help in decreasing maternal morbidities and mortalities in the long run.

"…it will benefit patient that it will help in decreasing the stay of patients and their exposure will be reduced. This will reduce morbidities and mortalities in the long run" (KII- Registrar)

c. Identifying solution to the application of FAST-M

Some HCPs were doubtful of the practicality of intervention in the prolonged and continuous implementation due to resource restrictions (e.g. quality of available antibiotics, shortage of

For peer review only - http://bmjopen.bmj.com/site/about/guidelines.xhtml

501 staffing, shortage of equipment's and monitors). The inability to overcome these limitations led
502 to a common attitude that:

503 *"Nothing is sufficient from top to bottom, we try our level best to provide but we do not*
504 *have monitors, we have hurdles for lab investigations, there are issues of availability of*
505 *nurses and antibiotics, there are many technical gaps" (KII- Registrar Admin)*

506 All respondents suggested that in order to strengthen the significance to FAST-M bundle for
507 early identification of sepsis, the inclusion of the variable of oxygen saturation in the MEOWS
508 chart, with appropriate cut off values, would be important. This was because pulse oximetry is
509 now available routinely in the unit and may be an important indicator of clinical deterioration.
510 This feedback was consistently given by all HCPs.

511 *"Oxygen saturation is mandatory to include in the MEOWs chart for monitoring of*
512 *patient" (FGD- Assistant Professor- Medicine)*

513 It was informed through HCPs working in the medicine unit that sepsis guidelines followed in
514 their unit include an addition of steroid therapy and inotrope support for sepsis management.

515 *"You should include support because sometime when we give fluids and antibiotics, but*
516 *still patient is not maintaining the blood pressure because most of the times septic*
517 *patients arrives late, so you should include source plus support in S. so both of the things*
518 *will be included. Because support is the most important" (FGD- Assistant Professor-*
519 *Medicine)*

520 All HCPs agreed over the use of ceftriaxone as first choice of antibiotics in FAST-M treatment
521 bundle based on its cost and availability for patients.

For peer review only - http://bmjopen.bmj.com/site/about/guidelines.xhtml

"We give Ceftriaxone straight away as it is freely available. We give 2g Ceftriaxone and for those patients whose culture is sent, we wait for their blood culture reports to change antibiotics accordingly. Otherwise, our patient mostly responds to ceftriaxone" (KII- Senior Registrar)

Few participants specified that they use Piperacillin/tazobactam and meropenem for management of the confirmed cases of sepsis due to their beneficial results in such patients, yet the patients pay out of pocket for the cost of these antibiotics. Thus, Meropenem and Piperacillin/Tazobactam were proposed as second choice of antibiotics due to their availability and cost.

"…sometimes when we do not have availability of meropenem so we give ceftriaxone to the patients, which is easily available free of cost for patients" (KII- Senior Registrar)

HCPs also suggested involving nursing interns and trainee dispensers who come for their training and work without wages. The involvement of nursing interns and trainee dispensers would reduce the problem of shortage of staffing in the unit and they would be employed to implement the FAST-M intervention without added investment for human resources.

"We get one or two girls from BScN programme, but we can talk to the dean in account and there are many people who can help us with this" (FGD- Health Administrator)

The focus group participants identified the need of increasing awareness which is the key to implementation of the FAST-M intervention. The stakeholders emphasized understanding of HCPs about the significance of FAST-M bundle care tools as a key to effective implementation in future. One of the group participants suggested:

For peer review only - http://bmjopen.bmj.com/site/about/guidelines.xhtml

"We can make big boards and we can involve everyone and give them awareness. And we can provide examples to them that how it was implemented in past in different setting showing good outcomes" (FGD-HOD Gynae)

Moreover, the inclusion of MEOWs charts in patients' Medical Record files of the hospital was emphasized by every group member involved in the discussion.

"We will include MEOWS chart in all patients' files so our doctors can easily record the findings on MEOWS chart which will alert them about patient's condition" (FGD- HOD Gynae)

HCPs readiness for FAST-M implementation

The HCPs readiness towards FAST-M intervention started with the drive of identification of requirements for FAST-M adaptation and concluded with consensus building of HCPs for its implementation.

a. Understanding and identifying gaps

HCPs acknowledged that successful implementation of the FAST-M intervention would require health care facility to be well-equipped, including both the availability of equipment and trained health care providers. Other key challenges to the successful implementation of FAST-M intervention are related to logistics, including shortage of human resources and inadequate funds for procuring monitors for assessments, antibiotics and lab investigations. One of the most frequent concerns around FAST-M implementation included the need to train HCPs including doctors, nurses, and auxiliary support staff to enable them to set up and sustain the services. Further, study participants suggested that a multidisciplinary approach would be useful to ensure that all professionals including the team of doctors, nurses, administrators from different units

For peer review only - http://bmjopen.bmj.com/site/about/guidelines.xhtml

For peer review only

e.g. medicine, intensive care units, labor room, laboratory and operating room are working together for the successful implementation of FAST-M.

> *"In team, one person should be from administration, to who if we complain related for our hurdles and queries, so he can work on them, one person should be from laboratory, one should be from nursing staff and one should be from doctors, who can take all the things to higher levels and work on them" (KII- Registrar Admin)*

Healthcare providers argued that there are high costs associated with the implementation of FAST-M intervention. Providers further explained that high costs of laboratory investigations would be a limiting factor as it would cause additional anxiety of financial burden to the patients. On the other hand, a few health professionals confirmed that costs would not be a major concern if there was a buy-in from hospital administration for the patient's requirements. HCPs mentioned that the initial investments may be higher for procuring required equipment like monitors and apparatus required for monitoring of patients.

> *"Ceftriaxone is easily available in our hospital, but we are not sure about its quality. But for the critical patients if we see any red flags, we can arrange their requirements from our donations. In our unit, we are doing this for critical patients" (FGD-HOD-Gynae)*

b. Consensus building for FAST-M implementation

The focus group participants displayed readiness for implementation of FAST-M tool in their local context by developing consensus on resolutions and approaches to the perceived challenges they could encounter during the implementation. The focus group discussion provided the opportunity to reflect on the anticipated challenges and how they may be able to successfully implement in their setting with the available resources. HCPs decided to implement

For peer review only

For peer review only - http://bmjopen.bmj.com/site/about/guidelines.xhtml

FAST-M intervention in their setting and they also acknowledged the importance of a training program for HCPs to implement FAST-M bundle care tools in their setting. It was recognised that the FAST-M protocol comprises similar practices but in an organized and structured way, and was well-regarded by all HCPs. They valued the implication of FAST-M bundle as stated:

"We are already doing these all things except documentation so it will be easy to apply. You know the guidelines, you have got an algorithm then it would be difficult to miss any patient. So it's a very good thing and this can be implemented. We have everything but there should be training and if you give that it would be easy to implement: (FGD- Associate Professor- Medicine)

Discussion

Our findings revealed several potential facilitators for the uptake of FAST-M intervention. Firstly, the HCPs had highly favorable perceptions regarding the use of FAST-M bundle care tools. The major advantage identified was illustration of colored codes in the MEOWs chart such as red and yellow flags that assists in categorization of patients according to severity of their symptoms. The early identification of patients with maternal sepsis through MEOWs chart facilitates timely management of patients using decision and treatment tools. All HCPs acknowledged the FAST-M bundle care tools as easy to use as they do not require any invasive procedures to identify suspected maternal sepsis cases and trigger appropriate actions. Secondly, the HCPs deliberated about long-term improvement in patient's health outcomes through the use of FAST-M intervention such as decrease in length of patients' stay at the hospital, and improvement in maternal morbidities and mortalities overall.

For peer review only - http://bmjopen.bmj.com/site/about/guidelines.xhtml

Our study findings identified that the shortage of health care providers hindered many aspects of sepsis care delivery, and may be a critical barrier to any intervention. As the hospital provides free of charge care to patients, there is high influx of patients in the facility. This high volume of patients' increases workload on health care providers and eventually the shortage of health care workers is associated with adverse patient's outcomes and comprised quality in patient care [21]. Therefore, all the study participants suggested involving nursing interns, trainee dispensers and other available human resource to reduce doctors' and nurses' workload through shared responsibilities and employing a task-sharing approach. The approach of task sharing of specialists with trained non-specialist workers has provided positive outcomes in improvement of patient care, reduced morbidity and mortality rates and cost-effectiveness [22].

Accordingly, a training programme has been planned as part of the implementation of the FAST-M intervention so all HCPs providers have the required knowledge to manage sepsis cases according to the FAST-M approach, making practice uniform across teams in the facility and ensure sustainability of FAST-M intervention as a long term benefit for patients.

The source identification denoted as 'S' in the FAST-M bundle requires a detailed history and examination to identify the infection source along with the targeted further investigations. The training programme will provide an opportunity to improve this aspect, including the significance of taking a detailed history and examination and documenting them. This is very important to provide quality care and to help health care providers to plan a patient's treatment to maintain the continuum of care [23].

The FAST-M implementation in districts of Malawi provided useful example of effective implementation where champions played a significant role in implementing FAST-M intervention, and their contribution for intervention provided day-to-day oversight of healthcare

For peer review only - http://bmjopen.bmj.com/site/about/guidelines.xhtml

practitioners' practice [10]. Our study findings suggest that the clinical practice variations among healthcare providers is a potential major hindering factor in implementation of FAST-M intervention, and yet we decided to select maternal sepsis champions. These champions could potentially standardise the practices for the management of maternal sepsis in all the departments managing such cases. To continue to strengthen the implementation of this intervention, champions will be selected during training programme based on the consensus of healthcare providers involved in training of FAST-M intervention.

Moreover, the HCPs were concerned about the compromised quality of available resources such as antibiotics and laboratory investigations which voiced their uncertainty to support FAST-M intervention. They felt that the hospital's environment and the quality of available resources did not support patients' clinical management. It was identified that the hospital system set for laboratory investigations is lengthy and time consuming.

While, the quality of health services within clinical setting is imperative to provide effective care to the patients [24]. Study findings also suggest that the treatment cost adds financial burden of patient and leads to discontinuation of medical treatment [25].Thus, the practicability of intervention depends on the facility environment, availability of resources and its affordability for implementation and the readiness of 'healthcare administrators' who are accountable for provision of healthcare supplies. The role of healthcare administrators in upgrading the system is quite significant to avoid barriers to implementation. Hence, the healthcare administrators provided assurance for provision of supplies and resources as a stance to reduce maternal sepsis rate at their healthcare setting and will be fully included in the implementation process, including the training and champion network.

For peer review only - http://bmjopen.bmj.com/site/about/guidelines.xhtml

During the development of the FAST-M bundle through a modified Delphi process, oxygen saturation was mostly perceived as of reasonable importance. Though, the feasibility of implementing this element in low-resource settings limited its usefulness due to the non-availability of pulse oximeters at that time in many low-resource settings [10]. However, considering the outbreak of COVID-19 infection and the availability of pulse oximeters at the study site, it was recommended to include oxygen saturation in the MEOWs chart to determine patient's clinical condition. The inclusion of oxygen saturation in the MEOWs chart is considered important based on the existing sepsis management practices of the facility. Moreover, the element of oxygen saturation is a significant indicator in identification of patients' clinical condition. Therefore, the supplementary element of oxygen saturation has been added to the bundle care tools prior to its implementation (Supplemental file-5).

Some specialists raised consideration of broadening the bundle to include more comprehensive sepsis care including consideration of steroid therapy and inotrope support. As part of the adaptation process this issue was fully discussed with a range of local and international experts from gynecology and intensive care fields and it was decided that these aspects would be most appropriate only for specialist doctors, normally in an ICU environment, so would not be suitable for inclusion in the first response bundle. However, management of patients using steroids would be emphasized during training program to delineate its role in management of COVID-19 as a distinct situation from other bacterial causes of maternal sepsis to ensure rational and evidence based steroid use.

Antibiotics administration is one of the easily available, free of cost and important components of FAST-M treatment bundle for sepsis management. The FAST-M treatment bundle applied in the earlier study conducted in Malawi [10] was therefore of the important. We explored

For peer review only - http://bmjopen.bmj.com/site/about/guidelines.xhtml

healthcare providers' views regarding use of antibiotics in their local setting for treatment of maternal sepsis. It was identified that Ceftriaxone is easily available free of cost to patients and it provides positive results in treatment of sepsis. Thus, it was agreed to use ceftriaxone as first choice of antibiotics in FAST-M treatment bundle. Moreover, it was also acknowledged that Piperacillin/tazobactam and meropenem are used for treatment of confirmed sepsis cases due to their beneficial results, though the patients pay out of pocket for the cost of these antibiotics. Thus, Meropenem and Piperacillin/Tazobactam were proposed as second choice of antibiotics due to their availability and cost. The Malawian version of FAST-M treatment bundle was therefore modified for antibiotic guidelines (Supplemental file-5).

The importance of an explicit sepsis care policy was discovered during interviews and focus group discussion to assist in standardising infection regulations in the hospital. It was identified that the FAST-M intervention can serve as a guiding policy to provide evidence-based information to support clinical decision-making. Therefore, a unified system of FAST-M intervention for sepsis care in the facility for maternal patients can serve as a standard tool for maternal sepsis management.

The major strength of this study is the use of CFIR that guided the researchers' focus, starting with observations and documenting from a broad health systems and programme implementation perspective, becoming more specific in the later performed interviews and focus group discussion. Moreover, participation of HCPs from several levels to ask their feedback on the research question, and by interviewing HCPs about their experiences helped in gaining better insights about their practices and perceptions. Yet, this study was carried out and will be implemented in one setting only. Future studies are required to explore feasibility of implementing FAST-M bundle in other low-resource settings of Pakistan.

For peer review only - http://bmjopen.bmj.com/site/about/guidelines.xhtml

We believe that it is possible to implement the FAST-M intervention in low-resource settings of Pakistan and we recommend several strategies to address the challenges facilities may face in their local context. The hospital, leadership and HCPs require collaboration to work as a multidisciplinary team to advance sepsis management practices and understand its implications. This could be achieved through development and dissemination of FAST-M intervention as a sepsis management guideline in the facility.

The distribution of supportive resources to provide education to all HCPs including doctors, nurses and healthcare administrators about FAST-M tools is required to increase knowledge and awareness of FAST-M bundle. Also, facilities will require selected champions for implementation of the FAST-M intervention.

Overall, bundle care tools have the potential to enhance improvements in sepsis care. However, the implementation challenges posed by these bundles should be examined, especially in low-resource settings, where facilities and services have not yet flourished.

We identified facilitators and barriers for implementation of this intervention from only one of the facilities in Pakistan selected as our study site. Future research is needed to understand how implementation of this adapted FAST-M intervention works when implemented as part of care, and to rigorously evaluate its effectiveness and key implementation outcomes such as the sustainability of the intervention.

Conclusion

The FAST-M maternal sepsis bundle has the potential to be used as an integrated strategy for early recognition and management of maternal sepsis in low resource health settings in Pakistan.

For peer review only - http://bmjopen.bmj.com/site/about/guidelines.xhtml

We found several barriers and facilitators for its implementation and suggested key adaptations to the intervention which we perceive will help address these barriers.

Based on this formative research, the FAST-M tools and implementation approach in their adapted format will be implemented in the selected health facility and mixed-methods research conducted to assess the feasibility of implementing these adapted tools as part of the health care system in Pakistan.

Data availability statement

The datasets were collected and analyzed and can be made available from the corresponding author on reasonable request

Ethics statements

Patient consent for publication

Not required

Ethical approval

Ethical approval for this study was obtained from the LUMHS hospital [REC/-886, 4-87], Aga Khan University Ethical Review Committee [2019-2061-7102] and National Bioethics Committee [515/20/]. Participants will be asked to provide written consent to indicate their willingness to participate. Voluntary participation and the right to ask any questions and to decline participation at any time will be emphasized during the data collection.

Acknowledgements

For peer review only - http://bmjopen.bmj.com/site/about/guidelines.xhtml

We would like to acknowledge health officials and individuals: Dr. Anna Blennerhassett (for her expert contributions to the project), Dr. Zulfiqar Shah, Dr. Madiha Shah, Dr. Imran Karim Shaikh, Dr. Mumtaz Lakho, Dr. Fouzia Sheikh, Mr. Sattar Jatoi, Dr. Nargis and Dr. Sabrina (for providing assistance, contact numbers and permissions for data collection at field sites). We would also like to acknowledge Dr. Sadia Shakoor (Associate Professor Section of Microbiology, Department of Pathology and Laboratory Medicine, Aga Khan University Hospital) in providing information and recent statistics related to use of antibiotics in Pakistan.

For peer review only

For peer review only - http://bmjopen.bmj.com/site/about/guidelines.xhtml

References

1. Say L, Chou D, Gemmill A, Tunçalp Ö, Moller AB, Daniels J, Gülmezoglu AM, Temmerman M, Alkema L. Global causes of maternal death: a WHO systematic analysis. The Lancet Global Health. 2014 Jun 1;2(6):e323-33.
2. Bonet M, Pileggi VN, Rijken MJ, Coomarasamy A, Lissauer D, Souza JP, Gülmezoglu AM. Towards a consensus definition of maternal sepsis: results of a systematic review and expert consultation. Reproductive health. 2017 Dec;14(1):67.
3. Peters DH, Adam T, Alonge O, Agyepong IA, Tran N. Implementation research: what it is and how to do it. Bmj. 2013 Nov 20;347.
4. World Health Organization, Unicef. Trends in maternal mortality: 1990 to 2013: estimates by WHO, UNICEF, UNFPA, The World Bank and the United Nations Population Division: executive summary. World Health Organization; 2014.
5. van Dillen J, Zwart J, Schutte J, van Roosmalen J. Maternal sepsis: epidemiology, etiology and outcome. Current opinion in infectious diseases. 2010 Jun 1;23(3):249-54.
6. Khalid S Khan, Daniel Wojdyla, Lale Say, A Metin Gülmezoglu, Paul FA Van Look,WHO analysis of causes of maternal death: a systematic review,The Lancet,Volume 367, Issue 9516,2006,Pages 1066-1074,ISSN 0140-

For peer review only

For peer review only - http://bmjopen.bmj.com/site/about/guidelines.xhtml

6736,https://doi.org/10.1016/S0140-6736(06)68397 (https://www.sciencedirect.com/science/article/pii/S0140673606683979)

7. Lissauer D, Cheshire J, Dunlop C, Taki F, Wilson A, Smith JM, Daniels R, Kissoon N, Malata A, Chirwa T, Lwesha VM. Development of the FAST-M maternal sepsis bundle for use in low-resource settings: a modified Delphi process. BJOG: An International Journal of Obstetrics & Gynaecology. 2020 Feb;127(3):416-23.

8. Seymour CW, Gesten F, Prescott HC, Friedrich ME, Iwashyna TJ, Phillips GS, Lemeshow S, Osborn T, Terry KM, Levy MM. Time to treatment and mortality during mandated emergency care for sepsis. New England Journal of Medicine. 2017 Jun 8;376(23):2235-44.

9. Taki F. *The development of a care bundle to improve the initial management of maternal sepsis for use in low and lower-middle income countries* (Doctoral dissertation, University of Birmingham).

10. Cheshire J, Jones L, Munthali L, Kamphinga C, Liyaya H, Phiri T, Parry-Smith W, Dunlop C, Makwenda C, Devall AJ, Tobias A. The FAST-M complex intervention for the detection and management of maternal sepsis in low-resource settings: a multi-site evaluation. BJOG: An International Journal of Obstetrics & Gynaecology. 2021 Feb 4.

11. Madhudas C, Khurshid F, Sirichand P. Maternal morbidity and mortality associated with puerperal sepsis. Journal of Liaquat University of Medical and Health Sciences. 2011 Sep 1;10(03):121.

12. Iftikhar R. A study of maternal mortality. J Surg Pak.(Int.). 2009 Oct;14(4):176-8.

For peer review only

13. Arulkumaran N, Singer M. Puerperal sepsis. Best Practice & Research Clinical Obstetrics & Gynaecology. 2013 Dec 1;27(6):893-902.

14. Shamshad S, Shamsher S, Rauf B. Puerperal sepsis—still a major threat for parturient. Journal of Ayub Medical College Abbottabad. 2010 Sep 1;22(3):18-22.

15. Begum S, Aziz-un-Nisa BI. Analysis of maternal mortality in a tertiary care hospital to determine causes and preventable factors. J Ayub Med Coll Abbottabad. 2003;15(2):49-52.

16. Reinhart K, Kissoon N. Sepsis Guidelines for Pakistan. Anaesth Pain & intensive Care. 2015 Apr 1;19(2):105-7.

17. Damschroder L, Hall C, Gillon L, Reardon C, Kelley C, Sparks J, Lowery J. The Consolidated Framework for Implementation Research (CFIR): progress to date, tools and resources, and plans for the future. InImplementation science 2015 Dec (Vol. 10, No. 1, pp. 1-1). BioMed Central.

18. Peters DH, Adam T, Alonge O, Agyepong IA, Tran N. Implementation research: what it is and how to do it. Bmj. 2013 Nov 20;347.

19. Birken SA, Powell BJ, Presseau J, Kirk MA, Lorencatto F, Gould NJ, Shea CM, Weiner BJ, Francis JJ, Yu Y, Haines E. Combined use of the Consolidated Framework for Implementation Research (CFIR) and the Theoretical Domains Framework (TDF): a systematic review. Implementation science. 2017 Dec;12(1):1-4.

For peer review only - http://bmjopen.bmj.com/site/about/guidelines.xhtml

20. Royal College of Obstetricians and Gynaecologists. Bacterial Sepsis in Pregnancy. Green-top Guideline No.64a. London: RCOG; 2012 [https://www.rcog.org.uk/en/guidelines-research-services/guidelines/gtg64a/].

21. Padmanathan P, De Silva MJ. The acceptability and feasibility of task-sharing for mental healthcare in low and middle income countries: a systematic review. Social science & medicine. 2013 Nov 1;97:82-6.

22. Dhar RL. Service quality and the training of employees: The mediating role of organizational commitment. Tourism Management. 2015 Feb 1;46:419-30

23. Hess CT. Documentation drivers for optimal patient outcomes. Nursing2020. 2017 Aug 1;47(8):69.

24. Sajid MS, Baig MK. Quality of health care: an absolute necessity for public satisfaction. International Journal of Health Care Quality Assurance. 2007 Sep 11.

25. Shaikh I, Singh S. On the examination of out-of-pocket health expenditures in India, Pakistan, Sri Lanka, Maldives, Bhutan, Bangladesh and Nepal. Business: Theory and Practice. 2017 Mar 5;18:25.

For peer review only - http://bmjopen.bmj.com/site/about/guidelines.xhtml

Footnotes

Authors' contributions

SI, DL, RB & LS conceptualized the design of the study and creation of data collection tools. RS, RR, SK assisted in data collection from field site. SI, RB, BK & GK managed data collection and interpretation. SI and BK carried out the analysis and wrote the initial manuscript. All authors provided input during the interpretation of the data and revising of the manuscript. DL, AC, RB, JS, CD provided feedback on the first draft. SI & BK edited and wrote the final draft. The authors read and approved the final manuscript.

Funding

The funding for this project is provided by the University of Birmingham, University of Liverpool, National Institute of Health Research and Bill and Melinda Gates Foundation.

Competing interests

The authors declare that they have no competing interests.

For peer review only - http://bmjopen.bmj.com/site/about/guidelines.xhtml

For peer review only

For peer review only - http://bmjopen.bmj.com/site/about/guidelines.xhtml

COREQ (COnsolidated criteria for REporting Qualitative research) Checklist

A checklist of items that should be included in reports of qualitative research. You must report the page number in your manuscript where you consider each of the items listed in this checklist. If you have not included this information, either revise your manuscript accordingly before submitting or note N/A.

Topic	Item No.	Guide Questions/Description	Reported on Page No.
**Domain 1: Research team and reflexivity**			
Personal characteristics			
Interviewer/facilitator	1	Which author/s conducted the interview or focus group?	
Credentials	2	What were the researcher's credentials? E.g. PhD, MD	
Occupation	3	What was their occupation at the time of the study?	
Gender	4	Was the researcher male or female?	
Experience and training	5	What experience or training did the researcher have?	
Relationship with participants			
Relationship established	6	Was a relationship established prior to study commencement?	
Participant knowledge of the interviewer	7	What did the participants know about the researcher? e.g. personal goals, reasons for doing the research	
Interviewer characteristics	8	What characteristics were reported about the inter viewer/facilitator? e.g. Bias, assumptions, reasons and interests in the research topic	
**Domain 2: Study design**			
Theoretical framework			
Methodological orientation and Theory	9	What methodological orientation was stated to underpin the study? e.g. grounded theory, discourse analysis, ethnography, phenomenology, content analysis	
Participant selection			
Sampling	10	How were participants selected? e.g. purposive, convenience, consecutive, snowball	
Method of approach	11	How were participants approached? e.g. face-to-face, telephone, mail, email	
Sample size	12	How many participants were in the study?	
Non-participation	13	How many people refused to participate or dropped out? Reasons?	
Setting			
Setting of data collection	14	Where was the data collected? e.g. home, clinic, workplace	
Presence of non-participants	15	Was anyone else present besides the participants and researchers?	
Description of sample	16	What are the important characteristics of the sample? e.g. demographic data, date	
Data collection			
Interview guide	17	Were questions, prompts, guides provided by the authors? Was it pilot tested?	
Repeat interviews	18	Were repeat inter views carried out? If yes, how many?	
Audio/visual recording	19	Did the research use audio or visual recording to collect the data?	
Field notes	20	Were field notes made during and/or after the inter view or focus group?	
Duration	21	What was the duration of the inter views or focus group?	
Data saturation	22	Was data saturation discussed?	
Transcripts returned	23	Were transcripts returned to participants for comment and/or	

For peer review only

Topic	Item No.	Guide Questions/Description	Reported on Page No.
		correction?	
**Domain 3: analysis and findings**			
Data analysis			
Number of data coders	24	How many data coders coded the data?	
Description of the coding tree	25	Did authors provide a description of the coding tree?	
Derivation of themes	26	Were themes identified in advance or derived from the data?	
Software	27	What software, if applicable, was used to manage the data?	
Participant checking	28	Did participants provide feedback on the findings?	
Reporting			
Quotations presented	29	Were participant quotations presented to illustrate the themes/findings? Was each quotation identified? e.g. participant number	
Data and findings consistent	30	Was there consistency between the data presented and the findings?	
Clarity of major themes	31	Were major themes clearly presented in the findings?	
Clarity of minor themes	32	Is there a description of diverse cases or discussion of minor themes?	

Developed from: Tong A, Sainsbury P, Craig J. Consolidated criteria for reporting qualitative research (COREQ): a 32-item checklist for interviews and focus groups. *International Journal for Quality in Health Care*. 2007. Volume 19, Number 6: pp. 349 – 357

Once you have completed this checklist, please save a copy and upload it as part of your submission. DO NOT include this checklist as part of the main manuscript document. It must be uploaded as a separate file.

For peer review only

Supplementary file 2

Informed Consent

Title of study:	Extension of the FAST-M maternal sepsis bundle in Pakistan, a feasibility study
Chief Investigator:	Professor David Lissauer
Site:	Liaquat University of Health Sciences Pakistan
Site Principal Investigator:	Dr Sheikh Irfan Ahmed
Site CO-PI's	Dr Lumaan Sheikh, Dr Raheel Sikandar and Dr. Rubina Barolia
Ethics approval:	AKU ERC-2019-2061-7102,<br>LUMHS/ REC/-886, 4-87/NBC-515/20/
Affiliated organizations:	University of Birmingham, University of Liverpool & Aga Khan University Hospital Pakistan & Liaquat University of Medical & Health Science, Jamshoro.

We would like to invite you to take part in this research study. Before you decide, we would like you to understand the study, why the research is being done and what this part of the study involves for you. One of the team will explain the study to you and answer any questions you may have.

Part 1: Purpose of the study

What is the purpose of the overall study?

We are developing an intervention that we hope will improve the care of patients with maternal sepsis around the world. Sepsis is when an infection has become severe enough to lead to organ dysfunction and become life threatening.

The intervention is composed of three things:

1. The MEOWS (Maternal Early Warning Scores) chart tool to help you monitor patient's observations and help detect maternal sepsis

2. The FAST-M sepsis "bundle", to help ensure fast, consistent and effective treatment of maternal sepsis

3. A training day to learn to use the tools to help recognize and treat maternal sepsis

For peer review only

We hope that this intervention will make caring for patients with maternal sepsis easier. This study aims to discover whether it is possible to introduce this intervention into Pakistan healthcare facilities.

We hope to try and understand the good and bad aspects of the bundle to try and make it more user friendly and effective. We hope that using this bundle will make caring for patients with maternal sepsis easier.

In order to achieve this we hope to:

1. Understand your current experiences in managing maternal sepsis at your hospital

2. Understand what you thought was good and bad about the intervention.

3. Understand ways to improve the intervention.

4. Evaluate the intervention to see if it improves care in your hospital.

We hope you will be willing to participate in all of the activities for the study mentioned above.

Why have I been invited to participate?

You have been invited to participate because you work in maternity care and we would like to understand your experiences of maternal sepsis and the proposed intervention.

What will I have to do if I take part?

You will be interviewed several times over a period of six to eight months. Sometimes these will be one on one interviews and sometimes in groups. The interviews will be in English and take up to an hour. The interview will take place at or close-by to your place of work, at a time that is convenient to you. The interview will be audio-recorded to allow us to analyse the information you give us. Some or all of the information will be transcribed word for word. This information will be used in several ways – all of which will be anonymous so that your identity is not disclosed. The table describes how your information will be used.

At the start of the study the information that you give us will be used to understand current practice at your hospital for the management of maternal sepsis. During the study the information that you give us will be used to discover the good and bad aspects of the intervention and how it could be improved to make it easier for you to manage patients with maternal sepsis. This will help us decide whether the intervention is a success or not. Some of the information you give us, including word for word extracts, will be used in the final project report, which may also be published in a journal.

Do I have to take part?

It is completely up to you to volunteer to be interviewed and it will have no effect upon your work. We will describe the study and go through this information sheet with you. If you decide to take part, we will then ask you to sign a consent form.

What are the possible disadvantages and risks of taking part?

For peer review only

Before participating you should consider that we will be asking you about your experiences, opinions, beliefs and feelings in relation to the intervention. We are interested in finding out about the positive things that help you do your work and anything that hinders your work. Although unlikely, there is a possibility that you might feel upset when answering these questions during the interview. If this was to occur, you would be able to take a break or continue another day.

There will be an opportunity at the end of the interview for you to consider whether there is anything that you have discussed that you would prefer not to be included in the transcript. The transcript will also be made available to you to review by email if you would like. As a participant you are free to withdraw during the interview and up to a month afterwards, without giving a reason.

What are the possible benefits of taking part?

We hope that you will find the experience interesting and enjoyable. The information we collect from this study will be used to help us make the intervention the best it can be. Your interview will also be very important in evaluating the interventions effects at your hospital and its potential usefulness in the management of maternal sepsis.

What are the financial considerations of taking part in this study?

We would like to provide you a token of thanks at the end of the interview for providing your time and information with us.

What if there is a problem?

Any complaint about the way you have been dealt with during the study or any possible difficulty you might suffer will be addressed. Information on this is given in Part 2.

Will my taking part in the study be kept confidential?

We will follow ethical practice and all information about you will be handled in confidence. Further details are included in Part 2.

This completes part 1. If the information in Part 1 has interested you and you are considering participation, please read the additional information in Part 2 before making any decision.

Part 2: Conduct of the study

What will happen if I don't want to carry on with the study?

You may withdraw from the study without giving a reason. If you chose to withdraw from the study during or up to one month after your interview, we might ask you whether we can use the information you have given us, such as your interview answers. If you don't want to carry on with the study but you give us permission to use the information already collected, we will proceed to keep it securely. If you wish to withdraw and don't want your data to be used for the study, we will delete any recordings and destroy transcript files.

What if there is a problem?

For peer review only

If you have a concern about any aspect of this study, you can speak to the researchers, who will do their best to answer your questions. Their contact details are on the last page.

Will my taking part in this study be kept confidential?

The study will take place at your workplace, and for this reason it is possible that other work colleagues will be aware of your participation. However, we will follow these procedures for collecting, storing, processing and destroying information about you to ensure your confidentiality and safeguard your data:

- The recording of any information you give us during your interview will be stored in a password protected file and only authorised people will have access to it. This will help prevent people identifying your voice.
- The data transcribed from recordings will be stored securely on a computer with access restricted by a password. Transcripts will not include names or locations. Consent forms and printed transcripts will be kept in a locked cabinet, only accessible to authorised researchers.
- Data collected will be used for this study but, with your permission, might also be retained to include it anonymously in future studies.
- The identifiable data will be retained for the duration of the study and will be disposed of securely (i.e. shredding documents).

As a participant, you would have the right to check the accuracy of data held about you and correct any errors.

What will happen to the results of the research study?

The researchers will write a report outlining the results of this study. You will not be identified in any report, presentation or publication, however extracts from your interviews may be reproduced. The results will be used to inform local practice and a future possible larger scale trial of the intervention. If you are interested in the outcome of the research, then a summary of the findings can be sent to you via email and if you wish you will be invited to attend a feedback day at the end of the project.

Who is organizing the research

This study is being carried out by the University of Birmingham, UK. University of Liverpool, UK and Aga Khan University Hospital(AKUH), Pakistan The research team is being led by Dr David Lissauer, Dr Lumaan Sheikh and Dr Sheikh Irfan is the researcher conducting this part of the study.

Who has reviewed the study?

This study has been reviewed by the National Bioethics Committee Pakistan and College Research Ethics Committee in AKUH.

Contact details:

For peer review only

Dr Sheikh Irfan Ahmed, Senior Instructor, AKUH National stadium road, Karachi Email: sheikh.irfan@aku.edu Telephone number: +92-021-34864650

Dr David Lissauer Lecturer in Maternal and Fetal Medicine, University of Birmingham, UK Email: David.Lissauer@liverpool.ac.uk

Dr Lumaan Sheikh Associate Professor, AKUH National stadium road, Karachi Email: lumaan.sheikh@aku.edu Telephone number: +92-021-34864641

Dr Raheel Sikandar Professor, Liaquat University of Medical & Health Sciences, Jamshoro Email: pgmc@lumhs.edu.pk Telephone number: + 92-22-9213322

Please keep this information sheet for your own records.

Dr Rubina Barolia, Associate Professor and Assistant Dean, School of Nursing, AKU, Email: rubina.barolia@aku.edu Telephone number: +92-021-34865446

Bakhtawar Khowaja, Research Coordinator, AKUH National stadium road, Karachi Email: Bakhtawar.hanif@aku.edu Telephone number: +92-021-34864626

PLEASE INITIAL THE BOXES IF YOU AGREE WITH EACH SECTION:

1. I have read the information sheet version 2.5 for the above study and have been given a copy to keep. I have had the opportunity to consider the information, ask questions and have had these answered satisfactorily. ☐

2. I understand that my participation is voluntary and that I am free to withdraw up to one month after my participation without giving any reason. ☐

3. I agree to be interviewed for research in this study. I agree to my interview being audio-recorded and I understand that transcripts will be anonymised. I understand that participating in the interview for this research is voluntary and that I am free to withdraw my approval for use of the audio recordings and transcripts up to one month after my participation. ☐

4. I understand that anonymised sections of data collected during the study, may be looked at by individuals from regulatory authorities in the UK or Pakistan. I give permission for these individuals to have access to my anonymised transcript. ☐

5. I understand that the researchers might publish an article in a journal with the results of this study. I give permission for my transcripts to be used for this purpose. I understand that these transcripts will be anonymised. ☐

6. I know how to contact the research team if I need to. ☐

7. I understand that I may terminate the interview at any time ☐

8. I am happy for information about me related to the study being stored on a password protected computer system, which will be backed-up in a separate location to keep this

For peer review only

information safe. Data collected will be used for this study but, might also be retained to include it anonymously in future studies ☐

9. I agree to participate in this study. ☐

SIGNATURES:

Participant Name and Surname ____________________ Date________________

Signature ____________________________

Researcher Name and Surname ___________________________ Date ____________________

Signature________________________________

For peer review only

Supplementary file 1

Interview Guide

Intervention Characteristics

1. What do you know about the intervention or its implementation?
2. How different is this intervention from your existing practices?
3. What kind of information or evidence are you aware of that shows whether or not the intervention will work in your setting?
4. What kinds of changes or alterations do you think you will need to make to the intervention so it will work effectively in your setting?
   - Do you think you will be able to make these changes? Why or why not?
5. What is your perception of the bundling of the intervention for implementation and quality of the supporting materials? Prompts: format, design, user-friendly. Duration, scope, intricacy and number of steps

Outer Setting

6. How do you think the individuals served by your organization will respond to the intervention?
7. What barriers will the individuals served by your organization face to participating in the intervention?
8. What kind of local, state, or national performance measures, policies, regulations, or guidelines might be important in influencing how this intervention can be implemented?

Inner Setting

9. Can you describe how the intervention will be integrated into current processes?
10. What are your current guidelines to assess and manage patients with maternal sepsis? Probes: tool, framework or guidelines for maternal sepsis, lactate test
11. What is your knowledge about importance of lactate test and what is your current practice about lactate testing? Probes: implications for lactate test, guidelines for lactate test
12. What is your current patient to doctor and patient to nurse’s ratio in your setting?

For peer review only - http://bmjopen.bmj.com/site/about/guidelines.xhtml

13. Explain the role of doctors and nurses in management of maternal sepsis in your organization. Which cadre is responsible for care and at what level of care? Probes: nurses, doctors, technicians and other health care cadres
14. Other than human resources, what resources are utilized in management of maternal sepsis in your hospital?
15. Do you expect to have sufficient resources to implement and administer the intervention?
    - [If no] What resources will not be available? Probes: human resource, equipments, critical units etc
16. Do you feel the training planned for you will prepare you to carry out the roles and responsibilities expected of you?
    - What are the positive aspects of planned training? What is missing?

Characteristics of Individuals

17. How do you feel about the intervention being used in your setting?
18. Do you think the intervention will be effective in your setting? Why or why not?

Process

19. Who will lead implementation of the intervention?
20. Are there people in your organization who are likely to champion (go above and beyond what might be expected) the intervention?
    Prompts: Position of these champions have in your organization?
21. How do you think they will help with implementation?

MODIFIED EARLY OBSTETRIC WARNING CHART (MEOWS CHART)

FAST-M

Contact clinical decision maker if patient triggers **ONE RED** or **TWO YELLOW** flags at any time.

Patient		Patient ID		DOB/Age	

WRITE **VALUES** IN BOXES PROVIDED

		Date
		Time
		Initials
Respiratory rate (breaths per minute)	25 or more	RED
	21 - 24	YELLOW
	11 - 20	NORMAL
	10 or less	RED
Temperature (°C)	38 or more	YELLOW
	36.0 to 37.9	NORMAL
	35.9 or less	YELLOW
Heart rate (beats per minute)	120 or more	RED
	100 - 119	YELLOW
	50 - 99	NORMAL
	40 - 49	YELLOW
	39 or less	RED
Systolic blood pressure (mmHg)	160 or more	RED
	140 -159	YELLOW
	100 -139	NORMAL
	90 - 99	YELLOW
	89 or less	RED
Diastolic blood pressure (mmHg)	110 or more	RED
	90 -109	YELLOW
	40 - 89	NORMAL
	39 or less	RED
Urine output Hours since patient passed urine (tick box)	12 hours or less	NORMAL
	12 - 18 hours	YELLOW
	18 hours or more OR less than 0.5 ml/kg/hour	RED
Mental State (tick box)	Alert	NORMAL
	Not Alert	RED
Looks unwell (tick box)	No	NORMAL
	Yes	YELLOW
TOTAL YELLOW FLAGS		
TOTAL RED FLAGS		
ACTION TAKEN (IF REQUIRED) Yes (Y) / No (N)		

ACT NOW if patient triggers ONE RED or TWO YELLOW flags at any time. Escalate to clinical decision maker and start FAST-M decision tool.

UNITED AGAINST MATERNAL SEPSIS

THINK SEPSIS, ACT FAST-M

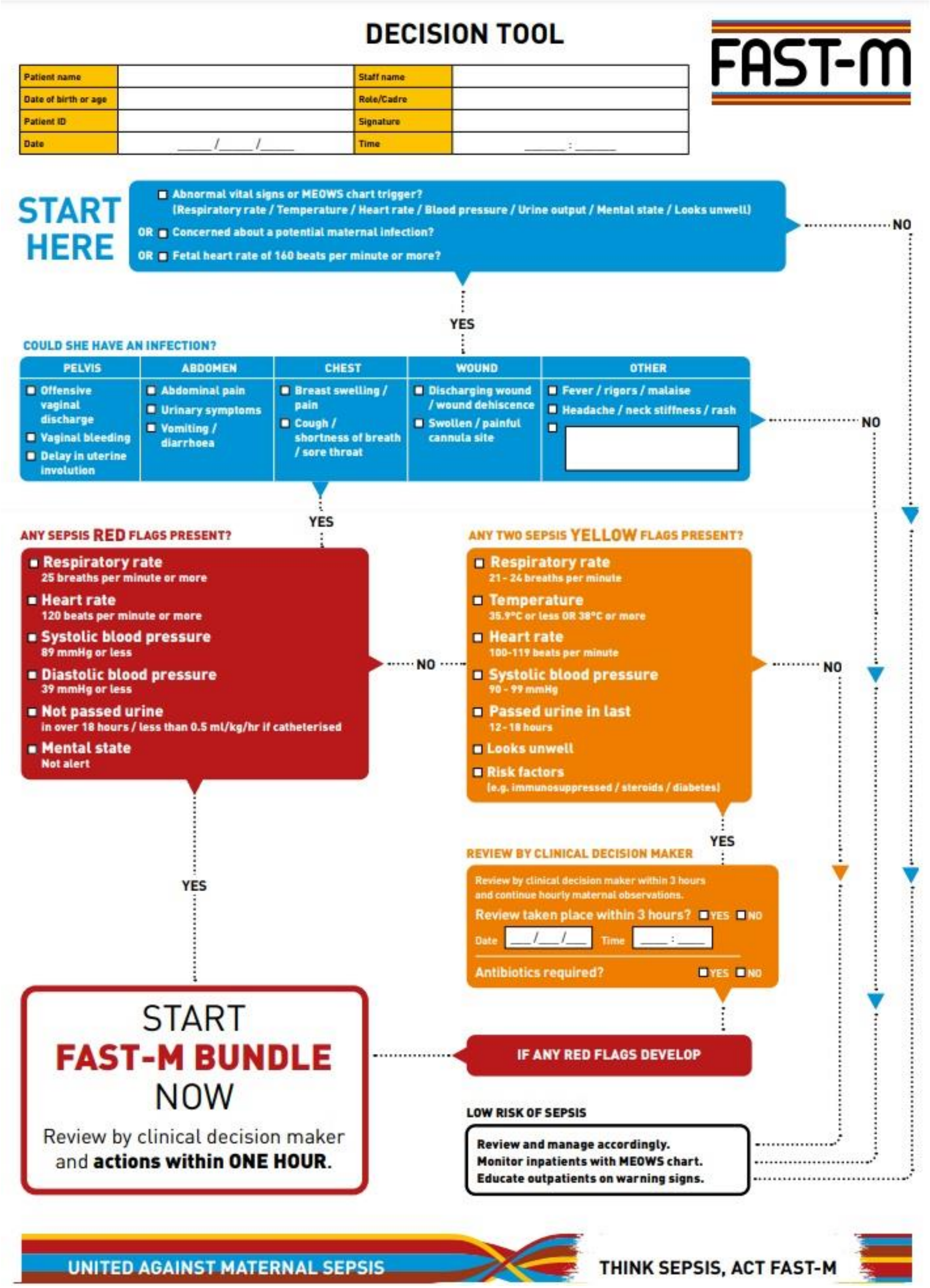
DECISION TOOL
FAST-M
Patient name
Date of birth or age
Patient ID
Date
____/____/____
Staff name
Role/Cadre
Signature
Time
______:______
START HERE
Abnormal vital signs or MEOWS chart trigger? (Respiratory rate / Temperature / Heart rate / Blood pressure / Urine output / Mental state / Looks unwell)
OR Concerned about a potential maternal infection?
OR Fetal heart rate of 160 beats per minute or more?
NO
YES
COULD SHE HAVE AN INFECTION?
PELVIS
Offensive vaginal discharge
Vaginal bleeding
Delay in uterine involution
ABDOMEN
Abdominal pain
Urinary symptoms
Vomiting / diarrhoea
CHEST
Breast swelling / pain
Cough / shortness of breath / sore throat
WOUND
Discharging wound / wound dehiscence
Swollen / painful cannula site
OTHER
Fever / rigors / malaise
Headache / neck stiffness / rash
NO
YES
ANY SEPSIS RED FLAGS PRESENT?
Respiratory rate
25 breaths per minute or more
Heart rate
120 beats per minute or more
Systolic blood pressure
89 mmHg or less
Diastolic blood pressure
39 mmHg or less
Not passed urine
in over 18 hours / less than 0.5 ml/kg/hr if catheterised
Mental state
Not alert
NO
ANY TWO SEPSIS YELLOW FLAGS PRESENT?
Respiratory rate
21 - 24 breaths per minute
Temperature
35.9°C or less OR 38°C or more
Heart rate
100-119 beats per minute
Systolic blood pressure
90 - 99 mmHg
Passed urine in last
12 - 18 hours
Looks unwell
Risk factors
(e.g. immunosuppressed / steroids / diabetes)
NO
YES
YES
REVIEW BY CLINICAL DECISION MAKER
Review by clinical decision maker within 3 hours and continue hourly maternal observations.
Review taken place within 3 hours? YES NO
Date __/__/__ Time ___:___
Antibiotics required? YES NO
START
FAST-M BUNDLE
NOW
Review by clinical decision maker and actions within ONE HOUR.
IF ANY RED FLAGS DEVELOP
LOW RISK OF SEPSIS
Review and manage accordingly.
Monitor inpatients with MEOWS chart.
Educate outpatients on warning signs.
UNITED AGAINST MATERNAL SEPSIS
THINK SEPSIS, ACT FAST-M

TREATMENT BUNDLE

FAST-M

REMEMBER TO COMPLETE THESE ACTIONS WITHIN ONE HOUR (1HR)

Patient name		Staff name	
D.O.B or age		Role/Cadre	
Patient ID		Signature	

Date & time of red flag observation	___/___/___ ___:___	Date & time bundle started	___/___/___ ___:___	Date & time of review by clinical decision maker	___/___/___ ___:___

F

FLUIDS (caution in pre-eclampsia, severe anaemia and pulmonary oedema)

Date	___/___/___	Time fluids initiated	___:___	Initials	
Details / reason not completed					

Give 500 ml crystalloid immediately. Repeat 500 ml boluses to a maximum of 30 ml/kg if hypotension persists.

A

ANTIBIOTICS

Date	___/___/___	Time started	___:___	Initials	
Details / reason not completed					

See antibiotic guidelines below

S

SOURCE – identify and treat the source of infection

Date	___/___/___	Time considered	___:___	Initials	
Details / reason not completed					

See source identification and treatment boxes below

T

TRANSPORT (to higher level hospital or location within hospital, if required)

Date & time transport considered	___/___/___ ___:___	Initials		Transport Required	☐ YES ☐ NO
Date & time transport requested	___/___/___ ___:___	Initials		☐ N/A	
Date & time patient left facility	___/___/___ ___:___	Initials			
Destination					
Reason for any delay					

M

MONITORING (start MEOWS chart if not already started. Repeat observations every 30 minutes until otherwise decided by clinical decision maker)

Date & time monitoring commenced	___/___/___ ___:___	Details / reason not completed	
Maternal / fetal monitoring should include	• Respiratory rate<br>• Temperature<br>• Heart rate<br>• Blood pressure	• Urine output<br>• Mental state<br>• Fetal heart rate	
Neonatal monitoring and review commenced	☐ YES ☐ NO ☐ N/A		

ANTIBIOTIC GUIDELINES

Insert local guidance here

Immediate treatment for Maternal Sepsis:

- Ceftriaxone 2 g IV once daily (if no IV access this can be given as 2 IM injections of 1 g in different sites).
- If possible intra-abdominal source add Metronidazole 500 mg IV three times daily or 400 mg PO three times daily.

If above antibiotic regime is not available then give:

- Chloramphenicol 1 g IV/IM four times daily plus Gentamycin 240 mg IV/IM once daily.

If maternal infection source is known, or as soon as it is identified:

- Use specific treatment based on Malawi Standard Treatment Guidelines.

IDENTIFY THE SOURCE

Consider

• Clinical history	• Blood cultures	• Sputum sample
• Clinical examination	• HIV and Malaria testing	• Imaging (abdominal, chest)
• Blood tests (if available)	• Urine sample	• Lumbar puncture
(FBC, U&Es, LFTs, CRP, clotting)	• Swabs (wound, vagina, throat)	• Other ____________

REMOVE / TREAT THE SOURCE

Consider

• Malaria treatment	• Removal of infected cannula / line
• Consider delivery of baby	• Hysterectomy
• Removal of retained products of conception	• Targeted antibiotics once source known
• Debridement of wound / drainage of collection	

UNITED AGAINST MATERNAL SEPSIS THINK SEPSIS, ACT FAST-M

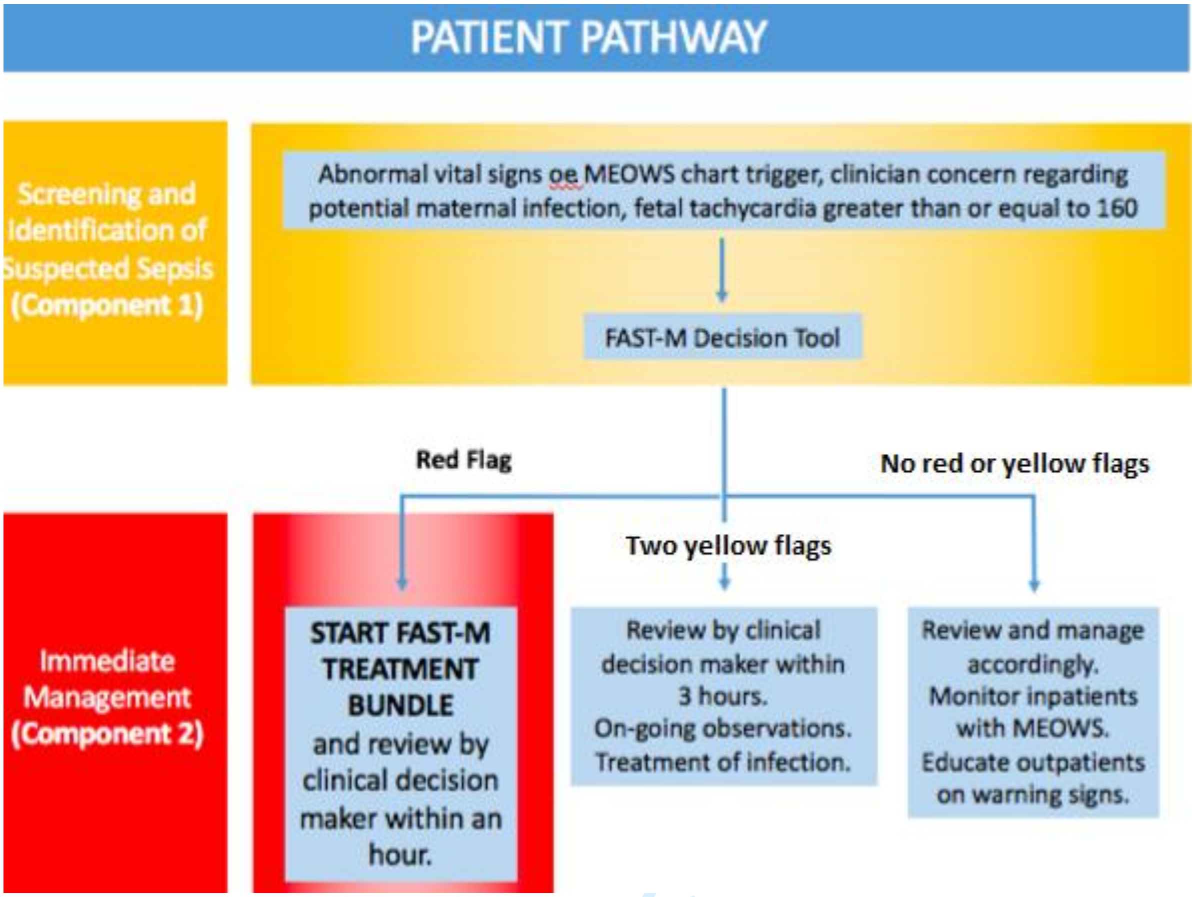
PATIENT PATHWAY
Screening and Identification of Suspected Sepsis (Component 1)
Abnormal vital signs oe MEOWS chart trigger, clinician concern regarding potential maternal infection, fetal tachycardia greater than or equal to 160
FAST-M Decision Tool
Red Flag
No red or yellow flags
Two yellow flags
Immediate Management (Component 2)
START FAST-M TREATMENT BUNDLE and review by clinical decision maker within an hour.
Review by clinical decision maker within 3 hours. On-going observations. Treatment of infection.
Review and manage accordingly. Monitor inpatients with MEOWS. Educate outpatients on warning signs.

view only

MODIFIED EARLY OBSTETRIC WARNING CHART (MEOWS CHART)

FAST-M

Contact clinical decision maker if patient triggers ONE RED or TWO YELLOW flags at any one time.

Patient		Patient ID		DOB/Age	

Date

Time

Initials

WRITE VALUES IN BOXES PROVIDED

Respiratory rate (breaths per minute)	25 or more	RED
	21 - 24	YELLOW
	11 - 20	NORMAL
	10 or less	RED
Oxygen saturations (%)	95 or more	NORMAL
	94 or less OR needing oxygen	RED
Temperature (°C)	38 or more	YELLOW
	36.0 to 37.9	NORMAL
	35.9 or less	YELLOW
Heart rate (beats per minute)	120 or more	RED
	100 - 119	YELLOW
	50 - 99	NORMAL
	40 - 49	YELLOW
	39 or less	RED
Systolic blood pressure (mmHg)	160 or more	RED
	140 -159	YELLOW
	100 -139	NORMAL
	90 - 99	YELLOW
	89 or less	RED
Diastolic blood pressure (mmHg)	110 or more	RED
	90 -109	YELLOW
	40 - 89	NORMAL
	39 or less	RED
Urine output Hours since patient passed urine (tick box)	12 hours or less	NORMAL
	12 - 18 hours	YELLOW
	18 hours or more OR less than 0.5 mL/kg/hour	RED
Mental State (tick box)	Alert	NORMAL
	Not Alert	RED
Looks unwell (tick box)	No	NORMAL
	Yes	YELLOW
TOTAL YELLOW FLAGS		
TOTAL RED FLAGS		
ACTION TAKEN (IF REQUIRED) Yes (Y) / No (N)		

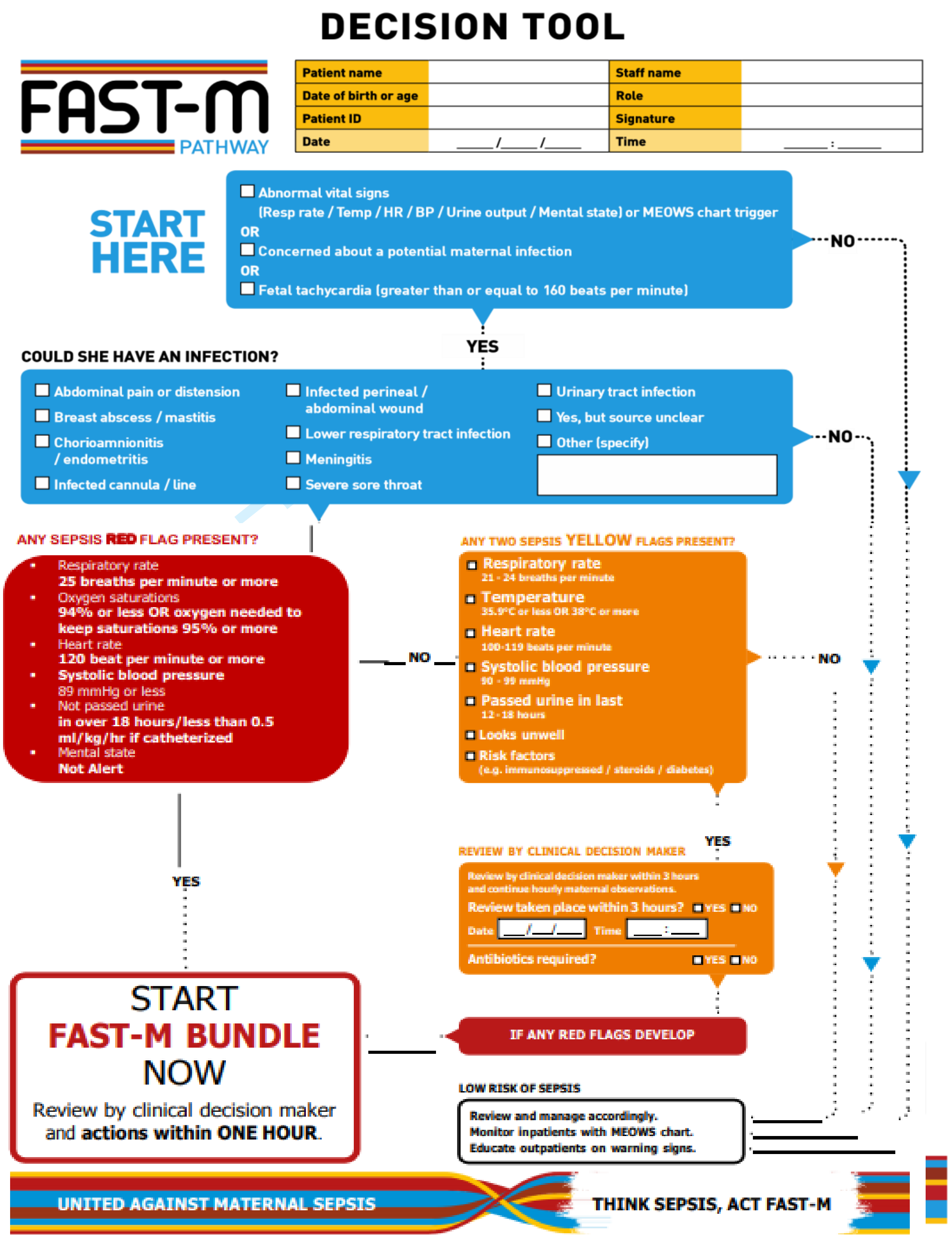
DECISION TOOL
FAST-M
PATHWAY
Patient name
Date of birth or age
Patient ID
Date ____/____/____
Staff name
Role
Signature
Time ____:____
START HERE
Abnormal vital signs
(Resp rate / Temp / HR / BP / Urine output / Mental state) or MEOWS chart trigger
OR
Concerned about a potential maternal infection
OR
Fetal tachycardia (greater than or equal to 160 beats per minute)
NO
YES
COULD SHE HAVE AN INFECTION?
Abdominal pain or distension
Breast abscess / mastitis
Chorioamnionitis / endometritis
Infected cannula / line
Infected perineal / abdominal wound
Lower respiratory tract infection
Meningitis
Severe sore throat
Urinary tract infection
Yes, but source unclear
Other (specify)
NO
ANY SEPSIS RED FLAG PRESENT?
Respiratory rate
25 breaths per minute or more
Oxygen saturations
94% or less OR oxygen needed to keep saturations 95% or more
Heart rate
120 beat per minute or more
Systolic blood pressure
89 mmHg or less
Not passed urine
in over 18 hours/less than 0.5 ml/kg/hr if catheterized
Mental state
Not Alert
NO
ANY TWO SEPSIS YELLOW FLAGS PRESENT?
Respiratory rate
21 - 24 breaths per minute
Temperature
35.9°C or less OR 38°C or more
Heart rate
100-119 beats per minute
Systolic blood pressure
90 - 99 mmHg
Passed urine in last
12 - 18 hours
Looks unwell
Risk factors
(e.g. immunosuppressed / steroids / diabetes)
NO
YES
REVIEW BY CLINICAL DECISION MAKER
Review by clinical decision maker within 3 hours and continue hourly maternal observations.
Review taken place within 3 hours? YES NO
Date ___/___/___ Time ____:____
Antibiotics required? YES NO
YES
START
FAST-M BUNDLE
NOW
Review by clinical decision maker and actions within ONE HOUR.
IF ANY RED FLAGS DEVELOP
LOW RISK OF SEPSIS
Review and manage accordingly.
Monitor inpatients with MEOWS chart.
Educate outpatients on warning signs.
UNITED AGAINST MATERNAL SEPSIS
THINK SEPSIS, ACT FAST-M

TREATMENT BUNDLE

FAST-M

REMEMBER TO COMPLETE THESE ACTIONS WITHIN ONE HOUR

Patient name		Staff name			
D.O.B or age		Role / Cadre			
Patient ID		Signature			
Date & time of red flag observation	__/__/__ __:__	Date & time bundle started	__/__/__ __:__	Date & time of review by clinical decision maker	__/__/__ __:__

**F**	**FLUIDS** (caution in pre-eclampsia, severe anaemia and pulmonary oedema)						
	Date	__/__/__	Time fluids initiated	__ : __	Initials		Give 500 ml crystalloid immediately. Repeat 500 ml boluses to a maximum of 30 ml/kg if hypotension persists.
	Details / reason not completed						
**A**	**ANTIBIOTICS**						
	Date	__/__/__	Time started	__ : __	Initials		See antibiotic guidelines below
	Details / reason not completed						
**S**	**SOURCE** – identify and treat the source of infection						
	Date	__/__/__	Time considered	__ : __	Initials		See source identification and treatment boxes below
	Details / reason not completed						

**T**	**TRANSPORT** (to higher level hospital or location within hospital, if required)				
	Date & time transport considered	__/__/__ __:__	Initials		Transport Required ☐ YES ☐ NO
	Date & time transport requested	__/__/__ __:__	Initials		☐ N/A
	Date & time patient left facility	__/__/__ __:__	Initials		
	Destination				
	Reason for any delay				

**M**	**MONITORING** (start MEOWS chart if not already started. Repeat observations every 30 minutes until otherwise decided by clinical decision maker)		
	Date & time monitoring commenced	__/__/__ __:__	Details / reason not completed
	Maternal / fetal monitoring should include	• Respiratory rate<br>• Oxygen Saturations<br>• Temperature<br>• Heart rate<br>• Blood pressure<br>• Urine output<br>• Mental state<br>• Fetal heart rate	
	Neonatal monitoring and review commenced	☐ YES ☐ NO ☐ N/A	

ANTIBIOTIC GUIDELINES

Insert local guidance here

Immediate treatment for Maternal Sepsis:

- Ceftriaxone 2 g IV once daily (if no IV access this can be given as 2 IM injections of 1 g in different sites).
- If possible intra-abdominal source add Metronidazole 500 mg IV three times daily or 400 mg PO three times daily.

If above antibiotic regime is not available then give:

- Tazobactam 4.5 g IV daily two time a day
- Meropenem 1 g IV daily two times a day

IDENTIFY THE SOURCE

Consider

• Clinical history	• Blood cultures	• Sputum sample
• Clinical examination	• HIV and Malaria testing	• Imaging (abdominal, chest)
• Blood tests (if available) (FBC, U&Es, LFTs, CRP, clotting)	• Urine sample	• Lumbar puncture
	• Swabs (wound, vagina, throat)	• Other ____________

REMOVE / TREAT THE SOURCE

Consider

• Malaria treatment	• Removal of infected cannula / line
• Consider delivery of baby	• Hysterectomy
• Removal of retained products of conception	• Targeted antibiotics once source known
• Debridement of wound / drainage of collection	

UNITED AGAINST MATERNAL SEPSIS THINK SEPSIS, ACT FAST-M

Paper Title:

Evaluation of the feasibility of the FAST-M maternal sepsis intervention in Pakistan, a protocol

Author Names:

Sheikh Irfan Ahmed[1]

Raheel Sikandar[2]

Rubina Barolia[1]

Bakhtawar M. Hanif Khowaja[1]

Kashif Ali Memon [2]

James Cheshire[3]

Catherine Dunlop [3]

Arri Coomarasamy[3]

Lumaan Sheikh[1]

David Lissauer[4, 5]

Affiliations & full institutional mailing addresses of all authors

[1] Aga Khan University Hospital, National Stadium Road, Karachi city, Pakistan. P.O. Box 3500 Postal code: 74800

[2] LUMHS Hospital Liaquat University of Health and Medical Sciences, Hyderabad city, Pakistan. Postal Code: 76090

[3] Institute of Metabolism and Systems Research, University of Birmingham, Edgbaston, Birmingham, UK, B15 2TT.

[4] Institute of Life Course and Medical Sciences, University of Liverpool, Liverpool, William Henry Duncan Building, Liverpool, UK, L7 8TX.

[5] Malawi-Liverpool-Wellcome Trust Clinical Research Programme, Chichiri, Blantyre 3, Malawi

For peer review only - http://bmjopen.bmj.com/site/about/guidelines.xhtml

Email addresses

sheikh.irfan@aku.edu

raheel.sikandar@lumhs.edu.pk

rubina.barolia@aku.edu

bakhtawar.hanif@aku.edu

drkashifmemon@yahoo.com

james.cheshire@nhs.net

catherinedunlop@nhs.net

A.Coomarasamy@bham.ac.uk

Lumaan.sheikh@aku.edu

David.Lissauer@liverpool.ac.uk

Corresponding Author*:

Sheikh Irfan Ahmed

For peer review only - http://bmjopen.bmj.com/site/about/guidelines.xhtml

Abstract

Background: Maternal sepsis is a life-threatening condition, defined by organ dysfunction caused by infection during pregnancy, childbirth and the postpartum period. It is estimated to account for between one tenth and half of all maternal deaths globally. An international stake-holder group, including the World Health Organization, developed a maternal sepsis management bundle called "FAST-M" for resource limited settings through a synthesis of evidence and international consensus. The FAST-M treatment bundle consists of five components: **F**luids, **A**ntibiotics, **S**ource identification and control, assessment of the need to **T**ransport or Transfer to a higher level of care and ongoing **M**onitoring (of the mother and neonate). This study aims to adapt the FAST-M intervention and evaluate its feasibility in Pakistan.

Methods: The proposed study is a mixed method, with a before and after design. The study will be conducted in two phases at Liaquat University of Medical and Health Sciences, Hyderabad. In the first phase, we will adapt the bundle care tools for the local context and assess in what circumstances different components of the intervention are likely to be effective, by conducting interviews and a focus group discussion (the Adaptation Phase). In the second phase, we will evaluate the feasibility of the FAST-M intervention (the Feasibility Assessment Phase).

Discussion: The utilisation of bundles can facilitate recognition and timely management of maternal sepsis. There is a need to adapt, integrate and optimise a bundled care approach in low-resource settings in Pakistan to minimise the burden of maternal morbidities and mortalities due to sepsis.

Keywords: FAST-M intervention, maternal sepsis, Pakistan, qualitative study, sepsis bundle, care bundle, complex intervention, low-resource setting, feasibility study, maternal deaths.

For peer review only - http://bmjopen.bmj.com/site/about/guidelines.xhtml

Background

Pregnancy and childbirth-related complications are a major public health concern [1]. Every day approximately 830 women die from preventable causes related to pregnancy and childbirth and almost one-third of these occur in South Asia [2]. Physiological and immunological variations during pregnancy and the postpartum period predispose women to risks of these complications [3]. About 60% of maternal deaths occur during delivery and postpartum period [4]. Most of the maternal deaths occur within 24 to 72 hours of delivery where postpartum hemorrhage, eclampsia and maternal sepsis are the leading causes of maternal mortality [5].

The World Health Organization estimates suggests that globally, maternal sepsis accounts for about one tenth of the maternal deaths around the time of childbirth and is the third most common cause of maternal mortality [7]. Whilst the maternal mortality related to sepsis has decreased considerably in high income countries accounting for 2.1% of the total maternal deaths, the numbers are still high in the lower income countries accounting for up to 15.1% of maternal deaths annually [8]. However, more recent WHO estimates that were focused specifically on understanding better the contribution of maternal infection to adverse outcomes suggested that up to half of all maternal deaths were actually infection related [9]. A substantial proportion of the improvements in maternal outcomes in high income countries was attributed to the prevention and appropriate treatment of maternal sepsis [10].

Early warning scores, modules of educational material in routine healthcare settings and the bundled approach to sepsis management in high income countries have been effective in reducing maternal mortalities and morbidities [10]. A more rapid completion of a 3-hour bundle of sepsis care and rapid administration of antibiotics were found to be associated with lower risk-

For peer review only - http://bmjopen.bmj.com/site/about/guidelines.xhtml

adjusted in-hospital mortality (p <0.001) [11]. Despite the improvement of sepsis care in high income countries, there is still lack of maternal sepsis-care bundle specific to the maternal population of low-resource settings [12].

The development of a maternal sepsis treatment bundle has been identified as an international "Priority Action" [13]. In collaboration with the WHO Maternal Sepsis Initiative, a Delphi approach was adopted to select contributory components to a maternal sepsis treatment bundle in low-resource settings [14]. The components selected were: **F**luids, **A**ntibiotics, **S**ource identification and control, assessment of the need to **T**ransport/Transfer to a higher level of care and ongoing **M**onitoring (of the mother and neonate). The treatment bundle was named "FAST-M" as a memorable acronym for both communication and awareness-raising [14].

The FAST-M intervention was implemented in districts of Malawi to evaluate the feasibility of early identification and management of maternal sepsis, and demonstrated significant improvements in maternal sepsis care [15]. The components included a 1) Maternal Early Obstetric Warning System (MEOWS) chart and FAST-M decision tool, 2) FAST-M treatment bundle and 3) The FAST-M implementation programme which consisted of the following: training programme, sepsis champions, task shifting, performance dashboards and data feedback to promote systems level change [15].

The FAST-M intervention has the capacity to strengthen maternal sepsis care as demonstrated in Malawi. We therefore aim to evaluate implementation of the FAST-M intervention to assess improvement in maternal sepsis care in low-resource setting of Pakistan.

For peer review only

Study Aim

This study aims to determine whether it is feasible to introduce a complex intervention (including a bundled approach) for maternal sepsis care in low resource setting of Pakistan; and to describe the facilitators and barriers to its implementation.

Study Objectives

- To adapt FAST-M bundle care tools (MEOWS chart, decision tool and treatment bundle) to the context in Pakistan
- We will also investigate how to optimally implement the approach in Pakistan's low resource hospital
- To understand the barriers and facilitators to these approaches in these settings
- Assess whether the use of the FAST-M intervention is feasible in the local healthcare system and improves sepsis care.
- Prepare the FAST-M intervention for a large-scale intervention trial.

Methods

Study setting

The study will be conducted at Liaquat University of Medical Health Sciences (LUMHS), which is a public sector tertiary hospital located in Hyderabad district of Pakistan. The hospital has a total of 3000 beds and 35 departments which serves a large number of mostly underprivileged populations. The hospital provides 24 hours' emergency cover to patients coming from nearby urban and rural areas. LUMHS has three Obstetrics and Gynecology units.

For peer review only - http://bmjopen.bmj.com/site/about/guidelines.xhtml

The current data from the facility shows that a total of approximately 11205 patients were admitted in OBGYN units from the period of January to August 2021; and the maternal mortality rate was recorded as 159/11205 (1.4%). Out of these 159 deaths, 45 were due to confirmed maternal sepsis (28.3%). These indicators direct that there is a need of a robust system to early detect and manage maternal sepsis cases in the hospital.

Study design

The study will use a mix-method design and will be conducted in two phases.

Phase 1-Adaptation of FAST-M intervention (Qualitative)

For a FAST-M bundle to be effective in Pakistan, it is necessary to identify how best to implement the FAST-M bundle in the context of local settings. In order to adapt this intervention, a systematic method will be taken to understand the nature of existing practices and an appropriate system for characterising the intervention and its components that can make use of this understanding. This constitute phase 1 of the study.

This formative research (phase 1) will adopt a qualitative research design involving focus group discussion (FGD) and key-informant interviews (KIIs) and a purposive sampling approach. The aim of group discussion and interviews will be to engage health practitioners, government officials and other key stakeholders to understand the behavior of existing practices in the study setting for maternal sepsis care, to finalize the FAST-M tools for the context of Pakistan, and to identify various facilitators and barriers that may influence implementation of the FAST-M intervention. The FGD and KIIs will be conducted using interview guides developed through the use of the Consolidated Framework for Implementation Research (CFIR) [16].

For peer review only - http://bmjopen.bmj.com/site/about/guidelines.xhtml

Consolidated Framework for Implementation Research (CFIR)

The CFIR is a commonly used framework to facilitate implementation research design, evaluate and implement evidence-based interventions, and comprises five major domains: 1) Intervention characteristics, 2) Outer setting, 3) Inner setting, 4) Characteristics of individuals, and 5) Process of implementation. It is categorized as a determinant framework with the objective to understand and explain factors (individual or organization) which influence implementation outcomes [16]. CFIR has been used in a wide range of studies because this flexible framework can be tailored to different settings across multiple contexts [17]. We aim to use the tailored CFIR framework to assess critical barriers and facilitators to implementation that need to be addressed at multiple levels if the FAST-M bundle is to be successfully optimized, and adopted in health care practices in Pakistan (Appendix-1).

The interview guides (Appendix-2) for KIIs and the FGD have been developed using five major domains of CFIR to identify existing practices for sepsis management. These guides will also identify the facilitators and barriers to implementation of FAST-M intervention in the study setting. The identification of existing practices for maternal sepsis care and facilitators and barriers in phase 1 will then form the basis of feasibility testing of FAST-M intervention in phase 2.

Inclusion criteria for KIIs and the FGD

- HCPs including physicians, nursing staff, healthcare administrators who are associated with maternal sepsis care and management
- HCPs who have worked at the study site for last six months

Sample size

15 to 20 semi-structured key informant interviews are planned in the qualitative phase of the study until data saturation is reached. One focus group will be conducted before initiation of the study to adapt the tools and identify implementation approaches; and a second will be conducted at the end of the study as a summative evaluation of the study to identify perceptions about success of implementation. Therefore, two focus group discussions (before and after implementation) will be conducted with 8-10 health care providers in each discussion.

Data collection and management

A semi-structured interview guide has been developed to explore healthcare professionals' views and attitudes towards FAST-M intervention and its implementation at their facility. Before beginning the interview, the qualitative researchers will describe the FAST-M bundle components and the patient referral pathway demonstrating the algorithm and summary for utilization of FAST-M bundle care tools (Appendix-3).

A free flow of discussion among participants will be encouraged, using probes from these discussions to obtain healthcare professionals' perceptions about the feasibility of the FAST-M intervention. Interviews will be conducted face-to-face in Urdu and English according to the participants' preference, and will be audio recorded following consent from study participants. Interviews and focus group discussion will be conducted by experienced study team members who are also trained qualitative researchers. Detailed field notes will be also taken during each interview to capture non-verbal language and cues.

All data will be kept confidential for seven years on password-protected computers and/or locked filing cabinets only accessible to members of the research team. During transcription, audio-

For peer review only - http://bmjopen.bmj.com/site/about/guidelines.xhtml

recordings will be referenced only with an identification number for anonymity of participants, with all identifying information removed before using the software analysis tool.

COVID-19- Standard Operating Procedures (SOPs)

In view of current of current COVID-19 pandemic situation, all project related activities will comply with standard operating procedures (SOPs). The following measures will be taken related to this study: 1) All research staff will be provided with appropriate masks, sanitizers, and/or other applicable Personal protective equipment (PPE) to the field staff; 2) Daily mandatory screening for COVID-19 symptoms of all project staff; 3) KIIs and FGDs will be conducted with social distancing (6 feet) with all vaccinated participants wearing face coverings.

Analysis plan

Qualitative data gained through individual interviews and FGDs will be audio recorded, transcribed and analysed using an inductive approach to determine the facilitators and barriers for implementation of the intervention and will be summarized according to CFIR domains. This will help to understand the important contextual features that are helping or hindering the operationalization of the FAST-M intervention.

The analysis will be an ongoing iterative process during phase 1 of this study. The research team will conduct multiple reviews of the transcripts and tapes to familiarize themselves with the data and identify initial themes that will be reflexive and interactive. Analysis will begin as soon as the first interview is completed in phase 1 and will be continued concurrently with data collection to help determine when new information is no longer being generated from interviews.

Although, we identified the CFIR as the appropriate framework, additional codes may emerge during the familiarization process to develop a thematic framework from experiences of

For peer review only - http://bmjopen.bmj.com/site/about/guidelines.xhtml

participants. The codes, categories and themes will be developed using NVivo version 10 (QSR International, Pty Ltd) software.

An audit trail will be used to document our decision-making process. Sections of the transcripts will be charted, organized by CFIR domains, and then re-framed to better reflect descriptions from participants. The primary team will review the codes and associated themes multiple times to check for potential biases, to ensure they are reflecting participants' words and meanings, and improve the credibility of their interpretation of the interviews. Initial findings will be shared with a group of participants to help with interpretation and generate meaning from the data.

The facilitators and hindering factors will be identified through phase 1 of the work. The FAST-M bundle care tools (MEOWS chart, decision tool and treatment bundle) will be modified through construal gained from interviews and discussion with health care providers.

Phase 2- Intervention phase

Following phase 1, intervention phase will be implemented for the feasibility testing.

Study population

During the intervention phase, patients will be assessed by a healthcare practitioner on decision to initiate screening for potential maternal sepsis that will be based on the following inclusion criteria:

- Women who are pregnant or within 6 weeks of miscarriage, termination of pregnancy or delivery
- Abnormal maternal observations triggered on the inpatient MEOWS chart
- Healthcare practitioner concern regarding potential maternal infection

For peer review only - http://bmjopen.bmj.com/site/about/guidelines.xhtml

- Fetal tachycardia greater than or equal to160 beats per minute

Sample size

For enrollment of sepsis cases, we will power to a primary process outcome of "sepsis management compliance”. This is defined as "the proportion of patients admitted with features of sepsis who receive appropriate monitoring (full set of vital sign measurements on admission) and antibiotics within 1 hour (if required)." This means the notes of all patients with suspected or confirmed sepsis will be reviewed and their data would be collected using study Case Report Forms (CRFs).

Assuming baseline compliance is less than 10%, grounded on observations from FAST-M study in Malawi, to detect an increase in compliance to 20%, with an alpha of 0.05, we will require the observation of 199 participants in each phase to achieve a power of 80%. This is adequate precision to allow important increases to be estimated. Allowing for loss to follow-up and missing / laboratory results, we consider an initial sample size of 400 as appropriate to allow the study to have adequate power to detect an increase in compliance. This number of cases will be feasible to collect within 6 months, based on current rate of sepsis from hospital records of anticipated site. The flow of participants through the study is presented in Appendix-4.

Study period

This feasibility study is anticipated to run for seven months. This includes a baseline assessment period of two months, and training programme planned to schedule at completion of baseline phase before commencing intervention phase of four months.

The intervention phase will be introduced after training all health care provides involved in management of maternal sepsis at the study site. At the start of the intervention phase, FAST-M

For peer review only - http://bmjopen.bmj.com/site/about/guidelines.xhtml

bundle care tools will be introduced including MEOWS chart, FAST-M decision tool, and FAST-M treatment bundle. Appendix -5 provides the summary of enrollment, intervention and assessment

Modified early obstetric warning score

MEOWS stands for modified early obstetric warning score (MEOWS) to identify suspected maternal sepsis patients. This tool helps in identifying any early warning scores used to track the physiological parameters of an individual over time onto a chart, with guidance thresholds to trigger clinical action of they become abnormal [18]. The MEOWS chart used during implementation of the FAST-M intervention in the districts of Malawi will be adapted in context of Pakistan for the purpose of this feasibility study [15].

The use of obstetric early warning systems (OEWS) in UK maternity units was recommended in the 2007 Confidential Enquiry into Maternal and Child Health (CEMACH) report as an adjunct to reducing maternal morbidity and mortality. [19] MEOWS consisted of scores of respiratory rate, oxygen saturation, temperature, heart rate, blood pressure, assessment of urine, including for proteinuria, color of amniotic fluid, neurological response, pain score, assessment of lochia, and an overall assessment of whether the woman appears well [19]. Clinical action is triggered by a single parameter exceeding a red threshold or any two parameters exceeding a yellow threshold. MEOWs chart have been widely adopted in the UK and internationally [20].

To complete the MEOWS chart, the healthcare providers involved in the study will be trained to record patient observations (heart rate, respiratory rate, blood pressure, conscious level, urine output and temperature) and fetal heart rate (if applicable) from medical records. These observations will be charted on a MEOWS chart in the inpatient setting.

For peer review only - http://bmjopen.bmj.com/site/about/guidelines.xhtml

Decision tool

Abnormal observations (indicated by a single red or two yellow thresholds) will trigger a review by an attending doctor or nurse. This will be agreed locally prior to study commencement. These patients will then be screened for potential sepsis using the FAST-M decision tool. In addition to abnormal maternal observations, cases of suspected sepsis will also be identified using the FAST-M patient pathway when prompted by attending clinician concern regarding potential maternal sepsis or an increased fetal heart rate greater than or equal to160 beats per minute.

Patients will be defined as having or are at a higher risk of having sepsis, who will trigger a red flag on the decision tool and will be commenced immediately on the FAST-M treatment bundle pathway. These patients will receive a review from a doctor/nurse as soon as possible, with the bundle initiated within one hour. Those patients who trigger two yellow flags on the decision tool and have or at a higher risk of having sepsis require a review from a doctor/nurse within three hours. All suspected cases will remain in observation for possible development of red flags. Half-hourly (if red trigger) or hourly (if two yellow triggers) observations will be made in the first instance, until otherwise specified by an attending clinical decision maker. Those patients without at least one red or two yellow flags will be considered to have a low risk of sepsis and will be managed according to local guidelines by the screening healthcare practitioner.

For peer review only - http://bmjopen.bmj.com/site/about/guidelines.xhtml

FAST-M treatment bundle

Patients managed with the FAST-M treatment bundle will have their treatment recorded on the FAST-M treatment bundle form including documentation of actions completed and any reasons for not completing certain component of the bundle.

The FAST-M treatment bundle consists of the timely consideration of all the following:

- Fluids
- Antibiotics
- Source identification and control
- Assessment of the need to Transport / Transfer to a high level of care
- Ongoing Monitoring (of the mother and neonate)

Co-interventions for implementation of intervention

Training Programme

Multiple full day training sessions by the study team will be delivered to healthcare practitioners working for maternal care and sepsis management at the study site. The interactive sessions will be offered in English and Urdu languages for each healthcare practitioner to understand the processes completely. Any requirement for supplementary educational material such as posters and a study booklet will be determined during the implementation programme via feedback from front line clinical staff and stakeholders on facilitators and barriers to use of the tools. This will be done using qualitative interviews and focus groups discussion.

For peer review only - http://bmjopen.bmj.com/site/about/guidelines.xhtml

The training and implementation programme is likely to consist of:

• Background information on maternal sepsis, including risk factors, signs and symptoms and the potential consequences if untreated

• Use of the MEOWS chart to track and trigger the recognition of deteriorating patients

• Use of the FAST-M decision tool to recognise and screen for potential study participants at risk of maternal sepsis

• Use of the FAST-M treatment tool to initiate the bundle components

• Guidance around implementing the individual components of the FAST-M bundle

• Use of feedback tools (run chart and dashboard) and approaches the team can use to work together to improve compliance and outcomes

Post training, an impact survey will be made to measure the extent to which skills and knowledge learned in the program have translated into improved behavior among participants who attended the training program.

Clinical champions

The local clinical champions and team leaders will be identified and trained to take a lead at study sites from different units where study will be implemented, and will remain engaged throughout the implementation process. The overarching goal of each champion will be to encourage engagement and compliance with the FAST-M bundle. To achieve this goal, champions at each site will be engaged in a number of key activities: disseminating knowledge, advocating, navigating boundaries, facilitating consensus, arranging meetings with stakeholders,

For peer review only - http://bmjopen.bmj.com/site/about/guidelines.xhtml

tracking quality indicators and developing organizational communication strategies and relationships.

Ongoing improvement approaches

Ongoing improvement practices at different units of the study site will be carried out by clinical champions of the respective units. The improvement strategies include: 1) weekly/biweekly training of health care providers on FAST-M tools, 2) display of run charts, dashboards in units to demonstrate rate of maternal sepsis and outcomes of maternal sepsis cases over-time, and 3) meeting with stakeholders for communicating needs and requirements for implementation of the FAST-M intervention. Appendix-6 shows the summary of ongoing improvement approaches planned to implement for FAST-M implementation

An overview of the implementation of the complex intervention is illustrated in the figure below;

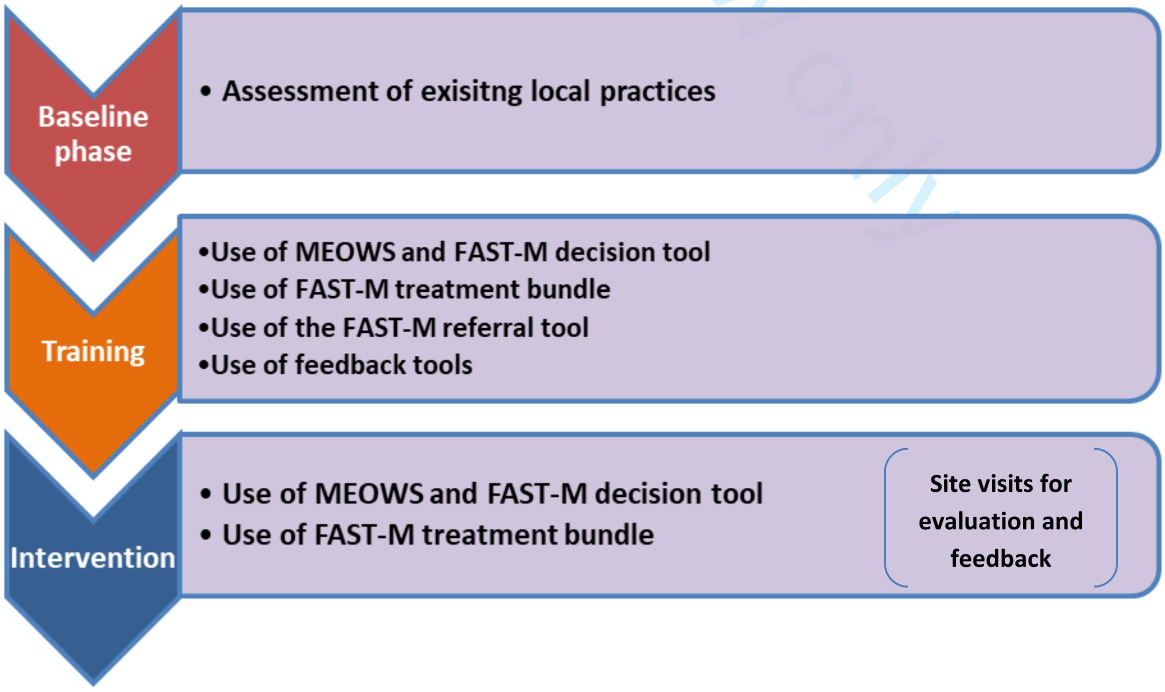

Data collection and management

During the intervention phase, data will be collected by a member of the research team who will not be part of the clinical team. Data will be collected using CRFs on various outcomes; structural, clinical, organizational and any adverse events.

If the patient requires a transfer as part of the FAST-M treatment bundle to any other health facility due to shortage of beds or other resources, the data collector will continue to follow up the patient's clinical outcomes. The data collection team will keep their study site updated on their performance using this data, and will visually display it on run charts and dashboards and work on strategies to improve performance. The data will be maintained in an investigator file to be secured in a locked cabinet. Information recorded on the data collection sheet will be recorded in a database located on a secure server.

Analysis plan

Quantitative analyses will be done to assess numerous outcomes; process, organizational, clinical, structural and adverse events with quantitative comparisons made between before and after implementation of the bundle. Quantitative data will be analysed using percentages, means, medians interquartile ranges and 95% confidence intervals and the change identified over time. Binary outcomes will be analysed using logistic regression and continuous measures by linear regression.

A mixed methods approach will be used to explore the implementation of the intervention. In this approach both quantitative and qualitative data collection methods will be used, and then integrated to draw conclusions. A sequential exploratory design will be used to collect qualitative

For peer review only - http://bmjopen.bmj.com/site/about/guidelines.xhtml

data for adaption of the FAST-M bundle care tools and will be applied to make these tools contextual based. This will be then followed by the implementation of contextual based modified FAST-M tools at the study setting. This mixed-methods study will help in exploring the perspectives and adaptation of FAST-M intervention in phase 1 and evidence of its feasibility in phase 2 of the study. This will allow us to assess practicality of implementation in order to build a robust and successful full-scale trial for future.

Main outcome measurements

We will explore a range of outcomes measurement for maternal sepsis care. Primary process include 1) the proportion of patients admitted with features of sepsis who received appropriate monitoring (full set of vital sign measurements on admission recorded on MEOWS chart) 2) the proportion of women with suspected maternal sepsis received antibiotics within 1 hour (if required), 3) the proportion of women with suspected maternal sepsis receiving the FAST-M treatment bundle (including each bundle component) within 1 hour of identification of sepsis. Secondary outcomes will include: 1) the proportion of women with suspected maternal sepsis referred to clinical decision maker on the basis of abnormal vital signs records; and 2) the proportion of women with suspected maternal sepsis receiving a clinical review by a senior clinical decision maker following their diagnosis.

Potential Harms

Fluid resuscitation in patients with sepsis if not managed appropriately can precipitate volume overload and subsequent pulmonary edema. This is a particular concern in patients with preeclampsia. Clear teaching and guidance regarding fluid resuscitation will be provided during the training programme. When fluid resuscitating patients with suspected maternal sepsis, the

For peer review only - http://bmjopen.bmj.com/site/about/guidelines.xhtml

decision regarding the rate of fluid administration will be made by the responsible clinician based on clinical examination findings and ongoing monitoring.

Discussion

Overall, bundle care tools have the potential to enhance improvements in sepsis care [11]. However, the implementation challenges posed by these bundles should be examined, especially in low-resource settings.

The FAST-M maternal sepsis intervention has the potential to be used as an integrated strategy for early recognition and management of maternal sepsis in low resource health settings.

This mixed-method study will establish whether it is feasible to implement the FAST-M bundle for early identification and management of maternal sepsis in Pakistan. A large multi-country interventional trial is anticipated to ascertain the effectiveness of the bundle to improve maternal sepsis care and outcomes in low and middle income countries. The long-term vision is that the intervention will then be trialled in other settings across Pakistan. The study findings will be disseminated to clinicians and key stakeholders to formulate appropriate bundle care tools for sepsis care. This will help reduce the high rate of maternal mortalities caused by sepsis.

Abbreviations

CFIR: Consolidated Framework for Implementation Research; FAST-M: Fluids, Antibiotics, Source control, assessment of the need to Transport/Transfer to a higher level of care and ongoing Monitoring (of the mother and neonate);FGD: Focus Group Discussion ; HCPs: Health Care Providers; KIIs: Key Informant Interviews; LMIC: Low Middle Income Countries;

For peer review only - http://bmjopen.bmj.com/site/about/guidelines.xhtml

LUMHS: Liaquat University of Medical Health Sciences; MEOWS: Maternal Early Obstetric Warning Signs; SSC: Surviving Sepsis Campaign

Declarations

Ethics approval and consent to participate

Ethical approval for this study was obtained from the LUMHS hospital [REC/-886, 4-87], Aga Khan University Ethical Review Committee [2019-2061-7102] and National Bioethics Committee [515/20/]. Participants will be asked to provide written consent to indicate their willingness to participate. Voluntary participation and the right to ask any questions and to decline participation at any time will be emphasized during the data collection.

Consent for publication

Written consent for publication will be obtained from all study participants.

Availability of data and materials

All data developed for this intervention is available from the corresponding author on reasonable request.

Competing interests

The authors declare that they have no competing interests.

Funding

For peer review only - http://bmjopen.bmj.com/site/about/guidelines.xhtml

This project is funded by the University of Birmingham, University of Liverpool, National Institute of Health Research and Bill and Melinda Gates Foundation.

Authors' contributions

SI, DL, RB & LS conceptualized the design of the study and creation of data collection tools. DL, AC, RB, JS, CD provided feedback on the first draft. SI & BK edited and wrote the final draft. The authors read and approved the final manuscript.

Acknowledgements

We would like to acknowledge Dr. Anna Blennerhassett, Dr. Fahmida Parveen and Dr. Sehrish Khan for their expert contributions to the project.

For peer review only

For peer review only - http://bmjopen.bmj.com/site/about/guidelines.xhtml

References

1. World Health Organization. Managing complications in pregnancy and childbirth: a guide for midwives and doctors. World Health Organization; 2017.
2. Roser M, Ritchie H. Maternal mortality. Our World in Data. 2013 Nov 12.
3. Neuman H, Koren O. The pregnancy microbiome. Intestinal microbiome: functional aspects in health and disease. 2017;88:1-0.
4. Hirshberg A, Srinivas SK. Epidemiology of maternal morbidity and mortality. InSeminars in perinatology 2017 Oct 1 (Vol. 41, No. 6, pp. 332-337). WB Saunders.
5. World Health Organization, Unicef. Trends in maternal mortality: 1990 to 2010: WHO, UNICEF, UNFPA and The World Bank estimates.
6. Bonet M, Souza JP, Abalos E, Fawole B, Knight M, Kouanda S, Lumbiganon P, Nabhan A, Nadisauskiene R, Brizuela V, Gülmezoglu AM. The global maternal sepsis study and awareness campaign (GLOSS): study protocol. Reproductive health. 2018 Dec;15(1):1-7.
7. Cebekhulu S, Cornelissen L, Pattinson RC. Too little, too late: the recurrent theme in maternal deaths due to sepsis.
8. Zahr CA, Wardlaw TM, Choi Y. Maternal mortality in 2000: estimates developed by WHO, UNICEF and UNFPA. World Health Organization; 2004.
9. Bonet M, Brizuela V, Abalos E, Cuesta C, Baguiya A, Chamillard M, Fawole B, Knight M, Kouanda S, Lumbiganon P, Nabhan A. Frequency and management of maternal infection in health facilities in 52 countries (GLOSS): a 1-week inception cohort study. The Lancet Global Health. 2020 May 1;8(5):e661-71.

For peer review only - http://bmjopen.bmj.com/site/about/guidelines.xhtml

10. Rudd KE, Kissoon N, Limmathurotsakul D, Bory S, Mutahunga B, Seymour CW, Angus DC, West TE. The global burden of sepsis: barriers and potential solutions. Critical Care. 2018 Dec;22(1):1-1.

11. Seymour CW, Gesten F, Prescott HC, Friedrich ME, Iwashyna TJ, Phillips GS, Lemeshow S, Osborn T, Terry KM, Levy MM. Time to treatment and mortality during mandated emergency care for sepsis. New England Journal of Medicine. 2017 Jun 8;376(23):2235-44.

12. Vasco M, Pandya S, Van Dyk D, Bishop DG, Wise R, Dyer RA. Maternal critical care in resource-limited settings. Narrative review. International journal of obstetric anesthesia. 2019 Feb 1;37:86-95.

13. Bonet M, Souza JP, Abalos E, Fawole B, Knight M, Kouanda S, Lumbiganon P, Nabhan A, Nadisauskiene R, Brizuela V, Gülmezoglu AM. The global maternal sepsis study and awareness campaign (GLOSS): study protocol. Reproductive health. 2018 Dec;15(1):1-7.

14. Lissauer D, Cheshire J, Dunlop C, Taki F, Wilson A, Smith JM, Daniels R, Kissoon N, Malata A, Chirwa T, Lwesha VM. Development of the FAST-M maternal sepsis bundle for use in low-resource settings: a modified Delphi process. BJOG: An International Journal of Obstetrics & Gynaecology. 2020 Feb;127(3):416-23.

15. Cheshire J, Jones L, Munthali L, Kamphinga C, Liyaya H, Phiri T, Parry-Smith W, Dunlop C, Makwenda C, Devall AJ, Tobias A. The FAST-M complex intervention for the detection and management of maternal sepsis in low-resource settings: a multi-site evaluation. BJOG: An International Journal of Obstetrics & Gynaecology. 2021 Feb 4.

16. Damschroder LJ, Aron DC, Keith RE, Kirsh SR, Alexander JA, Lowery JC. Fostering implementation of health services research findings into practice: a consolidated

For peer review only - http://bmjopen.bmj.com/site/about/guidelines.xhtml

framework for advancing implementation science. Implement Sci [Internet]. 2009 Aug 07 [cited 2018 Jan 23]; 4(50). Available from: https://implementationscience.biomedcentral.com/articles/10.1186/17485908-4-50.

17. Kirk MA, Kelley C, Yankey N, Birken SA, Abadie B, Damschroder L. A systematic review of the use of the consolidated framework for implementation research. Implement Sci [Internet]. 2016 May [cited 2018 Jan 18]; 11(72)

18. Royal College of Physicians. National Early Warning Score (News): Standardising the Assessment of Acute-Illness Severity in the NHS. London, UK: RCP; 2012.

19. Mhyre JM, D'Oria R, Hameed AB, Lappen JR, Holley SL, Hunter SK, Jones RL, King JC, D'Alton ME. The maternal early warning criteria: a proposal from the national partnership for maternal safety. Journal of Obstetric, Gynecologic & Neonatal Nursing. 2014 Nov 1;43(6):771-9.

20. Singh, S., McGlennan, A., England, A. and Simons, R. (2012) 'A validation study of the CEMACH recommended modified early obstetric warning system (MEOWS)', Anaesthesia. Blackwell Publishing Ltd, 67(1), pp. 12–18. doi: 10.1111/j.1365-2044.2011.06896.x.

For peer review only - http://bmjopen.bmj.com/site/about/guidelines.xhtml

Appendix 1: **CFIR framework adapted from Damschroder LJ et al. for classification of outcomes**

Domains	Constructs
One: Intervention Characteristic	Intervention Source<br>Evidence Strength and quality<br>Relative Advantage<br>Adaptability<br>Trialability<br>Complexity<br>Design Quality and packaging Cost
Two: Outer Setting	Patient Needs and Resources<br>Cosmopolitanism<br>Peer Pressure<br>External Policies and Incentives
Three: Inner Setting	Structural characteristics<br>Networks & Communication<br>Culture<br>Implementation Climate<br>Tension for change<br>Compatibility<br>Relative priority<br>Organizational incentives and rewards<br>Goals and feedback<br>Learning climate<br>Readiness for implementation<br>Leadership engagement<br>Available resources<br>Access to knowledge and information
Four: Characteristics of Individuals	Knowledge and Beliefs about the intervention<br>Self-efficacy<br>Individual stage of change<br>Individual identification with organization<br>Other personal Attributes
Five: Process	Planning<br>Engaging<br>Opinion leaders<br>Formally appointed internal implementation leaders<br>Champions<br>External change agents<br>Executing<br>Reflecting and evaluating

For peer review only - http://bmjopen.bmj.com/site/about/guidelines.xhtml

Appendix 2: Interview Guide

Interview Guide

1. Intervention Characteristics

1. What do you know about the intervention or its implementation?
2. How different is this intervention from your existing practices?
3. What kind of information or evidence are you aware of that shows whether or not the intervention will work in your setting?
4. What kinds of changes or alterations do you think you will need to make to the intervention so it will work effectively in your setting?
    - Do you think you will be able to make these changes? Why or why not?
5. What is your perception of the bundling of the intervention for implementation and quality of the supporting materials? Prompts: format, design, user-friendly. Duration, scope, intricacy and number of steps

2. Outer Setting

6. How do you think the individuals served by your organization will respond to the intervention?
7. What barriers will the individuals served by your organization face to participating in the intervention?
8. What kind of local, state, or national performance measures, policies, regulations, or guidelines might be important in influencing how this intervention can be implemented?

3. Inner Setting

9. Can you describe how the intervention will be integrated into current processes?
10. What are your current guidelines to assess and manage patients with maternal sepsis? Probes: tool, framework or guidelines for maternal sepsis, lactate test
11. What is your knowledge about importance of lactate test and what is your current practice about lactate testing? Probes: implications for lactate test, guidelines for lactate test
12. What is your current patient to doctor and patient to nurse’s ratio in your setting?

For peer review only - http://bmjopen.bmj.com/site/about/guidelines.xhtml

13. Explain the role of doctors and nurses in management of maternal sepsis in your organization. Which cadre is responsible for care and at what level of care? Probes: nurses, doctors, technicians and other health care cadres

14. Other than human resources, what resources are utilized in management of maternal sepsis in your hospital?

15. Do you expect to have sufficient resources to implement and administer the intervention?

    ○ [If no] What resources will not be available? Probes: human resource, equipments, critical units etc

16. Do you feel the training planned for you will prepare you to carry out the roles and responsibilities expected of you? ○ What are the positive aspects of planned training? What is missing?

4. Characteristics of Individuals

17. How do you feel about the intervention being used in your setting?

18. Do you think the intervention will be effective in your setting? Why or why not?

5. Process

19. Who will lead implementation of the intervention?

20. Are there people in your organization who are likely to champion (go above and beyond what might be expected) the intervention?

    Prompts: Position of these champions have in your organization?

21. How do you think they will help with implementation?

For peer review only

Appendix 3- FAST-M bundle care tools and patient algorithm

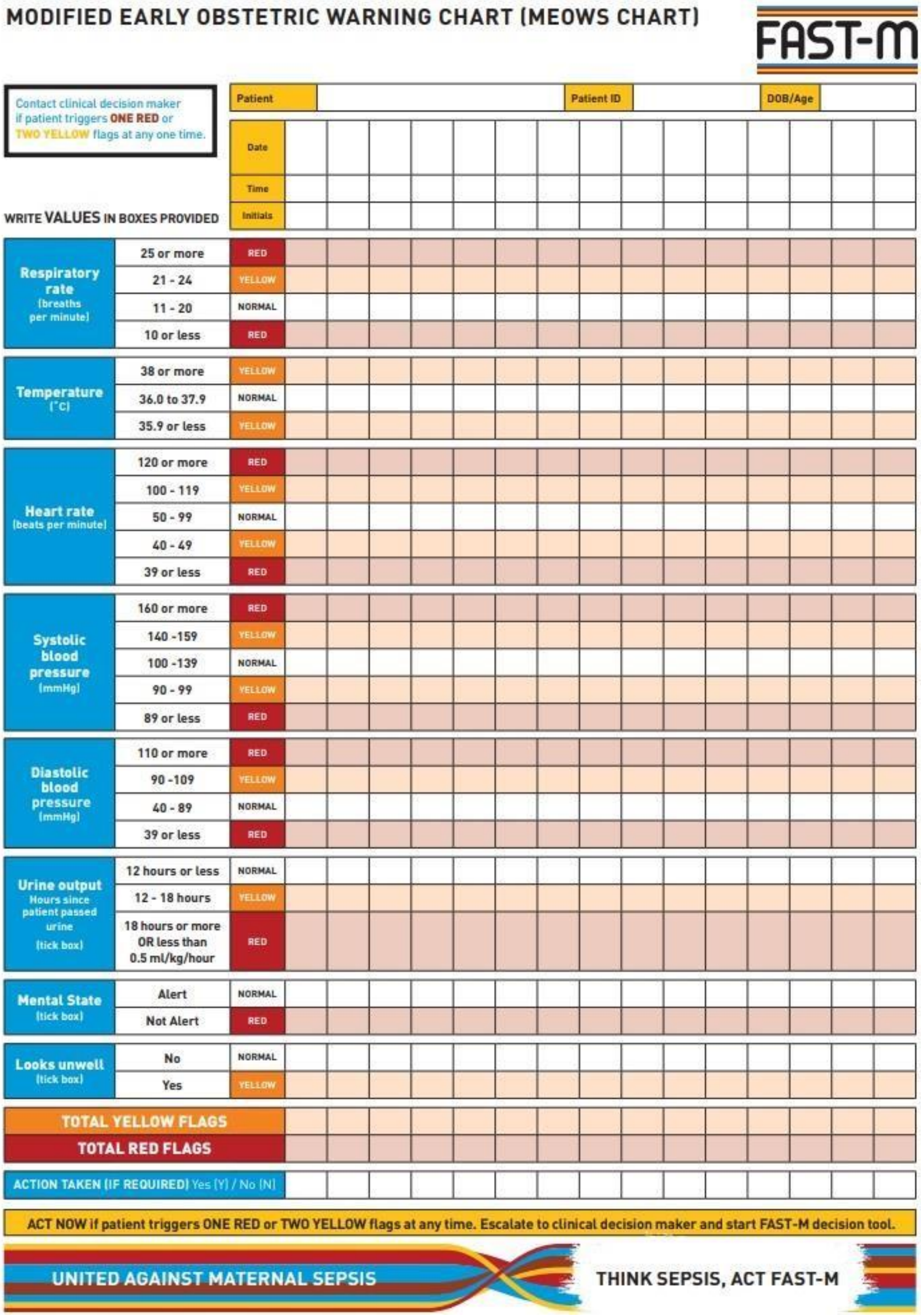

MODIFIED EARLY OBSTETRIC WARNING CHART (MEOWS CHART)

FAST-M

Contact clinical decision maker if patient triggers **ONE RED** or **TWO YELLOW** flags at any one time.

Patient		Patient ID		DOB/Age	

WRITE VALUES IN BOXES PROVIDED

Parameter	Value	Flag
		Date
		Time
		Initials
Respiratory rate (breaths per minute)	25 or more	RED
	21 - 24	YELLOW
	11 - 20	NORMAL
	10 or less	RED
Temperature (°C)	38 or more	YELLOW
	36.0 to 37.9	NORMAL
	35.9 or less	YELLOW
Heart rate (beats per minute)	120 or more	RED
	100 - 119	YELLOW
	50 - 99	NORMAL
	40 - 49	YELLOW
	39 or less	RED
Systolic blood pressure (mmHg)	160 or more	RED
	140 -159	YELLOW
	100 -139	NORMAL
	90 - 99	YELLOW
	89 or less	RED
Diastolic blood pressure (mmHg)	110 or more	RED
	90 -109	YELLOW
	40 - 89	NORMAL
	39 or less	RED
Urine output Hours since patient passed urine (tick box)	12 hours or less	NORMAL
	12 - 18 hours	YELLOW
	18 hours or more OR less than 0.5 ml/kg/hour	RED
Mental State (tick box)	Alert	NORMAL
	Not Alert	RED
Looks unwell (tick box)	No	NORMAL
	Yes	YELLOW
TOTAL YELLOW FLAGS		
TOTAL RED FLAGS		
ACTION TAKEN (IF REQUIRED) Yes (Y) / No (N)		

ACT NOW if patient triggers ONE RED or TWO YELLOW flags at any time. Escalate to clinical decision maker and start FAST-M decision tool.

UNITED AGAINST MATERNAL SEPSIS

THINK SEPSIS, ACT FAST-M

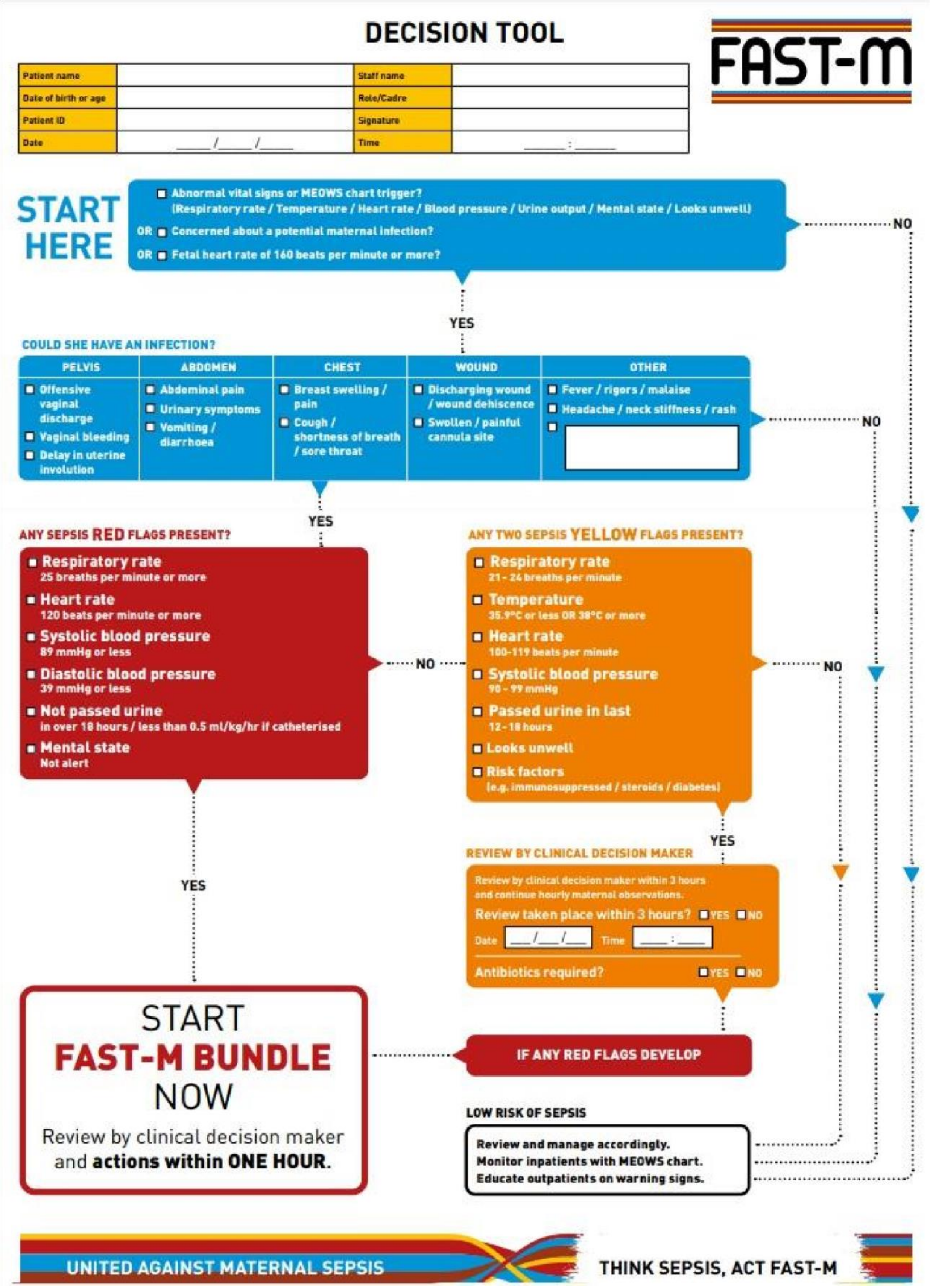

For peer review only - http://bmjopen.bmj.com/site/about/guidelines.xhtml

TREATMENT BUNDLE

FAST-M

Patient name		Staff name	
D.O.B or age		Role/Cadre	
Patient ID		Signature	

Date & time of red flag observation	__/__/__ __:__	Date & time bundle started	__/__/__ __:__	Date & time of review by clinical decision maker	__/__/__ __:__

1HR REMEMBER TO COMPLETE THESE ACTIONS WITHIN ONE HOUR

F — FLUIDS (caution in pre-eclampsia, severe anaemia and pulmonary oedema)

Date	__/__/__	Time fluids initiated	__:__	Initials	
Details / reason not completed					

Give 500 ml crystalloid immediately. Repeat 500 ml boluses to a maximum of 30 ml/kg if hypotension persists.

A — ANTIBIOTICS

Date	__/__/__	Time started	__:__	Initials	
Details / reason not completed					

See antibiotic guidelines below

S — SOURCE – identify and treat the source of infection

Date	__/__/__	Time considered	__:__	Initials	
Details / reason not completed					

See source identification and treatment boxes below

T — TRANSPORT (to higher level hospital or location within hospital, if required)

Date & time transport considered	__/__/__ __:__	Initials		Transport Required ☐ YES ☐ NO
Date & time transport requested	__/__/__ __:__	Initials		☐ N/A
Date & time patient left facility	__/__/__ __:__	Initials		
Destination				
Reason for any delay				

M — MONITORING (start MEOWS chart if not already started. Repeat observations every 30 minutes until otherwise decided by clinical decision maker)

Date & time monitoring commenced	__/__/__ __:__	Details / reason not completed
Maternal / fetal monitoring should include	• Respiratory rate<br>• Temperature<br>• Heart rate<br>• Blood pressure<br>• Urine output<br>• Mental state<br>• Fetal heart rate	
Neonatal monitoring and review commenced	☐ YES ☐ NO ☐ N/A	

ANTIBIOTIC GUIDELINES

Insert local guidance here

Immediate treatment for Maternal Sepsis:

- Ceftriaxone 2 g IV once daily (if no IV access this can be given as 2 IM injections of 1 g in different sites).
- If possible intra-abdominal source add Metronidazole 500 mg IV three times daily or 400 mg PO three times daily.

If above antibiotic regime is not available then give:

- Chloramphenicol 1 g IV/IM four times daily plus Gentamycin 240 mg IV/IM once daily.

If maternal infection source is known, or as soon as it is identified:

- Use specific treatment based on Malawi Standard Treatment Guidelines.

IDENTIFY THE SOURCE

Consider

- Clinical history
- Clinical examination
- Blood tests (if available) (FBC, U&Es, LFTs, CRP, clotting)
- Blood cultures
- HIV and Malaria testing
- Urine sample
- Swabs (wound, vagina, throat)
- Sputum sample
- Imaging (abdominal, chest)
- Lumbar puncture
- Other __________

REMOVE / TREAT THE SOURCE

Consider

- Malaria treatment
- Consider delivery of baby
- Removal of retained products of conception
- Debridement of wound / drainage of collection
- Removal of infected cannula / line
- Hysterectomy
- Targeted antibiotics once source known

UNITED AGAINST MATERNAL SEPSIS — THINK SEPSIS, ACT FAST-M

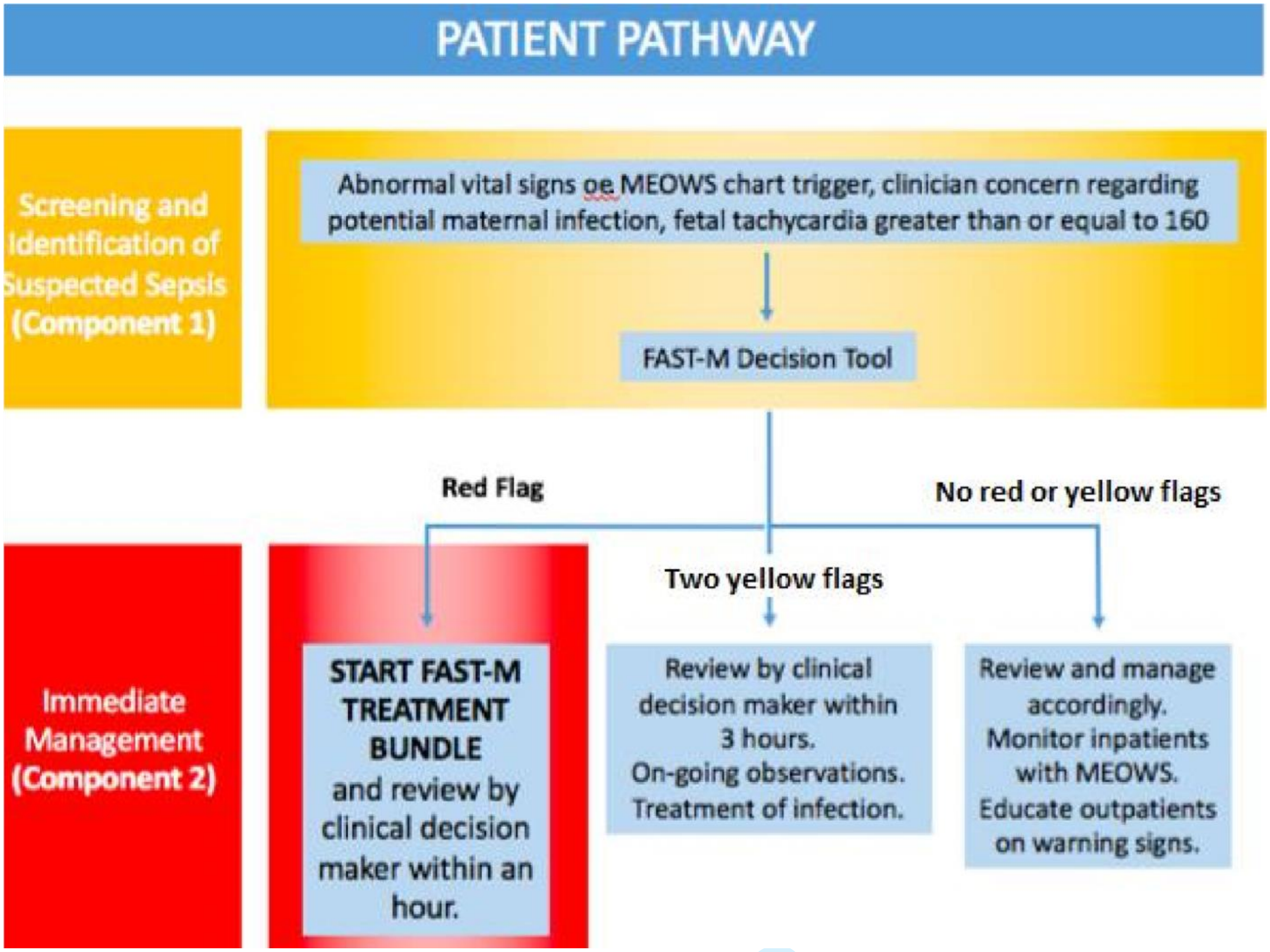

For peer review only - http://bmjopen.bmj.com/site/about/guidelines.xhtml

Appendix 4: Figure 1.

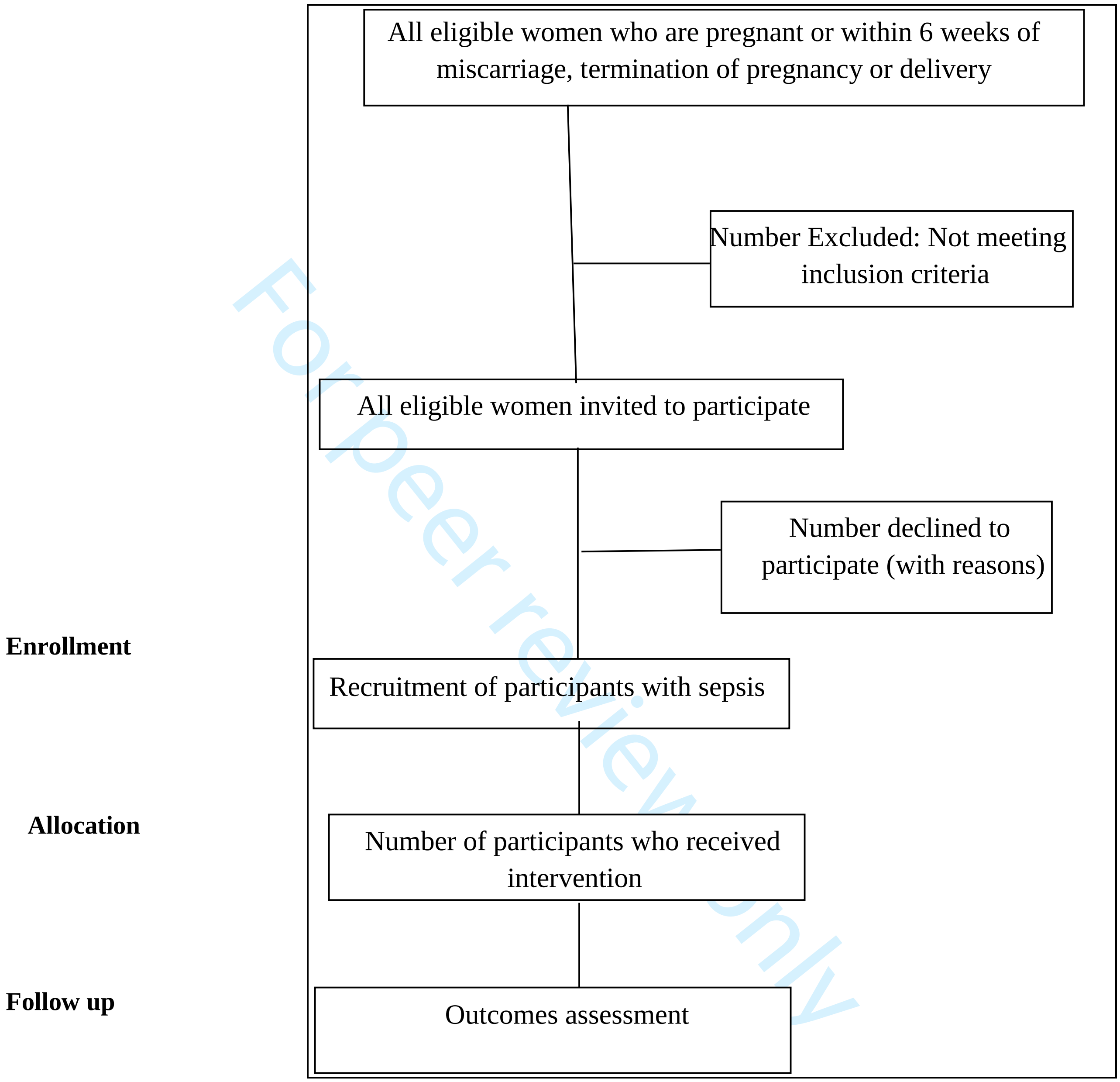

Fig. 1. Flow of participants through the study

For peer review only - http://bmjopen.bmj.com/site/about/guidelines.xhtml

Appendix 5: Figure 2

	STUDY PERIOD			
	**Enrolment**	**Allocation**	**Post-allocation**	**Close-out**
**TIMEPOINT**	$-t_1$	**0**	t_1	t_2
**ENROLMENT:**				
**Eligibility screen**	X			
**Informed consent**	X			
Baseline data collection	X			
**Allocation**		X		
**INTERVENTION:**				
Feasibility of FASTM bundle care tool			↔	
**ASSESSMENTS:**				
**/proportion of inpatients receiving a full set of vital signs on admission/**			X	
**/proportion of women with suspected maternal sepsis receiving the full FAST-M bundle/**			X	X
**/proportion of women with suspected maternal sepsis escalated to senior healthcare practitioners on the basis of abnormal vital signs/**			X	X

Fig. 2. Standard Protocol Items: Recommendations for Interventional Trials (SPIRIT) figure of enrolment, interventions and assessments

For peer review only

For peer review only - http://bmjopen.bmj.com/site/about/guidelines.xhtml

Appendix 6

Approaches	Planned Strategies
Facility level approaches	Site leadership by project champion,
	Formation of local sepsis committee
	Formal site launch
Individual level approaches	Multi-disciplinary, scenario-based local training
	Coaching by local project champion
	Aide-memoires, posters
	Paper-based tools (MEOWS chart, decision tool, treatment tool)
	Task sharing of vital sign measurement
Ongoing improvement approaches	Site based performance dashboards and run charts
	Local problem solving: led by sepsis committee (ongoing quality improvement, ownership, local adaptations, engagement, learning climate and sustainability)

Table 1. Summarised FAST-M implementation approach

For peer review only - http://bmjopen.bmj.com/site/about/guidelines.xhtml

BMJ Open

Adapting the FAST-M maternal sepsis intervention for implementation in Pakistan: A qualitative exploratory study

Journal:	*BMJ Open*
Manuscript ID	bmjopen-2021-059273.R1
Article Type:	Original research
Date Submitted by the Author:	30-Jun-2022
Complete List of Authors:	Ahmed, Sheikh; The Aga Khan University, Obstetrics and Gynecology<br>Khowaja, Bakhtawar; The Aga Khan University, Obstetrics and Gynecology<br>Barolia, Rubina; Aga Khan University,<br>Sikandar , Raheel ; Liaquat University of Medical and Health Sciences<br>Rind , Kubra ; The Aga Khan University, Obstetrics and Gynecology<br>Khan , Sehrish ; Liaquat University of Medical and Health Sciences<br>Rani , Raheela ; Liaquat University of Medical and Health Sciences<br>Cheshire , James ; University of Birmingham<br>Dunlop, Catherine; Birmingham Women's Hospital, Academic, 3rd Floor<br>Coomarasamy, Arri; University of Birmingham,<br>Sheikh, Lumaan; The Aga Khan University Hospital, Obstetric &Gynecology<br>Lissauer, David ; University of Liverpool
**Primary Subject Heading**:	Qualitative research
Secondary Subject Heading:	Infectious diseases
Keywords:	INFECTIOUS DISEASES, OBSTETRICS, Health & safety < HEALTH SERVICES ADMINISTRATION & MANAGEMENT, Public health < INFECTIOUS DISEASES, Reproductive medicine < GYNAECOLOGY

SCHOLARONE™
Manuscripts

For peer review only - http://bmjopen.bmj.com/site/about/guidelines.xhtml

I, the Submitting Author has the right to grant and does grant on behalf of all authors of the Work (as defined in the below author licence), an exclusive licence and/or a non-exclusive licence for contributions from authors who are: i) UK Crown employees; ii) where BMJ has agreed a CC-BY licence shall apply, and/or iii) in accordance with the terms applicable for US Federal Government officers or employees acting as part of their official duties; on a worldwide, perpetual, irrevocable, royalty-free basis to BMJ Publishing Group Ltd ("BMJ") its licensees and where the relevant Journal is co-owned by BMJ to the co-owners of the Journal, to publish the Work in this journal and any other BMJ products and to exploit all rights, as set out in our licence.

The Submitting Author accepts and understands that any supply made under these terms is made by BMJ to the Submitting Author unless you are acting as an employee on behalf of your employer or a postgraduate student of an affiliated institution which is paying any applicable article publishing charge ("APC") for Open Access articles. Where the Submitting Author wishes to make the Work available on an Open Access basis (and intends to pay the relevant APC), the terms of reuse of such Open Access shall be governed by a Creative Commons licence – details of these licences and which Creative Commons licence will apply to this Work are set out in our licence referred to above.

Other than as permitted in any relevant BMJ Author's Self Archiving Policies, I confirm this Work has not been accepted for publication elsewhere, is not being considered for publication elsewhere and does not duplicate material already published. I confirm all authors consent to publication of this Work and authorise the granting of this licence.

For peer review only

Paper Title:

Adapting the FAST-M maternal sepsis intervention for implementation in Pakistan: A qualitative exploratory study

Author Names:

Sheikh Irfan Ahmed[1]

Bakhtawar M.Hanif Khowaja[1]

Rubina Barolia[1]

Raheel Sikandar[2]

Kubra Rind[1]

Sehrish Khan[2]

Raheela Rani[2]

James Cheshire[3]

Catherine Dunlop [3]

Arri Coomarasamy[3]

Lumaan Sheikh[1]

David Lissauer[4, 5]

Affiliations & full institutional mailing addresses of all authors

[1] Aga Khan University Hospital, National Stadium Road, Karachi city, Pakistan. P.O. Box 3500 Postal code: 74800

[2] LUMHS Hospital Liaquat University of Health and Medical Sciences, Hyderabad city, Pakistan. Postal Code: 76090

[3] Institute of Metabolism and Systems Research, University of Birmingham, Edgbaston, Birmingham, UK, B15 2TT.

For peer review only - http://bmjopen.bmj.com/site/about/guidelines.xhtml

For peer review only

[4] Institute of Life Course and Medical Sciences, University of Liverpool, Liverpool, William Henry Duncan Building, Liverpool, UK, L7 8TX.

[5] Malawi-Liverpool-Wellcome Clinical Research Programme, Chichiri, Blantyre 3, Malawi

Email addresses

sheikh.irfan@aku.edu

bakhtawar.hanif@aku.edu

rubina.barolia@aku.edu

raheel.sikandar@lumhs.edu.pk

ghulam.kubra@aku.edu

drkhanhyd@gmail.com

raheela_rani@hotmail.com

james.cheshire@nhs.net

catherinedunlop@nhs.net

A.Coomarasamy@bham.ac.uk

lumaan.sheikh@aku.edu

David.Lissauer@liverpool.ac.uk

Corresponding Author*:

Sheikh Irfan Ahmed

For peer review only

For peer review only - http://bmjopen.bmj.com/site/about/guidelines.xhtml

Abstract

Objective

A maternal sepsis management bundle for resource-limited settings was developed through a synthesis of evidence and international consensus. This bundle, called "FAST-M" consists of: Fluids, Antibiotics, Source control, assessment of the need to Transport/Transfer to a higher level of care and ongoing Monitoring (of the mother and neonate). The study aimed to adapt the FAST-M intervention including the bundle care tools for early identification and management of maternal sepsis in a low resource setting of Pakistan and identify potential facilitators and barriers to its implementation.

Setting

The study was conducted at the Liaquat University of Medical and Health Sciences (LUMHS), which is a tertiary referral public sector hospital in Hyderabad.

Design and Participants

A qualitative exploratory study comprising key-informant interviews and a focus group discussion was conducted with healthcare providers working in the study setting between November 2020 and January 2021, to ascertain the potential facilitators and barriers to the implementation of the FAST-M intervention. Interview guides were developed using the five domains of the Consolidated Framework for Implementation Research (CFIR) framework: intervention characteristics, outer setting, inner setting, characteristics of the individuals, and process of implementation.

For peer review only - http://bmjopen.bmj.com/site/about/guidelines.xhtml

Results

Four overarching themes were identified, the hindering factors for implementation of the FAST-M intervention were: (I) Challenges in existing system such as a shortage of resources and lack of quality assurance; and (II) Clinical practice variation that includes lack of sepsis guidelines and documentation; the facilitating factors identified were: (III) Health care providers' perceptions about the FAST-M intervention and their positive views about its execution; and (IV) Development of HCPs readiness for FAST-M implementation that aided in identifying solutions to potential hindering factors at their clinical setting.

Conclusion

The study has identified potential gaps and probable solutions to the implementation of the FAST-M intervention, with modifications for adaptation in the local context

Keywords: FAST-M intervention, maternal sepsis, Pakistan, qualitative study, sepsis bundle, care bundle, complex intervention, low-resource setting, feasibility study.

For peer review only - http://bmjopen.bmj.com/site/about/guidelines.xhtml

Strengths and Limitations of this study

- The major strength of this study is the use of CFIR, which we used to gather data through the development of interview guides using CFIR domains.
- We collected data from multiple levels of HCPs using different methods of data collection i.e. individual interviews and focus group discussion to triangulate our findings and establish the trustworthiness of the study.
- The key informant interviews focused mainly on the doctor's perspective due to the prominent role of doctors in the study setting which limited us to gain perceptions of other healthcare providers.
- The study focused only on the perspective of the healthcare providers who have experience in the management and treatment of maternal sepsis patients to know the existing sepsis guidelines of the facility and adapt the intervention based on their experiences and feedback.

For peer review only - http://bmjopen.bmj.com/site/about/guidelines.xhtml

Background

Maternal sepsis is a major contributor to maternal morbidity and mortality worldwide [1]. Maternal sepsis is a life-threatening organ dysfunction caused by a dysregulated host response due to infection during pregnancy, childbirth and in the postpartum period [2, 3].

Globally, maternal sepsis accounts for about one tenth of maternal deaths and is the third most common cause of maternal mortality [1, 4]. It was estimated that each year 75,000 maternal deaths occurred in low and middle income countries due to maternal sepsis and approximately 10% of maternal deaths in Africa and Asia occur due to sepsis [4,5]. The risk of death among women who develop puerperal sepsis was higher in Africa (odds ratio 2.71), Asia (1.91), and Latin America and the Caribbean (2.06) than in developed countries [5].

Led by the World Health Organization and other partners, a global initiative was commenced in 2015, to develop strategies aimed at improving the early recognition and management of maternal sepsis [6]. Strategies to ensure early identification and treatment of sepsis have demonstrated significant improvement in outcomes in high income adult population settings [7] and it was necessary to translate these approaches into the maternity population and make them appropriate for low-resource settings [8]. Yet, there is very limited evidence of the implementation of such approaches specific to maternity care in low-resource settings.

Thus, a maternal sepsis bundle was developed as part of this process to improve the recognition and management of maternal sepsis in a low-resource setting. A modified Delphi approach was adopted to identify components significant to treatment and monitoring in terms of clinical importance and feasibility in resource-poor settings [9]. The components selected were: **F**luids, **A**ntibiotics, **S**ource control, assessment of the need to **T**ransport/Transfer to a higher level of

For peer review only - http://bmjopen.bmj.com/site/about/guidelines.xhtml

care and ongoing **M**onitoring (of the mother and neonate). The bundle was named "FAST-M" as a memorable acronym for both communication and awareness-raising [9].

Implementation of the FAST-M intervention across 15 government healthcare facilities in Malawi was found to not only be feasible but also resulted in improved clinical care [10], demonstrating that the intervention could assist in the early identification and management of maternal sepsis in low-resource settings [10]. This is now being tested formally as part of a large cluster-randomised trial across Malawi and Uganda.

In Pakistan, complications during pregnancy and childbirth are the leading causes of death in women, accounting for 20% of all deaths of women of child-bearing age [11-13]. National figures show that 15% of maternal deaths are reported due to sepsis [13] and maternal sepsis is established as the 3rd leading cause of maternal mortality [14]. Globally, the incidence of puerperal sepsis is 4.4% [11] whereas in Pakistan the incidence is reported to be 10-15% [15].

There are national sepsis guidelines for Pakistan (SGP) which are designed to aid in the identification and management of sepsis in adults in the local settings and are modeled on the Surviving Sepsis Campaign (SSC) [16]. However, these are inconsistently applied and lack a comprehensive implementation approach. There is still uncertainty about how best to optimise the implementation of evidence-based practices around maternal sepsis prevention and management in Pakistan.

The absence of routine monitoring in most public facilities in Pakistan during labor and childbirth such as not taking vital signs of women and newborns substantially increases the risk of maternal and newborn morbidity and mortality [17]. It has been evident that the quality of care is poorer in public referral facilities than in primary healthcare facilities [18]. Whilst the

For peer review only - http://bmjopen.bmj.com/site/about/guidelines.xhtml

FAST-M intervention when implemented in health settings of Malawi has shown improvements in vital signs recording and improved timely identification and management of women with maternal sepsis [9].

It is therefore planned to adapt and implement the FAST-M intervention in Pakistan. However, we recognise that to optimise its use in the Pakistani context requires a robust process of adaptation and re-design prior to its field testing. The implementation of the FAST-M intervention will be highly context specific. Therefore, this study aims to understand the existing sepsis management practices and behaviours to adapt the FAST-M bundle care tools in the local context. In addition, it will assist in the identification of the potential facilitators and barriers to its implementation in a low-resource setting within Pakistan.

This qualitative study was conducted in preparation for the implementation of FAST-M intervention in phase II of the study. The protocol and procedures for phases I and II of this study have been described in detail elsewhere [19]. The study findings obtained in this formative research will aid in the development of feasible methods to improve the processes and implementation of the FAST-M intervention in Pakistan.

Methods

Study Design

Our methods, grounded in implementation science, aimed to identify the anticipated facilitators and barriers in the implementation of FAST-M intervention at the Liaquat University of Medical Health Sciences (LUMHS), Hyderabad. Implementation research aims to identify the factors that function as barriers and enablers to specific interventions [20]. As our research question is descriptive and exploratory, this formative research adopted a qualitative research design

For peer review only

involving both focus group discussion (FGD) and key-informant interviews and a purposive sampling approach.

Focus group discussion (FGD) and key-informant interviews (KIIs) were conducted with health care providers working at the study site using interview guides structured using the CFIR framework [21]. The aim of FGD and KIIs was to engage health practitioners, government officials, and other key stakeholders to understand the behavior of existing practices in the study setting for maternal sepsis care, identify various facilitators and barriers that may influence the implementation of the FAST-M intervention and inform the adaptation of FAST-M bundle care tools and implementation approach according to the local context. Data collection through key informant interviews and FGD were to ensure data triangulation through different methods ensuring credibility of the study findings. The present study is being stated as per the guidance provided in consolidated criteria for reporting qualitative research (see online supplemental file 1).

Consolidated Framework for Implementation Research

The CFIR is a 'meta-theoretical' framework that provides an overarching analysis for implementation [21]. It offers an extensive and standardized list of constructs that allow researchers to identify various variables that are most relevant to a particular intervention [22]. The CFIR consists of five major domains: intervention characteristics, outer setting, inner setting, characteristics of the individuals, and the process of implementation. These domains are organized into 39 constructs (Table 1).

For peer review only - http://bmjopen.bmj.com/site/about/guidelines.xhtml

Table 1: CFIR domains and associated constructs

Domains	Constructs
**One: Intervention Characteristic**	Intervention Source<br>Evidence Strength and quality<br>Relative Advantage<br>Adaptability<br>Trialability<br>Complexity<br>Design Quality and packaging<br>Cost
**Two: Outer Setting**	Patient Needs and Resources<br>Cosmopolitanism<br>Peer Pressure<br>External Policies and Incentives
**Three: Inner Setting**	Structural characteristics<br>Networks & Communication<br>Culture<br>Implementation Climate<br>Tension for change<br>Compatibility<br>Relative priority<br>Organizational incentives and rewards<br>Goals and feedback<br>Learning climate<br>Readiness for implementation<br>Leadership engagement<br>Available resources<br>Access to knowledge and information
**Four: Characteristics of Individuals**	Knowledge and Beliefs about the intervention<br>Self-efficacy<br>Individual stage of change<br>Individual identification with organization<br>Other personal Attributes
**Five: Process**	Planning<br>Engaging<br>Opinion leaders<br>Formally appointed internal implementation leaders<br>Champions<br>External change agents<br>Executing<br>Reflecting and evaluating

For peer review only

For peer review only - http://bmjopen.bmj.com/site/about/guidelines.xhtml

CFIR has been used in various studies to inform qualitative processes across a range of complex intervention, because this flexible framework can be tailored to different settings across multiple contexts [21,22]. We therefore used the tailored CFIR framework to understand critical barriers and facilitators to implementation of FAST-M intervention that need to be addressed at multiple levels if the FAST-M intervention is to be successfully optimised, and adopted in healthcare practices in Pakistan.

Study setting

Liaquat University of Medical Health Sciences (LUMHS) is located in Hyderabad district, Pakistan. LUHMS is 1300 bed tertiary referral public sector hospital which serves a large number of mostly underprivileged populations. The hospital offers various facilities for both in-patient and out-patient. The hospital has three Obstetrics and Gynecology units and provides 24 hours emergency cover to patients coming from urban and rural areas of Sindh. It manages a high volume of cases of maternal sepsis every month. The current data from the facility shows that a total of approximately 11205 patients were admitted to OBGYN units from the period of January to August 2021, and the maternal mortality rate was recorded as 159/11205 (1.4%). Out of these 159 deaths, 45 were due to confirmed maternal sepsis (28.3%). These indicators direct that there is a need for a robust system to early detect and manage maternal sepsis cases in the hospital.

Patient and public involvement

There was no patient or public involvement in setting the research agenda.

Data collection methods and study participants

Healthcare providers working at LUMHS hospital were purposively sampled for KIIs and FGD. The letters of invitation were sent to all healthcare providers including Doctors (residents and

For peer review only - http://bmjopen.bmj.com/site/about/guidelines.xhtml

faculty members), staff nurses, and administrators who were involved in the management and treatment of maternal sepsis patients for at least the past six months from the time of invitation. All the participants who were approached by the study team agreed to participate in the study. The aim of KIIs and FGD was to explore and understand the behavior of the existing practices and guidelines used in the hospital for sepsis management, and an appropriate system for characterising intervention and its components that can make use of this understanding. KIIs with healthcare providers were conducted in the meeting room and faculty offices at LUMHS hospital. A FGD was conducted in the seminar room at LUMHS hospital. A trained moderator facilitated the focus group discussion. Interviews were scheduled according to participants' preferences and were audio-recorded following consent from study participants (Supplemental file 2).

Data collection procedure

A semi-structured interview guide was developed to explore healthcare professionals' views and attitudes towards the FAST-M intervention (Supplemental file 3), with a focus on the views on the feasibility of FAST-M implementation among healthcare professionals using five major domains of CFIR: intervention characteristics, outer setting, and inner setting, characteristics of the individuals and the process of implementation. The interview guides were tailored considering each category of participants. The research team reviewed the interview guide for content and flow and trialed the guide for the length of time and appropriateness of the questions. Before beginning the interview, the qualitative researchers first described the FAST-M bundle components and the patient referral pathway (supplemental file 4) demonstrating the utilization of FAST-M bundle care tools. The interview guide underwent subsequent modifications and iterations based on interviews conducted.

For peer review only - http://bmjopen.bmj.com/site/about/guidelines.xhtml

A free flow of information was encouraged, using probes from these discussions to obtain healthcare professionals' perceptions about the adaptation and feasibility of the FAST-M intervention. Interviews were conducted face-to-face in Urdu and English (KIIs = 16; FGD =1). The standards of precautions for control of COVID-19 infection were followed during data collection. All study participants were screened before interviews for COVID-19 infection through a series of questions regarding their symptoms. The participants were asked to wear masks at all times during interviews and discussions. The focus group discussion was conducted in a large seminar room to maintain physical distance between participants as a precaution for control of COVID-19 infection.

Interviews and focus group discussion were conducted by RB, SI, BK, and GK, who are part of the investigating team and are trained in qualitative research. The research questions were based on FAST-M intervention characteristics, outer and inner health care setting, characteristics of the individuals, and the process of implementation. Detailed field notes were taken during each interview to capture non-verbal language and cues. KIIs were conducted for 20 minutes to 40 minutes; FGD was conducted for 50 minutes and consisted of 12 participants in a group. Data were collected using interview guides developed on five major domains of CFIR: intervention characteristics, outer setting, inner setting, characteristics of the individuals, and the process of implementation. Data were collected and analyzed through an iterative process. The data collected through interviews and discussion were carried out until data saturation was achieved and no new information emerged [23]. We defined saturation as the amount of data needed until nothing new information and a meaningful conclusion drawn out about the feasibility of the FAST-M intervention was apparent and redundancy was reached.

For peer review only

For peer review only - http://bmjopen.bmj.com/site/about/guidelines.xhtml

Data Analysis

Study data were analyzed using a conventional qualitative content analysis approach facilitated by NVivo version 10 (QSR International, Pty Ltd) software. First, all the audio recordings were translated and transcribed from the local language (Urdu) into English. Transcripts were read several times to develop an interpretation of the participants' views about the feasibility of FAST-M implementation. Focus group discussion and KIIs were coded as one data set. Two investigators coded a subset of transcripts independently using separate coding that was then combined to match codes, and agreement by investigators was sought on a coding framework. Codes were formulated inductively from the transcripts related to research questions and CFIR domains. Coding discrepancies were discussed and resolved to reduce researchers' biases. Codes were then analyzed into categories and then the major themes based on the data findings.

The potential barriers and facilitators and modifications in the bundle care tools were identified that were discussed and reviewed by the research team. To ensure the credibility of the research, study data were triangulated by different data sources including doctors, nurses, and administrators and through different data collection methods including FGD and KIIs, to compare alternative perspectives and to assess any inconsistencies. The hospital leadership and a subgroup of clinical care providers were directly contacted and invited to attend an interactive session to hear about the findings and reflect on whether these were considered representative of their existing practices prior to modifying the bundle care tools and adapting the intervention. This respondent's validation process enhanced rigor and established conformability [24].

For peer review only - http://bmjopen.bmj.com/site/about/guidelines.xhtml

Results

In this qualitative study, one FGD and sixteen KIIs (Table 2) were conducted with HCPs (doctors, nurses, and health administrators), between November 2020 and January 2021who were involved in the management and treatment of maternal sepsis patients. Table 3 & 4 present demographics of study participants. A baseline facility audit was alongside conducted to identify the availability of resources in the facility (supplemental file-5). The survey findings assisted the study team to plan a practical approach for the implementation of the intervention (the audit findings will be recorded elsewhere). The qualitative findings presented in this paper aided the validation of observational findings. This helped the study team to gain feedback and insights from healthcare providers about their existing sepsis guidelines and resource availability. Based on these findings, the bundle care tools will be modified before implementation and the feasibility assessment.

Table 2: Study participants

Focus group discussion with HCPs	Total FGD=1; n=12
**Doctors (Medicine); (OBGYN)**	n=3; n=5
**Nurses (OBGYN); (labor room)**	n=1; n=1
**Health administrators**	n=2
Key informant interviews	**Total KIIs= 16; n=16**
**Doctors (OBGYN); (Operating room); ICU**	n=8 ; n=1; n=2
**Nurses (OBGYN)**	n= 4
**Health administrators**	n= 1

For peer review only - http://bmjopen.bmj.com/site/about/guidelines.xhtml

Table 3: Demographics of participants in KIs

**KIs**	**N=16**
**Job Title**	
Faculties from obstetrics and gynecology (Professor, Associate &Assistant Professors)	3
Faculties from obstetrics and gynecology (Professor, Associate &Assistant Professors)	1
Registrars, Residents & Medical Officers (OBGYN)	5
Residents & Medical Officers (Family Medicine)	2
Registered nurses	4
Administration staff	1
**Working experience in facility**	
>10 years	7
> 5 years	6
1- 5 years	3
**Gender**	
Male	4
Female	12
**Role in the hospital**	
Administration	2
Leadership	3
Clinical practices	11

Table 4: Demographics of group participants

**FGD participants**	**N=12**
**Job Title**	
Faculties from obstetrics and gynecology (Professor, Associate &Assistant Professors)	5
Faculties from family medicine (Professor, Associate &Assistant Professors)	3
Registered nurses	2
Administration registrars	2
**Working experience in facility**	
>10 years	5
> 5 years	5
1- 5 years	2
**Gender**	
Male	4
Female	8
**Role in the hospital**	
Administration	2
Leadership	5
Clinical practices	5

For peer review only

For peer review only - http://bmjopen.bmj.com/site/about/guidelines.xhtml

307 Data analysis revealed four overarching themes: (I) Challenges in existing system; (II) Clinical
308 practice variation; (III) Health care providers' perceptions about FAST-M; and (IV)
309 Development of HCPs readiness for FAST-M implementation. Table 5 demonstrates the
310 identified themes and categories.

311 Table 5: Themes and Categories

Themes	Categories
**Challenges in existing system**	Shortage of HCPs in the hospital
	Lack of adequate resources and quality assurance
**Clinical practice variation**	Sepsis guidelines and documentation
	Individual care practices and HCP comfort levels
**Health care providers' perceptions about FAST-M**	Understanding of the FAST-M bundle
	Perceptions about significance of FAST-M
	Identifying solutions to the application of FAST-M
**Development of HCPs readiness for FAST-M implementation**	Understanding and identifying gaps
	Consensus building for FAST-M implementation

312

313

314

315

316

317

For peer review only - http://bmjopen.bmj.com/site/about/guidelines.xhtml

Challenges in existing system

a. Shortage of HCPs in the hospital

A majority of the study participants reported challenges in the existing sepsis management practices. The major challenge reported by HCPs is the increased volume of patients coming to the obstetrics and gynecology inpatient wards and emergency room. The increased number of patients exaggerates the workload on health care providers. The issue of a high patient to doctors' ratio that is 6:1; and a high patient to nurses' ratio that is 20:1 was raised by a majority of study participants. There is a shortage of health workforce considering the influx of patients in the unit which is a hindering factor for provision of quality healthcare services.

"Being a tertiary level hospital, being a civil hospital and the main hospital, we are facing an increase patients flow on daily basis" (KII- Senior Registrar- OBGYN)

"On floor, we have 6 doctors and you think how many patients are there. Sometimes we have 36 admissions; sometimes we have around 40 admissions. So, you can see for doctors to patients ratio it is around 6:1 and for staff, they are sometimes present and sometimes not" (KII- Senior Registrar)

Health care providers identified that there is a considerable shortage of nurses in the hospital for the care of patients. The importance of nurse's role was acknowledged by all the key informants and focus group participants, and they emphasized the shortage of nurses for sepsis management in the hospital as a key challenge, with only one or two nurses assigned to 20 patients in each shift.

As it was stated:

For peer review only - http://bmjopen.bmj.com/site/about/guidelines.xhtml

"Yes we are short of staff nurses. Look, if we have around 32 to 40 patients so there is only one nurse for their care or hardly two" (KII- Staff Nurse)

"In emergency room, we do not have staff nurses available, so the doctor is responsible for maintaining IV line and catheterization. If there will be staff nurses available in the ER so they can help us with IV line, sending lab investigations and with catheterization. But this is a bitter truth that we have shortage of staff. No doubt the staff present in wards does work like they do patient's monitoring, IV medications and follow doctor's instructions" (KII- Admin Registrar)

b. Lack of adequate resources and quality assurance

Health care providers, mainly doctors, and nurses working in the hospital, voiced concerns over the scarcity of resources. All HCPs indicated their workplace as a low-resource setting and described private hospitals as having "*more resources than us*". Despite the disparity in resources, HCPs generally believed they were maximizing sepsis management within the limits of what was possible in their unit.

"…this is not a private hospital and unit like that. This is civil hospital and we have to face many things. Our surroundings are not as favorable as it seems. We have to struggle a lot and this is the cause of delay in things. But anyways, we are trying our best to manage sepsis cases within our available resources" (KII- Registrar Admin)

A majority of the patients present with complications and require intensive monitoring. There are High Dependency Units (HDUs) and Intensive Care Units (ICUs) in the hospital for critical monitoring of the patients though the shortage of spaces in HDU and ICU is a challenge, as reported by the study participants.

For peer review only - http://bmjopen.bmj.com/site/about/guidelines.xhtml

"We have monitors available but not according to the patients need. We cannot monitor all the patients and we do it according to the severity of patient's condition. We have only two HDU beds and this is a challenge for us" (KII- Senior Registrar)

"We have 12 surgical and 12 medicine beds in ICUs altogether in LUMHS for all units. We face constraints of getting ICU beds for critical patients" (FGD- HOD)

The obstetrics and gynecology units have their own set of routines or guidelines that help HCPs organize their practices and influence how and when care is provided. When asked about barriers and enablers in sepsis management, HCPs talked about the lack of awareness of policies that made it difficult to identify and manage sepsis cases. This concern was raised by a few key informants that a number of HCPs working in the facility are unaware of the hospital policies. Though all the key informants noted the presence of policies and guidelines for sepsis management, only a few (6/16) key informants had detailed knowledge about the policies or guidelines related to sepsis management. The other departments in the hospital example medical ICU, surgical ICU, labor room, emergency room, and inpatient wards follow different guidelines for sepsis management. This hinders the care given to patients because no unified system or protocol exists in the facility for sepsis management.

Few people know the correct knowledge of sepsis. People should refresh their knowledge and there should be combined meetings of all units so we have a protocol for CVP lines, high flow oxygen administration and antibiotics. There should be a set vision for this" (KII- Senior Registrar)

It was also reported by health administrator of the facility that the non-performance and non-seriousness of HCPs towards their job responsibilities is an impeding factor in sepsis

For peer review only - http://bmjopen.bmj.com/site/about/guidelines.xhtml

management. This non-performance and non-seriousness is the result of frustration and burnout caused due the HCPs workload.

"Our doctors are in a hurry to quickly complete their work and go, because they have a lot of burden" (KII- Healthcare Administrator)

All HCPs stressed on compromised quality of resources available in the facility. They reported that the quality and efficiency of antibiotics are lacking and there are hurdles in the obtainability of antibiotics. This delays patients' management and the patient care process.

"The most important is the below standard antibiotics provided here" (FGD- Associate Professor OBGYN)

This is honest truth that the antibiotics we get from outside, from a good company, there is a difference in the quality and efficiency. We are not getting good results with antibiotics as we are supposed to" (KII- Senior Registrar)

HCPs also highlighted the constraints faced from the level of patients. The collection and transport of blood samples to laboratories is a complicated process. The patient's samples are transferred to laboratories by the hospital staff at the selected time of the day. If any patient's investigation is required after that fixed set time, it is transferred to laboratory through patients' attendants. Consequently, this delays patients' investigational process.

"We have developed a system that in morning, the ward boy will collect samples from each ward, it goes to university hospital which doesn't charge anything. If any sample is missed and sent later, we send them through patient's attendants and they are charged" (KII-Health Administrator)

For peer review only - http://bmjopen.bmj.com/site/about/guidelines.xhtml

404 HCPs also deliberated on patient's ability to afford for lab investigations. Most of the patients coming to the facility belong to the low-income class group considering their socio-economic background. Though LUMHS is a public health facility and a majority of services are provided in the hospital without charge, there are few investigations for which patients are required to pay fees for services for example blood culture and serum lactate tests.

409 *"Our patients are poor and they cannot afford investigations like culture test and serum lactate. They are costly so people are reluctant for these blood tests" (KII- Registrar)*

411 *"These investigations should be free for patients. Culture bottles are so expensive and people are so poor that they go and throw them away" (FGD- Registrar Admin)*

413 **Clinical practice Variation**

414 a. Sepsis guidelines and documentation

415 The interview participants reported that the obstetrics and gynecology units follow Royal College of Gynecology (RCOG) guidelines. The RCOG guiding principles provide information about the risk factors of maternal sepsis, the basic vital signs and identification of maternal sepsis, clinical features suggestive of sepsis, investigations to rule out maternal sepsis, and the specific antimicrobial therapy for management [25]. Despite the presence of guidelines in the hospital, the early identification and management of sepsis is a huge struggle.

421 *"MEOWS chart was there in RCOG guidelines and we used to do that, but as you have these FAST-M tools, we didn't use to do this way. We used to do this very haphazardly" (KII- Assistant Professor)*

For peer review only - http://bmjopen.bmj.com/site/about/guidelines.xhtml

The **F** in the pneumonic of FAST-M denotes fluid resuscitation. This administration of intravenous fluids can be a key intervention for management of sepsis if it is associated with hypotension, however, rapid fluid administration is more complex in pregnant women if there are other co-existing medical problems such as eclampsia. These concerns and delays in fluid administration in the existing system were identified by HCPs. This delay was because of the HCPs anticipated apprehensions and concerns related to complications of fluid therapy as stated:

> *"In existing practices, we are giving the antibiotics but this fluid therapy sometimes gets delayed as we are concerned about the development of pulmonary edema in septic patients after giving fluids" (KII- Registrar)*

> *"Sometimes these gynae people get worried that whether it is sepsis or cardiac issue and whether we should give fluids or not as the patient can have fluid overload" (FGD- Assistant Professor- Medicine)*

Most of the study participants stated that they are following similar procedures and guidelines as provided in FAST-M bundle care tools. Yet, they identified a lack of documentation in the existing practices.

> *"We do not follow the step wise procedure and documentation but we follow the same thing as we do respiratory rate, BP, GCS and etc." (KII- Fellow-ICU)*

b. Individual care practices and HCP comfort levels

There is a hierarchy of doctors in the hospital from senior to junior level based on their qualifications and experience. The hospital units are managed by Professors who are Head of Department of the units. The upper category in the hierarchy of doctors comprises all the faculty staff including associate professors and assistant professors, the second upper category in the

For peer review only - http://bmjopen.bmj.com/site/about/guidelines.xhtml

hierarchy covers registrar doctors, who support postgraduate residents and house officers who come for their internship program following completion of medical training. These all categories of doctors have diverse job roles for the management of patients as stated:

> *"We have faculties and we have them on senior level, then we have our Registrars, PGs and HOs, so suppose senior level look for all the patients, do patients rounds and check and advice for the patients. Registrars have their assigned patients' beds. The registrars are assigned according to the number of beds present and occupied. These registrars are accompanied by PGs. Suppose, if any registrar is assigned 12 beds, she gets two PGs who can look after 6-6 beds. So the main people who are on the floor are registrars and PGs who manage patients according to the faculty's advice" (KII- Associate Professor)*

Within the hospital, it was observed that HCPs' approach to sepsis management was not consistent. Clinical practice variation refers to patients receiving differing care depending on when, where, and by whom they are being cared for, despite evidence for best practice. One HCP noted that:

> *"Some doctors send lactate and culture test and others don't... this may be because of patient's financial affordability. And this variation is also there when we prescribe antibiotics. Every doctor has their own practice" (KII- Registrar)*

Some nurses voiced concerns about timely management of patients. HCPs reported that patients monitoring gets delayed based on an individual nurse's levels of comfort to monitor the patients. There are less skilled nurses in the unit to identify and assess the criticality of the patient. The novice nurses are inexpert to take care of the patients and they also lack skills towards sepsis care.

For peer review only - http://bmjopen.bmj.com/site/about/guidelines.xhtml

"Senior nurse makes the schedule and look after the labor room as well as ward because of their competencies. We have new nurses as well but it is obvious that their understanding and knowledge of the work is less than ours" (KII- Staff nurse)

"We get senior and competent nurses in the morning shift because there is more work in morning shifts" (KII- Senior Registrar)

Unit practice norms, combined with the HCPs' personal comfort, confidence, and skills, inform their practices about sepsis management. HCPs also have varying definitions and criteria for which patients are transferred to ICUs and to sort this process uninterrupted, HODs decide on the eligibility criteria for admission to ICU.

Health care provider's perceptions about FAST-M

a. Understanding of the FAST-M bundle

HCPs reported that they were informed about FAST-M bundle care tools from their head of departments who are keen to test this intervention in their local setting. Some health care providers had more opportunities to learn about the components of FAST-M bundle, but other HCPs specifically staff nurses did not know about the FAST-M tools. While all doctors reported having a baseline understanding of FAST-M tools and its components including MEOWS chart, decision tool and treatment tool, they expressed the need of additional understanding of FAST-M tools before its implementation. All HCPs recommended providing additional education and training sessions to HCPs to address such gaps.

"Whatever HCPs are doing, they are doing at their own, they are also trained but they are not very well trained, so training will help them to manage patients well according to the guidelines" (KII- OR Doctor)

For peer review only - http://bmjopen.bmj.com/site/about/guidelines.xhtml

Healthcare administrators and doctors employed at the hospital displayed their interest in support for implementation of FAST-M intervention, whereas nurses most frequently cited satisfaction with their existing practices.

> *"Our OBGYN doctors are already providing us the charts for monitoring of cesarean deliveries, for baby's monitoring and there are different charts for monitoring. We are already managing our patients well" (FGD- Nurse)*

Majority of the key-informants highlighted positive influences of implementation of FAST-M bundle care tools on existing policies of sepsis management in the hospital as one of them stated:

> *"There is no current guideline followed in the hospital and this has come as a sort of guideline that can be used for sepsis management" (KII- OR Doctor)*

b. Perceptions about significance of FAST-M

HCPs attitudes towards FAST-M implementation were positive and supportive. All HCPs shared positive perceptions about timely sepsis identification and management through classification of patients using MEOWS chart's triggers as red and yellow flags. The use of colors such as red flags and yellow flags indicating cutoff values facilitates HCPs in identifying and categorizing patients. HCPs identified color demonstration in the MEOWs chart as a major enabler in identification of sepsis patients.

> *"Now we know that there is a red and yellow flag, and if patient is in severe sepsis we have to send the samples within an hour and have to give antibiotic and fluids as described in the protocol" (KII- Registrar)*

For peer review only

For peer review only - http://bmjopen.bmj.com/site/about/guidelines.xhtml

“It is very easy because of colors we are getting alert on red and yellow flags. This is very easy and understandable” (KII- Senior Registrar)

HCPs believed that FAST-M tools improve knowledge of HCPs as the tools include everything related to the identification and management of the patients with maternal sepsis. The flow of the tools was appreciated by HCPs and they also stated that this organized flow of FAST-M tools will save time in sepsis management.

“This tool provides specifications about fluid therapy and antibiotics administration with specific time. It has improved our knowledge” (KII- Nurse)

HCPs also indicated the significance of FAST-M tools as being initiated by any healthcare provider including the nurse. There is no requirement of a doctor to initiate the bundle care tools. The staff nurses and even the trainee dispensers, who are available in the unit as helpers to staff nurses, can initiate the MEOWs chart for identification of the cases.

“The good thing I see in this FAST-M is that even the nurse can start this bundle care” (FGD- HOD Gynae)

Generally, most HCPs stated that the FAST-M intervention will help in sharing tasks between HCPs and it will increase the accountability of HCPs to perform their responsibilities

“It should be done because from staff till doctor everybody will be responsible for their work and will document each and every thing. We get tired of emphasizing this” (KII- ICU Fellow)

For peer review only - http://bmjopen.bmj.com/site/about/guidelines.xhtml

One of the KIs emphasized the quality of this tool as being non-invasive. Patients would easily accept this intervention and HCPs would not hesitate to initiate it. It can be easily accepted and implemented.

> *"The intervention that has been introduced, it is totally non-invasive and it is the same work that we do in our daily routine, so we will have no problems in its implementation" (KII- ICU Fellow)*

All the key-informants and focus group participants articulated patients' benefits through FAST-M implementation. They emphasised that the early identification and management of maternal sepsis through the FAST-M tools may decrease patients' length of stay in the hospital, and eventually decreasing the length of stay would benefit patients in providing physical, economic and psychological advantages. Ultimately, this would help in decreasing maternal morbidities and mortalities in the long run.

> *"…it will benefit patient that it will help in decreasing the stay of patients and their exposure will be reduced. This will reduce morbidities and mortalities in the long run" (KII- Registrar)*

c. Identifying solutions to the application of FAST-M

Some HCPs were doubtful of the practicality of intervention in the prolonged and continuous implementation due to resource restrictions (e.g. quality of available antibiotics, shortage of staffing, shortage of equipment and monitors). The inability to overcome these limitations led to a common attitude that:

For peer review only - http://bmjopen.bmj.com/site/about/guidelines.xhtml

"Nothing is sufficient from top to bottom, we try our level best to provide but we do not have monitors, we have hurdles for lab investigations, there are issues of availability of nurses and antibiotics, there are many technical gaps" (KII- Registrar Admin)

All respondents suggested that in order to strengthen the significance of FAST-M intervention for early identification of sepsis, the inclusion of the variable of oxygen saturation in the MEOWS chart, with appropriate cut off values, would be important. This was because pulse oximetry is now available routinely in the unit and may be an important indicator of clinical deterioration. This feedback was consistently given by all HCPs.

"Oxygen saturation is mandatory to include in the MEOWs chart for monitoring of patient" (FGD- Assistant Professor- Medicine)

It was informed through HCPs working in the medicine unit that sepsis guidelines followed in their unit include an addition of steroid therapy and inotrope support for sepsis management.

"You should include support because sometimes when we give fluids and antibiotics, but still patient is not maintaining the blood pressure because most of the times septic patients arrives late, so you should include source plus support in S. so both of the things will be included. Because support is the most important" (FGD- Assistant Professor- Medicine)

All HCPs agreed over the use of ceftriaxone as first choice of antibiotics in FAST-M treatment bundle based on its cost and availability for patients.

"We give Ceftriaxone straight away as it is freely available. We give 2g Ceftriaxone and for those patients whose culture is sent, we wait for their blood culture reports to change

For peer review only - http://bmjopen.bmj.com/site/about/guidelines.xhtml

antibiotics accordingly. Otherwise, our patient mostly responds to ceftriaxone" (KII-Senior Registrar)

Few participants specified that they use Piperacillin/tazobactam and meropenem for management of the confirmed cases of sepsis due to their beneficial results in such patients, yet the patients pay out of pocket for the cost of these antibiotics. Thus, Meropenem and Piperacillin/Tazobactam were proposed as the second choice of antibiotics due to their availability and cost.

"...sometimes when we do not have availability of meropenem so we give ceftriaxone to the patients, which is easily available free of cost for patients" (KII- Senior Registrar)

HCPs also suggested involving nursing interns and trainee dispensers who come for their training and work without wages. The involvement of nursing interns and trainee dispensers would reduce the problem of shortage of staffing in the unit and they would be employed to implement the FAST-M intervention without added investment for human resources.

"We get one or two girls from BScN programme, but we can talk to the dean in account and there are many people who can help us with this" (FGD- Health Administrator)

The focus group participants identified the need of increasing awareness which is the key to implementation of the FAST-M intervention. The stakeholders emphasized understanding of HCPs about the significance of FAST-M bundle care tools as a key to effective implementation in future. One of the group participants suggested:

"We can make big boards and we can involve everyone and give them awareness. And we can provide examples to them that how it was implemented in past in different setting showing good outcomes" (FGD-HOD Gynae)

For peer review only - http://bmjopen.bmj.com/site/about/guidelines.xhtml

Moreover, the inclusion of MEOWs charts in patients' Medical Record files of the hospital was emphasized by every group member involved in the discussion.

> *"We will include MEOWS chart in all patients' files so our doctors can easily record the findings on MEOWS chart which will alert them about patient's condition" (FGD- HOD Gynae*)

HCPs readiness for FAST-M implementation

The HCPs readiness towards FAST-M intervention started with the drive of identification of requirements for FAST-M adaptation and concluded with the consensus building of HCPs for its implementation.

a. Understanding and identifying gaps

HCPs acknowledged that successful implementation of the FAST-M intervention would require health care facility to be well-equipped, including both the availability of equipment and trained health care providers. Other key challenges to the successful implementation of FAST-M intervention are related to logistics, including shortage of human resources and inadequate funds for procuring monitors for assessments, antibiotics and lab investigations. One of the most frequent concerns around FAST-M implementation included the need to train HCPs including doctors, nurses, and auxiliary support staff to enable them to set up and sustain the services. Further, study participants suggested that a multidisciplinary approach would be useful to ensure that all professionals including the team of doctors, nurses, administrators from different units e.g. medicine, intensive care units, labor room, laboratory and operating room are working together for the successful implementation of FAST-M.

For peer review only - http://bmjopen.bmj.com/site/about/guidelines.xhtml

"In team, one person should be from administration, to who if we complain related for our hurdles and queries, so he can work on them, one person should be from laboratory, one should be from nursing staff and one should be from doctors, who can take all the things to higher levels and work on them" (KII- Registrar Admin)

Healthcare providers argued that there are high costs associated with the implementation of FAST-M intervention. Providers further explained that high costs of laboratory investigations would be a limiting factor as it would cause additional anxiety of financial burden to the patients. On the other hand, a few health professionals confirmed that costs would not be a major concern if there was buy-in from hospital administration for the patient's requirements. HCPs mentioned that the initial investments may be higher for procuring required equipment like monitors and apparatus required for monitoring of patients.

"Ceftriaxone is easily available in our hospital, but we are not sure about its quality. But for the critical patients if we see any red flags, we can arrange their requirements from our donations. In our unit, we are doing this for critical patients" (FGD-HOD-Gynae)

b. Consensus building for FAST-M implementation

The focus group participants displayed readiness for implementation of FAST-M interventiom in their local context by developing consensus on resolutions and approaches to the perceived challenges they could encounter during the implementation. The focus group discussion provided the opportunity to reflect on the anticipated challenges and how they may be able to successfully implement in their setting with the available resources. HCPs decided to implement FAST-M intervention in their setting and they also acknowledged the importance of a training program for HCPs to implement FAST-M bundle care tools in their setting. It was recognised

For peer review only - http://bmjopen.bmj.com/site/about/guidelines.xhtml

that the FAST-M protocol comprises similar practices but in an organized and structured way, and was well-regarded by all HCPs. They valued the implication of FAST-M intervention as stated:

"We are already doing these all things except documentation so it will be easy to apply. You know the guidelines, you have got an algorithm then it would be difficult to miss any patient. So it's a very good thing and this can be implemented. We have everything but there should be training and if you give that it would be easy to implement: (FGD- Associate Professor- Medicine)

Discussion

Our findings revealed several potential facilitators for the uptake of FAST-M intervention. Firstly, the HCPs had highly favorable perceptions regarding the use of FAST-M bundle care tools. The major advantage identified was illustration of colored codes in the MEOWs chart such as red and yellow flags that assists in categorization of patients according to severity of their symptoms. The early identification of patients with maternal sepsis through MEOWs chart facilitates timely management of patients using decision and treatment tools.

Evolving morbidity can be difficult to recognise in the obstetric population because of the normal changes in peripartum physiology [26]. Delays in recognition of patient deterioration and initiation of treatment lead to worse outcomes in maternal populations [26]. Early Warning Systems (EWS) have been used since 1999 in the general patient population to identify clinical deterioration [27], though the Maternal Early Obstetric Warning System (MEOWS) has been promoted with the aim to reduce maternal morbidity and mortality, and improve clinical

For peer review only - http://bmjopen.bmj.com/site/about/guidelines.xhtml

outcomes [28]. The FAST-M intervention comprises different components for the recognition and management of maternal sepsis (Supplemental file 4).

During the development of the FAST-M bundle through a modified Delphi process, oxygen saturation was mostly perceived as of reasonable importance. Though, the feasibility of implementing this element in low-resource settings limited its usefulness due to the non-availability of pulse oximeters at that time in many low-resource settings [9]. However, considering the outbreak of COVID-19 infection and the availability of pulse oximeters at the study site, it was recommended to include oxygen saturation in the MEOWs chart to determine patient's clinical condition. The inclusion of oxygen saturation in the MEOWs chart is considered important based on the existing sepsis management practices of the facility. Moreover, the element of oxygen saturation is a significant indicator in the identification of patients' clinical conditions. Therefore, the supplementary element of oxygen saturation has been added to the bundle care tools prior to its implementation (Supplemental file-6).

The MEOWS chart in the FAST-M intervention tracks physiological parameters and evolving morbidity and once a predetermined threshold reaches, it triggers evaluation by a healthcare provider [28]. The healthcare professional determines further evaluation, treatment, or intervention as necessary through the use of decision tool and treatment bundle [29]. The systematic approach for screening and management of maternal sepsis patients through the FAST-M intervention supports its implementation in the low-resource setting in Pakistan.

All HCPs acknowledged the FAST-M bundle care tools as easy to use as they do not require any invasive procedures to identify suspected maternal sepsis cases and trigger appropriate actions. Secondly, the HCPs deliberated about long-term improvement in patient's health outcomes

For peer review only - http://bmjopen.bmj.com/site/about/guidelines.xhtml

678 through the use of FAST-M intervention such as the decrease in length of patients' stay at the hospital, and improvement in maternal morbidities and mortalities overall.

680 Our study findings identified that the shortage of health care providers hindered many aspects of sepsis care delivery, and may be a critical barrier to any intervention. As the hospital provides free of charge care to patients, there is high influx of patients in the facility. This high volume of patients' increases workload on health care providers and eventually the shortage of healthcare workers is associated with adverse patient outcomes and comprised quality in patient care [30]. Therefore, all the study participants suggested involving nursing interns, trainee dispensers, and other available human resources to reduce doctors' and nurses' workload through shared responsibilities and employing a task-sharing approach. The approach of task sharing of specialists with trained non-specialist workers has provided positive outcomes in the improvement of patient care, reduced morbidity and mortality rates, and cost-effectiveness [30].

690 Accordingly, a training program has been planned as part of the implementation of the FAST-M intervention so all HCPs providers have the required knowledge to manage sepsis cases according to the FAST-M approach, making practice uniform across teams in the facility and ensuring the sustainability of FAST-M intervention as a long term benefit for patients.

694 The source identification denoted as 'S' in the FAST-M bundle requires a detailed history and examination to identify the infection source along with the targeted further investigations. The training program will provide an opportunity to improve this aspect, including the significance of taking a detailed history and examination and documenting them. This is very important to provide quality care and to help health care providers to plan a patient's treatment to maintain the continuum of care [31].

For peer review only - http://bmjopen.bmj.com/site/about/guidelines.xhtml

The FAST-M implementation in districts of Malawi provided useful example of effective implementation where champions played a significant role in implementing FAST-M intervention, and their contribution for intervention provided day-to-day oversight of healthcare practitioners' practice [10]. Our study findings suggest that the clinical practice variations among healthcare providers is a potential major hindering factor in implementation of FAST-M intervention, and yet we decided to select maternal sepsis champions. These champions could potentially standardise the practices for the management of maternal sepsis in all the departments managing such cases. To continue to strengthen the implementation of this intervention, champions will be selected during training program based on the consensus of healthcare providers involved in the training of FAST-M intervention.

Moreover, the HCPs were concerned about the compromised quality of available resources such as antibiotics and laboratory investigations which voiced their uncertainty to support FAST-M intervention. They felt that the hospital's environment and the quality of available resources did not support patients' clinical management. It was identified that the hospital system set for laboratory investigations is lengthy and time-consuming.

While the quality of health services within the clinical setting is imperative to provide effective care to the patients [32]. Study findings also suggest that the treatment cost adds to the financial burden of patients and leads to the discontinuation of medical treatment [33]. Thus, the practicability of intervention depends on the facility environment, availability of resources and its affordability for implementation and the readiness of 'healthcare administrators' who are accountable for provision of healthcare supplies. The role of healthcare administrators in upgrading the system is quite significant to avoid barriers to implementation. Hence, the healthcare administrators provided assurance for provision of supplies and resources as a stance

For peer review only - http://bmjopen.bmj.com/site/about/guidelines.xhtml

to reduce maternal sepsis rate at their healthcare setting and will be fully included in the implementation process, including the training and champion network.

Some specialists raised consideration of broadening the bundle to include more comprehensive sepsis care including consideration of steroid therapy and inotrope support. As part of the adaptation process, this issue was fully discussed with a range of local and international experts from the gynecology and intensive care fields and it was decided that these aspects would be most appropriate only for specialist doctors, normally in an ICU environment, so would not be suitable for inclusion in the first response bundle. However, the management of patients using steroids would be emphasized during the training program to delineate its role in the management of COVID-19 as a distinct situation from other bacterial causes of maternal sepsis to ensure rational and evidence based steroid use.

Antibiotics administration is one of the easily available, free of cost and important components of FAST-M treatment bundle for sepsis management. The FAST-M treatment bundle applied in the earlier study conducted in Malawi [10] was therefore of the important. We explored healthcare providers' views regarding use of antibiotics in their local setting for treatment of maternal sepsis. It was identified that Ceftriaxone is easily available free of cost to patients and it provides positive results in treatment of sepsis. Thus, it was agreed to use ceftriaxone as first choice of antibiotics in FAST-M treatment bundle. Moreover, it was also acknowledged that Piperacillin/tazobactam and meropenem are used for treatment of confirmed sepsis cases due to the current understanding of the organisms responsible for maternal sepsis and the antimicrobial resistance patterns. Though patients pay out of pocket for the cost of these antibiotics. Thus, Meropenem and Piperacillin/Tazobactam were proposed as the second choice of antibiotics due

For peer review only - http://bmjopen.bmj.com/site/about/guidelines.xhtml

to their availability and cost. The Malawian version of FAST-M treatment bundle was therefore modified for locally appropriate antibiotic guidelines (Supplemental file-6).

The importance of an explicit sepsis care policy was discovered during interviews and focus group discussion to assist in standardising infection regulations in the hospital. It was identified that the FAST-M intervention can serve as a guiding policy to provide evidence-based information to support clinical decision-making. Therefore, a unified system of FAST-M intervention for sepsis care in the facility for maternal patients can serve as a standard tool for maternal sepsis management.

The major strength of this study is the use of CFIR that guided the researchers' focus, starting with observations and documenting from a broad health systems and programme implementation perspective, becoming more specific in the later performed interviews and focus group discussion. Moreover, participation of HCPs from several levels to ask their feedback on the research question, and by interviewing HCPs about their experiences helped in gaining better insights about their practices and perceptions.

The study also has some limitations. First, the study focused only on the perspective of the healthcare providers who were involved in the management and treatment of maternal sepsis patients; therefore, the sample size was limited and important perspectives from patients and their families could have been missed. Secondly, the intervention would be implemented in only one study setting in Pakistan at this time. However, it is notable that this site serves a diverse population from the urban and rural areas of province of Sindh. The FAST-M tools were specifically adapted according to the existing sepsis practices of the current study setting. Future studies to explore feasibility of FAST-M bundle would require adaptation prior to implementing in other low-resource settings of Pakistan.

For peer review only - http://bmjopen.bmj.com/site/about/guidelines.xhtml

We believe that it is possible to implement the FAST-M intervention in low-resource settings of Pakistan and we recommend several strategies to address the challenges facilities may face in their local context. The hospital, leadership and HCPs require collaboration to work as a multidisciplinary team to advance sepsis management practices and understand its implications. This could be achieved through development and dissemination of FAST-M intervention as a sepsis management guideline in the facility.

The distribution of supportive resources to provide education to all HCPs including doctors, nurses and healthcare administrators about FAST-M tools is required to increase knowledge and awareness of FAST-M bundle. Also, facilities will require selected champions for implementation of the FAST-M intervention.

Overall, bundle care tools have the potential to enhance improvements in sepsis care. However, the implementation challenges posed by these bundles should be examined, especially in low-resource settings, where facilities and services have not yet flourished.

We identified facilitators and barriers for implementation of this intervention from only one of the facilities in Pakistan selected as our study site. Future research is needed to understand how implementation of this adapted FAST-M intervention works when implemented as part of care, and to rigorously evaluate its effectiveness and key implementation outcomes such as the sustainability of the intervention.

Conclusion

The FAST-M maternal sepsis bundle has the potential to be used as an integrated strategy for early recognition and management of maternal sepsis in low resource health settings in Pakistan.

For peer review only - http://bmjopen.bmj.com/site/about/guidelines.xhtml

We found several barriers and facilitators for its implementation and suggested key adaptations to the intervention which we perceive will help address these barriers.

Based on this formative research, the FAST-M tools and implementation approach in their adapted format will be implemented in the selected health facility and mixed-methods research conducted to assess the feasibility of implementing these adapted tools as part of the health care system in Pakistan.

Data availability statement

The datasets were collected and analyzed and can be made available from the corresponding author on reasonable request

Ethics statements

Patient consent for publication

Not required

Ethical approval

Ethical approval for this study was obtained from the LUMHS hospital [REC/-886, 4-87], Aga Khan University Ethical Review Committee [2019-2061-7102] and National Bioethics Committee [515/20/]. Participants will be asked to provide written consent to indicate their willingness to participate. Voluntary participation and the right to ask any questions and to decline participation at any time will be emphasized during the data collection.

For peer review only - http://bmjopen.bmj.com/site/about/guidelines.xhtml

Funding

This research was commissioned by the National Institute for Health Research (NIHR). Award Ref: NIHR300808 Host: University of Liverpool. The views expressed in this paper are those of the authors and not necessarily those of the NIHR or the Department of Health and Social Care. The project was also supported by the University of Birmingham Alumni funds.

Acknowledgements

We would also like to acknowledge Dr. Sadia Shakoor (Associate Professor Section of Microbiology, Department of Pathology and Laboratory Medicine, Aga Khan University Hospital) in providing information and recent statistics related to use of antibiotics in Pakistan. We would like to acknowledge health officials and individuals: Dr. Anna Blennerhassett (for her expert contributions to the project), Dr. Zulfiqar Shah, Dr. Madiha Shah, Dr. Imran Karim Shaikh, Dr. Mumtaz Lakho, Dr. Fouzia Sheikh, Mr. Sattar Jatoi, Dr. Nargis and Dr. Sabrina (for providing assistance, contact numbers and permissions for data collection at field sites).

For peer review only - http://bmjopen.bmj.com/site/about/guidelines.xhtml

References

1. Say L, Chou D, Gemmill A, Tunçalp Ö, Moller AB, Daniels J, Gülmezoglu AM, Temmerman M, Alkema L. Global causes of maternal death: a WHO systematic analysis. The Lancet Global Health. 2014 Jun 1;2(6):e323-33.
2. Bonet M, Pileggi VN, Rijken MJ, Coomarasamy A, Lissauer D, Souza JP, Gülmezoglu AM. Towards a consensus definition of maternal sepsis: results of a systematic review and expert consultation. Reproductive health. 2017 Dec;14(1):67.
3. World Health Organization, Unicef. Trends in maternal mortality: 1990 to 2013: estimates by WHO, UNICEF, UNFPA, The World Bank and the United Nations Population Division: executive summary. World Health Organization; 2014.
4. van Dillen J, Zwart J, Schutte J, van Roosmalen J. Maternal sepsis: epidemiology, etiology and outcome. Current opinion in infectious diseases. 2010 Jun 1;23(3):249-54.
5. Khalid S Khan, Daniel Wojdyla, Lale Say, A Metin Gülmezoglu, Paul FA Van Look,WHO analysis of causes of maternal death: a systematic review,The Lancet,Volume 367, Issue 9516,2006,Pages 1066-1074,ISSN 0140-6736,https://doi.org/10.1016/S0140-6736(06)68397 (https://www.sciencedirect.com/science/article/pii/S0140673606683979)
6. Lissauer D, Cheshire J, Dunlop C, Taki F, Wilson A, Smith JM, Daniels R, Kissoon N, Malata A, Chirwa T, Lwesha VM. Development of the FAST-M maternal sepsis bundle for use in low-resource settings: a modified Delphi process. BJOG: An International Journal of Obstetrics & Gynaecology. 2020 Feb;127(3):416-23.

For peer review only - http://bmjopen.bmj.com/site/about/guidelines.xhtml

850 7. Seymour CW, Gesten F, Prescott HC, Friedrich ME, Iwashyna TJ, Phillips GS,
851 Lemeshow S, Osborn T, Terry KM, Levy MM. Time to treatment and mortality during
852 mandated emergency care for sepsis. New England Journal of Medicine. 2017 Jun
853 8;376(23):2235-44.

854 8. Taki F. *The development of a care bundle to improve the initial management of maternal*
855 *sepsis for use in low and lower-middle income countries* (Doctoral dissertation,
856 University of Birmingham).

857 9. Lissauer D, Cheshire J, Dunlop C, Taki F, Wilson A, Smith JM, Daniels R, Kissoon N,
858 Malata A, Chirwa T, Lwesha VM. Development of the FAST-M maternal sepsis bundle
859 for use in low-resource settings: a modified Delphi process. BJOG: An International
860 Journal of Obstetrics & Gynaecology. 2020 Feb;127(3):416-23.

861 10. Cheshire J, Jones L, Munthali L, Kamphinga C, Liyaya H, Phiri T, Parry-Smith W,
862 Dunlop C, Makwenda C, Devall AJ, Tobias A. The FAST-M complex intervention for
863 the detection and management of maternal sepsis in low-resource settings: a multi-site
864 evaluation. BJOG: An International Journal of Obstetrics & Gynaecology. 2021 Feb 4.

865 11. Madhudas C, Khurshid F, Sirichand P. Maternal morbidity and mortality associated with
866 puerperal sepsis. Journal of Liaquat University of Medical and Health Sciences. 2011 Sep
867 1;10(03):121.

868 12. Iftikhar R. A study of maternal mortality. J Surg Pak.(Int.). 2009 Oct;14(4):176-8.

869 13. Arulkumaran N, Singer M. Puerperal sepsis. Best Practice & Research Clinical Obstetrics
870 & Gynaecology. 2013 Dec 1;27(6):893-902.

For peer review only - http://bmjopen.bmj.com/site/about/guidelines.xhtml

14. Shamshad S, Shamsher S, Rauf B. Puerperal sepsis—still a major threat for parturient. Journal of Ayub Medical College Abbottabad. 2010 Sep 1;22(3):18-22.

15. Begum S, Aziz-un-Nisa BI. Analysis of maternal mortality in a tertiary care hospital to determine causes and preventable factors. J Ayub Med Coll Abbottabad. 2003;15(2):49-52.

16. Reinhart K, Kissoon N. Sepsis Guidelines for Pakistan. Anaesth Pain & intensive Care. 2015 Apr 1;19(2):105-7.

17. Kurji Z, Premani ZS, Mithani Y. Analysis of the health care system of Pakistan: lessons learnt and way forward. J Ayub Med Coll Abbottabad. 2016;28(3):601.

18. Agha S, Fitzgerald L, Fareed A, Rajbhandari P, Rahim S, Shahid F, Williams E, Javed W, Currie S. Quality of labor and birth care in Sindh Province, Pakistan: findings from direct observations at health facilities. Plos one. 2019 Oct 17;14(10):e0223701.

19. Ahmed, S.I., Sikandar, R., Barolia, R. *et al.* Evaluation of the feasibility of the FAST-M maternal sepsis intervention in Pakistan: a protocol. *Pilot Feasibility Stud* **8,** 130 (2022). https://doi.org/10.1186/s40814-022-01090-4

20. Peters DH, Adam T, Alonge O, Agyepong IA, Tran N. Implementation research: what it is and how to do it. Bmj. 2013 Nov 20;347.

21. Damschroder L, Hall C, Gillon L, Reardon C, Kelley C, Sparks J, Lowery J. The Consolidated Framework for Implementation Research (CFIR): progress to date, tools and resources, and plans for the future. InImplementation science 2015 Dec (Vol. 10, No. 1, pp. 1-1). BioMed Central.

For peer review only - http://bmjopen.bmj.com/site/about/guidelines.xhtml

22. Birken SA, Powell BJ, Presseau J, Kirk MA, Lorencatto F, Gould NJ, Shea CM, Weiner BJ, Francis JJ, Yu Y, Haines E. Combined use of the Consolidated Framework for Implementation Research (CFIR) and the Theoretical Domains Framework (TDF): a systematic review. Implementation science. 2017 Dec;12(1):1-4.

23. Hennink MM, Kaiser BN. Saturation in qualitative research. SAGE Publications Limited; 2020.

24. Abdalla MM, Oliveira LG, Azevedo CE, Gonzalez RK. Quality in qualitative organizational research: Types of triangulation as a methodological alternative. Administração: ensino e pesquisa. 2018;19(1):66-98.

25. Royal College of Obstetricians and Gynaecologists. Bacterial Sepsis in Pregnancy. Green-top Guideline No.64a. London: RCOG; 2012 [https:// www.rcog.org.uk/en/guidelines-research-services/guidelines/gtg64a/].

26. Edwards W, Dore S, van Schalkwyk J, Armson BA. Prioritizing maternal sepsis: national adoption of an obstetric early warning system to prevent morbidity and mortality. Journal of Obstetrics and Gynaecology Canada. 2020 May 1;42(5):640-3.

27. Arnolds DE, Carey KA, Braginsky L, Holt R, Edelson DP, Scavone BM, Churpek M. Comparison of early warning scores for predicting clinical deterioration and infection in obstetric patients. BMC pregnancy and childbirth. 2022 Dec;22(1):1-9.

28. Ryan HM, Jones MA, Payne BA, Sharma S, Hutfield AM, Lee T, Ukah UV, Walley KR, Magee LA, von Dadelszen P. Validating the performance of the modified early obstetric warning system multivariable model to predict maternal intensive care unit admission. Journal of Obstetrics and Gynaecology Canada. 2017 Sep 1;39(9):728-33.

For peer review only - http://bmjopen.bmj.com/site/about/guidelines.xhtml

29. Smith MB, Chiovaro JC, O'Neil M, Kansagara D, Quiñones AR, Freeman M, Motu'apuaka ML, Slatore CG. Early warning system scores for clinical deterioration in hospitalized patients: a systematic review. Annals of the American Thoracic Society. 2014 Nov;11(9):1454-65.

30. Padmanathan P, De Silva MJ. The acceptability and feasibility of task-sharing for mental healthcare in low and middle income countries: a systematic review. Social science & medicine. 2013 Nov 1;97:82-6.

31. Dhar RL. Service quality and the training of employees: The mediating role of organizational commitment. Tourism Management. 2015 Feb 1;46:419-30

32. Sajid MS, Baig MK. Quality of health care: an absolute necessity for public satisfaction. International Journal of Health Care Quality Assurance. 2007 Sep 11.

33. Shaikh I, Singh S. On the examination of out-of-pocket health expenditures in India, Pakistan, Sri Lanka, Maldives, Bhutan, Bangladesh and Nepal. Business: Theory and Practice. 2017 Mar 5;18:25.

For peer review only - http://bmjopen.bmj.com/site/about/guidelines.xhtml

Footnotes

Authors' contributions

SI, DL, RB & LS conceptualized the design of the study and creation of data collection tools. RS, RR, SK assisted in data collection from field site. SI, RB, BK & GK managed data collection and interpretation. SI and BK carried out the analysis and wrote the initial manuscript. All authors provided input during the interpretation of the data and revising of the manuscript. DL, AC, RB, JS, CD provided feedback on the first draft. SI & BK edited and wrote the final draft. The authors read and approved the final manuscript.

Competing interests

The authors declare that they have no competing interests.

view only

COREQ (COnsolidated criteria for REporting Qualitative research) Checklist

A checklist of items that should be included in reports of qualitative research. You must report the page number in your manuscript where you consider each of the items listed in this checklist. If you have not included this information, either revise your manuscript accordingly before submitting or note N/A.

Topic	Item No.	Guide Questions/Description	Reported on Page No.
**Domain 1: Research team and reflexivity**			
Personal characteristics			
Interviewer/facilitator	1	Which author/s conducted the interview or focus group?	
Credentials	2	What were the researcher's credentials? E.g. PhD, MD	
Occupation	3	What was their occupation at the time of the study?	
Gender	4	Was the researcher male or female?	
Experience and training	5	What experience or training did the researcher have?	
Relationship with participants			
Relationship established	6	Was a relationship established prior to study commencement?	
Participant knowledge of the interviewer	7	What did the participants know about the researcher? e.g. personal goals, reasons for doing the research	
Interviewer characteristics	8	What characteristics were reported about the inter viewer/facilitator? e.g. Bias, assumptions, reasons and interests in the research topic	
**Domain 2: Study design**			
Theoretical framework			
Methodological orientation and Theory	9	What methodological orientation was stated to underpin the study? e.g. grounded theory, discourse analysis, ethnography, phenomenology, content analysis	
Participant selection			
Sampling	10	How were participants selected? e.g. purposive, convenience, consecutive, snowball	
Method of approach	11	How were participants approached? e.g. face-to-face, telephone, mail, email	
Sample size	12	How many participants were in the study?	
Non-participation	13	How many people refused to participate or dropped out? Reasons?	
Setting			
Setting of data collection	14	Where was the data collected? e.g. home, clinic, workplace	
Presence of non-participants	15	Was anyone else present besides the participants and researchers?	
Description of sample	16	What are the important characteristics of the sample? e.g. demographic data, date	
Data collection			
Interview guide	17	Were questions, prompts, guides provided by the authors? Was it pilot tested?	
Repeat interviews	18	Were repeat inter views carried out? If yes, how many?	
Audio/visual recording	19	Did the research use audio or visual recording to collect the data?	
Field notes	20	Were field notes made during and/or after the inter view or focus group?	
Duration	21	What was the duration of the inter views or focus group?	
Data saturation	22	Was data saturation discussed?	
Transcripts returned	23	Were transcripts returned to participants for comment and/or	

For peer review only

Topic	Item No.	Guide Questions/Description	Reported on Page No.
		correction?	
**Domain 3: analysis and findings**			
Data analysis			
Number of data coders	24	How many data coders coded the data?	
Description of the coding tree	25	Did authors provide a description of the coding tree?	
Derivation of themes	26	Were themes identified in advance or derived from the data?	
Software	27	What software, if applicable, was used to manage the data?	
Participant checking	28	Did participants provide feedback on the findings?	
Reporting			
Quotations presented	29	Were participant quotations presented to illustrate the themes/findings? Was each quotation identified? e.g. participant number	
Data and findings consistent	30	Was there consistency between the data presented and the findings?	
Clarity of major themes	31	Were major themes clearly presented in the findings?	
Clarity of minor themes	32	Is there a description of diverse cases or discussion of minor themes?	

Developed from: Tong A, Sainsbury P, Craig J. Consolidated criteria for reporting qualitative research (COREQ): a 32-item checklist for interviews and focus groups. *International Journal for Quality in Health Care*. 2007. Volume 19, Number 6: pp. 349 – 357

Once you have completed this checklist, please save a copy and upload it as part of your submission. DO NOT include this checklist as part of the main manuscript document. It must be uploaded as a separate file.

For peer review only

Supplementary file 2

Informed Consent

Title of study:	Extension of the FAST-M maternal sepsis bundle in Pakistan, a feasibility study
Chief Investigator:	Professor David Lissauer
Site:	Liaquat University of Health Sciences Pakistan
Site Principal Investigator:	Dr Sheikh Irfan Ahmed
Site CO-PI's	Dr Lumaan Sheikh, Dr Raheel Sikandar and Dr. Rubina Barolia
Ethics approval:	AKU ERC-2019-2061-7102,<br>LUMHS/ REC/-886, 4-87/NBC-515/20/
Affiliated organizations:	University of Birmingham, University of Liverpool & Aga Khan University Hospital Pakistan & Liaquat University of Medical & Health Science, Jamshoro.

We would like to invite you to take part in this research study. Before you decide, we would like you to understand the study, why the research is being done and what this part of the study involves for you. One of the team will explain the study to you and answer any questions you may have.

Part 1: Purpose of the study

What is the purpose of the overall study?

We are developing an intervention that we hope will improve the care of patients with maternal sepsis around the world. Sepsis is when an infection has become severe enough to lead to organ dysfunction and become life threatening.

The intervention is composed of three things:

1. The MEOWS (Maternal Early Warning Scores) chart tool to help you monitor patient's observations and help detect maternal sepsis

2. The FAST-M sepsis "bundle", to help ensure fast, consistent and effective treatment of maternal sepsis

3. A training day to learn to use the tools to help recognize and treat maternal sepsis

For peer review only

We hope that this intervention will make caring for patients with maternal sepsis easier. This study aims to discover whether it is possible to introduce this intervention into Pakistan healthcare facilities.

We hope to try and understand the good and bad aspects of the bundle to try and make it more user friendly and effective. We hope that using this bundle will make caring for patients with maternal sepsis easier.

In order to achieve this we hope to:

1. Understand your current experiences in managing maternal sepsis at your hospital

2. Understand what you thought was good and bad about the intervention.

3. Understand ways to improve the intervention.

4. Evaluate the intervention to see if it improves care in your hospital.

We hope you will be willing to participate in all of the activities for the study mentioned above.

Why have I been invited to participate?

You have been invited to participate because you work in maternity care and we would like to understand your experiences of maternal sepsis and the proposed intervention.

What will I have to do if I take part?

You will be interviewed several times over a period of six to eight months. Sometimes these will be one on one interviews and sometimes in groups. The interviews will be in English and take up to an hour. The interview will take place at or close-by to your place of work, at a time that is convenient to you. The interview will be audio-recorded to allow us to analyse the information you give us. Some or all of the information will be transcribed word for word. This information will be used in several ways – all of which will be anonymous so that your identity is not disclosed. The table describes how your information will be used.

At the start of the study the information that you give us will be used to understand current practice at your hospital for the management of maternal sepsis. During the study the information that you give us will be used to discover the good and bad aspects of the intervention and how it could be improved to make it easier for you to manage patients with maternal sepsis. This will help us decide whether the intervention is a success or not. Some of the information you give us, including word for word extracts, will be used in the final project report, which may also be published in a journal.

Do I have to take part?

It is completely up to you to volunteer to be interviewed and it will have no effect upon your work. We will describe the study and go through this information sheet with you. If you decide to take part, we will then ask you to sign a consent form.

What are the possible disadvantages and risks of taking part?

For peer review only

Before participating you should consider that we will be asking you about your experiences, opinions, beliefs and feelings in relation to the intervention. We are interested in finding out about the positive things that help you do your work and anything that hinders your work. Although unlikely, there is a possibility that you might feel upset when answering these questions during the interview. If this was to occur, you would be able to take a break or continue another day.

There will be an opportunity at the end of the interview for you to consider whether there is anything that you have discussed that you would prefer not to be included in the transcript. The transcript will also be made available to you to review by email if you would like. As a participant you are free to withdraw during the interview and up to a month afterwards, without giving a reason.

What are the possible benefits of taking part?

We hope that you will find the experience interesting and enjoyable. The information we collect from this study will be used to help us make the intervention the best it can be. Your interview will also be very important in evaluating the interventions effects at your hospital and its potential usefulness in the management of maternal sepsis.

What are the financial considerations of taking part in this study?

We would like to provide you a token of thanks at the end of the interview for providing your time and information with us.

What if there is a problem?

Any complaint about the way you have been dealt with during the study or any possible difficulty you might suffer will be addressed. Information on this is given in Part 2.

Will my taking part in the study be kept confidential?

We will follow ethical practice and all information about you will be handled in confidence. Further details are included in Part 2.

This completes part 1. If the information in Part 1 has interested you and you are considering participation, please read the additional information in Part 2 before making any decision.

Part 2: Conduct of the study

What will happen if I don’t want to carry on with the study?

You may withdraw from the study without giving a reason. If you chose to withdraw from the study during or up to one month after your interview, we might ask you whether we can use the information you have given us, such as your interview answers. If you don’t want to carry on with the study but you give us permission to use the information already collected, we will proceed to keep it securely. If you wish to withdraw and don’t want your data to be used for the study, we will delete any recordings and destroy transcript files.

What if there is a problem?

For peer review only

If you have a concern about any aspect of this study, you can speak to the researchers, who will do their best to answer your questions. Their contact details are on the last page.

Will my taking part in this study be kept confidential?

The study will take place at your workplace, and for this reason it is possible that other work colleagues will be aware of your participation. However, we will follow these procedures for collecting, storing, processing and destroying information about you to ensure your confidentiality and safeguard your data:

- The recording of any information you give us during your interview will be stored in a password protected file and only authorised people will have access to it. This will help prevent people identifying your voice.
- The data transcribed from recordings will be stored securely on a computer with access restricted by a password. Transcripts will not include names or locations. Consent forms and printed transcripts will be kept in a locked cabinet, only accessible to authorised researchers.
- Data collected will be used for this study but, with your permission, might also be retained to include it anonymously in future studies.
- The identifiable data will be retained for the duration of the study and will be disposed of securely (i.e. shredding documents).

As a participant, you would have the right to check the accuracy of data held about you and correct any errors.

What will happen to the results of the research study?

The researchers will write a report outlining the results of this study. You will not be identified in any report, presentation or publication, however extracts from your interviews may be reproduced. The results will be used to inform local practice and a future possible larger scale trial of the intervention. If you are interested in the outcome of the research, then a summary of the findings can be sent to you via email and if you wish you will be invited to attend a feedback day at the end of the project.

Who is organizing the research

This study is being carried out by the University of Birmingham, UK. University of Liverpool, UK and Aga Khan University Hospital(AKUH), Pakistan The research team is being led by Dr David Lissauer, Dr Lumaan Sheikh and Dr Sheikh Irfan is the researcher conducting this part of the study.

Who has reviewed the study?

This study has been reviewed by the National Bioethics Committee Pakistan and College Research Ethics Committee in AKUH.

Contact details:

For peer review only

Dr Sheikh Irfan Ahmed, Senior Instructor, AKUH National stadium road, Karachi Email: sheikh.irfan@aku.edu Telephone number: +92-021-34864650

Dr David Lissauer Lecturer in Maternal and Fetal Medicine, University of Birmingham, UK Email: David.Lissauer@liverpool.ac.uk

Dr Lumaan Sheikh Associate Professor, AKUH National stadium road, Karachi Email: lumaan.sheikh@aku.edu Telephone number: +92-021-34864641

Dr Raheel Sikandar Professor, Liaquat University of Medical & Health Sciences, Jamshoro Email: pgmc@lumhs.edu.pk Telephone number: + 92-22-9213322

Please keep this information sheet for your own records.

Dr Rubina Barolia, Associate Professor and Assistant Dean, School of Nursing, AKU, Email: rubina.barolia@aku.edu Telephone number: +92-021-34865446

Bakhtawar Khowaja, Research Coordinator, AKUH National stadium road, Karachi Email: Bakhtawar.hanif@aku.edu Telephone number: +92-021-34864626

PLEASE INITIAL THE BOXES IF YOU AGREE WITH EACH SECTION:

1. I have read the information sheet version 2.5 for the above study and have been given a copy to keep. I have had the opportunity to consider the information, ask questions and have had these answered satisfactorily. ☐
2. I understand that my participation is voluntary and that I am free to withdraw up to one month after my participation without giving any reason. ☐
3. I agree to be interviewed for research in this study. I agree to my interview being audio-recorded and I understand that transcripts will be anonymised. I understand that participating in the interview for this research is voluntary and that I am free to withdraw my approval for use of the audio recordings and transcripts up to one month after my participation. ☐
4. I understand that anonymised sections of data collected during the study, may be looked at by individuals from regulatory authorities in the UK or Pakistan. I give permission for these individuals to have access to my anonymised transcript. ☐
5. I understand that the researchers might publish an article in a journal with the results of this study. I give permission for my transcripts to be used for this purpose. I understand that these transcripts will be anonymised. ☐
6. I know how to contact the research team if I need to. ☐
7. I understand that I may terminate the interview at any time ☐
8. I am happy for information about me related to the study being stored on a password protected computer system, which will be backed-up in a separate location to keep this

For peer review only

information safe. Data collected will be used for this study but, might also be retained to include it anonymously in future studies ☐

☐

9. I agree to participate in this study.

SIGNATURES:

Participant Name and Surname ____________________ Date________________

Signature ____________________________

Researcher Name and Surname ___________________________ Date ____________________

Signature________________________________

For peer review only

Supplementary file 1

Interview Guide

Intervention Characteristics

1. What do you know about the intervention or its implementation?
2. How different is this intervention from your existing practices?
3. What kind of information or evidence are you aware of that shows whether or not the intervention will work in your setting?
4. What kinds of changes or alterations do you think you will need to make to the intervention so it will work effectively in your setting?
    - Do you think you will be able to make these changes? Why or why not?
5. What is your perception of the bundling of the intervention for implementation and quality of the supporting materials? Prompts: format, design, user-friendly. Duration, scope, intricacy and number of steps

Outer Setting

6. How do you think the individuals served by your organization will respond to the intervention?
7. What barriers will the individuals served by your organization face to participating in the intervention?
8. What kind of local, state, or national performance measures, policies, regulations, or guidelines might be important in influencing how this intervention can be implemented?

Inner Setting

9. Can you describe how the intervention will be integrated into current processes?
10. What are your current guidelines to assess and manage patients with maternal sepsis? Probes: tool, framework or guidelines for maternal sepsis, lactate test
11. What is your knowledge about importance of lactate test and what is your current practice about lactate testing? Probes: implications for lactate test, guidelines for lactate test
12. What is your current patient to doctor and patient to nurse's ratio in your setting?

13. Explain the role of doctors and nurses in management of maternal sepsis in your organization. Which cadre is responsible for care and at what level of care? Probes: nurses, doctors, technicians and other health care cadres
14. Other than human resources, what resources are utilized in management of maternal sepsis in your hospital?
15. Do you expect to have sufficient resources to implement and administer the intervention?
    - [If no] What resources will not be available? Probes: human resource, equipments, critical units etc
16. Do you feel the training planned for you will prepare you to carry out the roles and responsibilities expected of you?
    - What are the positive aspects of planned training? What is missing?

Characteristics of Individuals

17. How do you feel about the intervention being used in your setting?
18. Do you think the intervention will be effective in your setting? Why or why not?

Process

19. Who will lead implementation of the intervention?
20. Are there people in your organization who are likely to champion (go above and beyond what might be expected) the intervention?
    Prompts: Position of these champions have in your organization?
21. How do you think they will help with implementation?

MODIFIED EARLY OBSTETRIC WARNING CHART (MEOWS CHART)

FAST-M

Contact clinical decision maker if patient triggers **ONE RED** or **TWO YELLOW** flags at any one time.

Patient: | Patient ID: | DOB/Age:

WRITE **VALUES** IN BOXES PROVIDED

		Date															
		Time															
		Initials															
Respiratory rate (breaths per minute)	25 or more	RED															
	21 - 24	YELLOW															
	11 - 20	NORMAL															
	10 or less	RED															
Temperature (°C)	38 or more	YELLOW															
	36.0 to 37.9	NORMAL															
	35.9 or less	YELLOW															
Heart rate (beats per minute)	120 or more	RED															
	100 - 119	YELLOW															
	50 - 99	NORMAL															
	40 - 49	YELLOW															
	39 or less	RED															
Systolic blood pressure (mmHg)	160 or more	RED															
	140 -159	YELLOW															
	100 -139	NORMAL															
	90 - 99	YELLOW															
	89 or less	RED															
Diastolic blood pressure (mmHg)	110 or more	RED															
	90 -109	YELLOW															
	40 - 89	NORMAL															
	39 or less	RED															
Urine output Hours since patient passed urine (tick box)	12 hours or less	NORMAL															
	12 - 18 hours	YELLOW															
	18 hours or more OR less than 0.5 ml/kg/hour	RED															
Mental State (tick box)	Alert	NORMAL															
	Not Alert	RED															
Looks unwell (tick box)	No	NORMAL															
	Yes	YELLOW															
TOTAL YELLOW FLAGS																	
TOTAL RED FLAGS																	
ACTION TAKEN (IF REQUIRED) Yes (Y) / No (N)																	

ACT NOW if patient triggers ONE RED or TWO YELLOW flags at any time. Escalate to clinical decision maker and start FAST-M decision tool.

UNITED AGAINST MATERNAL SEPSIS

THINK SEPSIS, ACT FAST-M

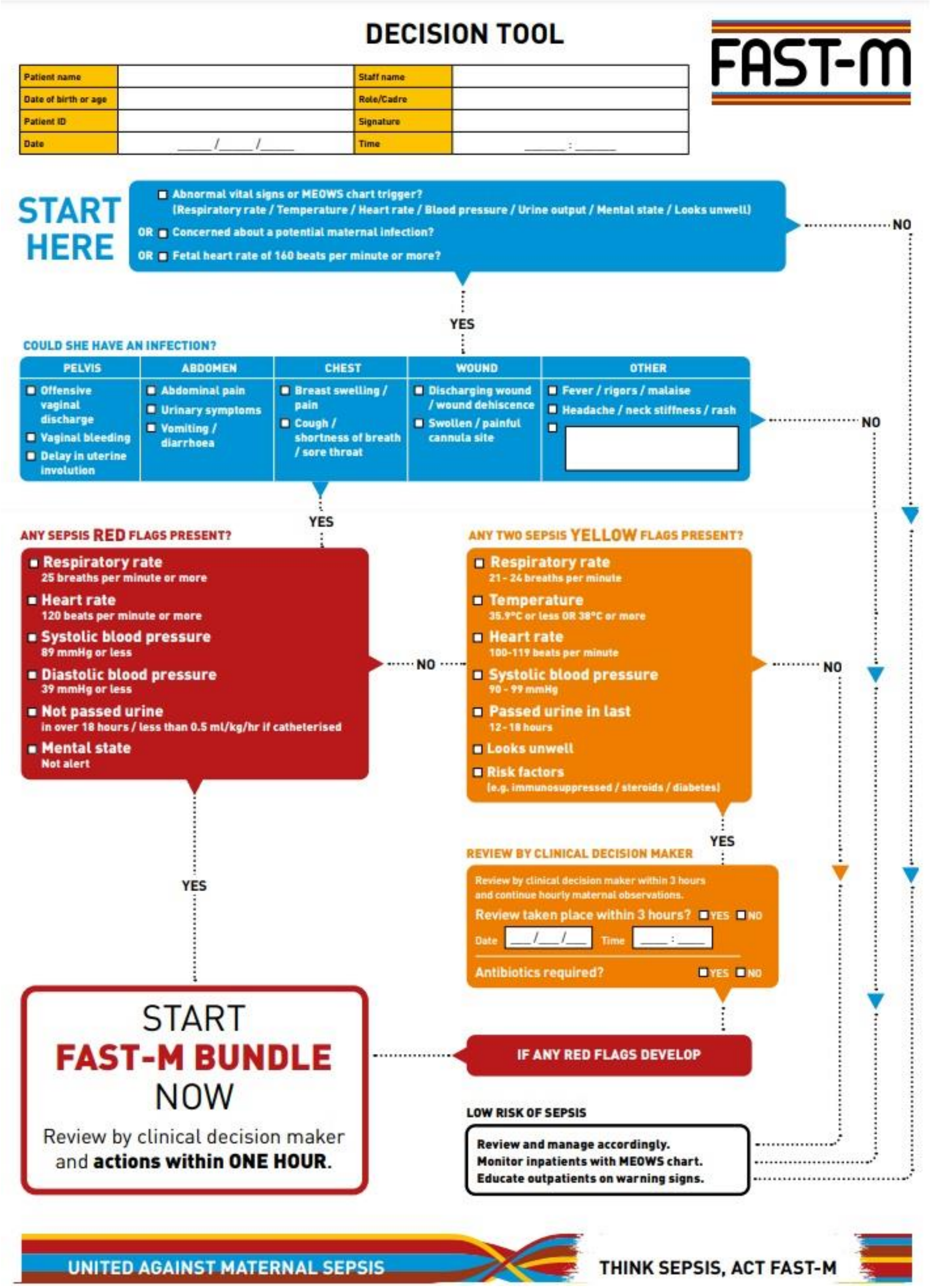
DECISION TOOL
FAST-M
Patient name
Date of birth or age
Patient ID
Date
Staff name
Role/Cadre
Signature
Time
START HERE
Abnormal vital signs or MEOWS chart trigger?
(Respiratory rate / Temperature / Heart rate / Blood pressure / Urine output / Mental state / Looks unwell)
OR Concerned about a potential maternal infection?
OR Fetal heart rate of 160 beats per minute or more?
NO
YES
COULD SHE HAVE AN INFECTION?
PELVIS
Offensive vaginal discharge
Vaginal bleeding
Delay in uterine involution
ABDOMEN
Abdominal pain
Urinary symptoms
Vomiting / diarrhoea
CHEST
Breast swelling / pain
Cough / shortness of breath / sore throat
WOUND
Discharging wound / wound dehiscence
Swollen / painful cannula site
OTHER
Fever / rigors / malaise
Headache / neck stiffness / rash
NO
YES
ANY SEPSIS RED FLAGS PRESENT?
Respiratory rate
25 breaths per minute or more
Heart rate
120 beats per minute or more
Systolic blood pressure
89 mmHg or less
Diastolic blood pressure
39 mmHg or less
Not passed urine
in over 18 hours / less than 0.5 ml/kg/hr if catheterised
Mental state
Not alert
NO
ANY TWO SEPSIS YELLOW FLAGS PRESENT?
Respiratory rate
21 - 24 breaths per minute
Temperature
35.9°C or less OR 38°C or more
Heart rate
100-119 beats per minute
Systolic blood pressure
90 - 99 mmHg
Passed urine in last
12 - 18 hours
Looks unwell
Risk factors
(e.g. immunosuppressed / steroids / diabetes)
NO
YES
YES
REVIEW BY CLINICAL DECISION MAKER
Review by clinical decision maker within 3 hours and continue hourly maternal observations.
Review taken place within 3 hours? YES NO
Date Time
Antibiotics required? YES NO
START
FAST-M BUNDLE
NOW
Review by clinical decision maker and actions within ONE HOUR.
IF ANY RED FLAGS DEVELOP
LOW RISK OF SEPSIS
Review and manage accordingly.
Monitor inpatients with MEOWS chart.
Educate outpatients on warning signs.
UNITED AGAINST MATERNAL SEPSIS
THINK SEPSIS, ACT FAST-M

TREATMENT BUNDLE

FAST-M

Patient name		Staff name	
D.O.B or age		Role/Cadre	
Patient ID		Signature	

Date & time of red flag observation	__/__/__ __:__	Date & time bundle started	__/__/__ __:__	Date & time of review by clinical decision maker	__/__/__ __:__

REMEMBER TO COMPLETE THESE ACTIONS WITHIN ONE HOUR

1HR

F — FLUIDS (caution in pre-eclampsia, severe anaemia and pulmonary oedema)

Date	__/__/__	Time fluids initiated	__:__	Initials		Give 500 ml crystalloid immediately. Repeat 500 ml boluses to a maximum of 30 ml/kg if hypotension persists.
Details / reason not completed						

A — ANTIBIOTICS

Date	__/__/__	Time started	__:__	Initials		See antibiotic guidelines below
Details / reason not completed						

S — SOURCE – identify and treat the source of infection

Date	__/__/__	Time considered	__:__	Initials		See source identification and treatment boxes below
Details / reason not completed						

T — TRANSPORT (to higher level hospital or location within hospital, if required)

Date & time transport considered	__/__/__ __:__	Initials		Transport Required	☐ YES ☐ NO
Date & time transport requested	__/__/__ __:__	Initials		☐ N/A	
Date & time patient left facility	__/__/__ __:__	Initials			
Destination					
Reason for any delay					

M — MONITORING (start MEOWS chart if not already started. Repeat observations every 30 minutes until otherwise decided by clinical decision maker)

Date & time monitoring commenced	__/__/__ __:__		Details / reason not completed
Maternal / fetal monitoring should include	• Respiratory rate<br>• Temperature<br>• Heart rate<br>• Blood pressure	• Urine output<br>• Mental state<br>• Fetal heart rate	
Neonatal monitoring and review commenced	☐ YES ☐ NO ☐ N/A		

ANTIBIOTIC GUIDELINES

Insert local guidance here

Immediate treatment for Maternal Sepsis:

- Ceftriaxone 2 g IV once daily (if no IV access this can be given as 2 IM injections of 1 g in different sites).
- If possible intra-abdominal source add Metronidazole 500 mg IV three times daily or 400 mg PO three times daily.

If above antibiotic regime is not available then give:

- Chloramphenicol 1 g IV/IM four times daily plus Gentamycin 240 mg IV/IM once daily.

If maternal infection source is known, or as soon as it is identified:

- Use specific treatment based on Malawi Standard Treatment Guidelines.

IDENTIFY THE SOURCE

Consider

- Clinical history
- Clinical examination
- Blood tests (if available) (FBC, U&Es, LFTs, CRP, clotting)
- Blood cultures
- HIV and Malaria testing
- Urine sample
- Swabs (wound, vagina, throat)
- Sputum sample
- Imaging (abdominal, chest)
- Lumbar puncture
- Other __________

REMOVE / TREAT THE SOURCE

Consider

- Malaria treatment
- Consider delivery of baby
- Removal of retained products of conception
- Debridement of wound / drainage of collection
- Removal of infected cannula / line
- Hysterectomy
- Targeted antibiotics once source known

UNITED AGAINST MATERNAL SEPSIS

THINK SEPSIS, ACT FAST-M

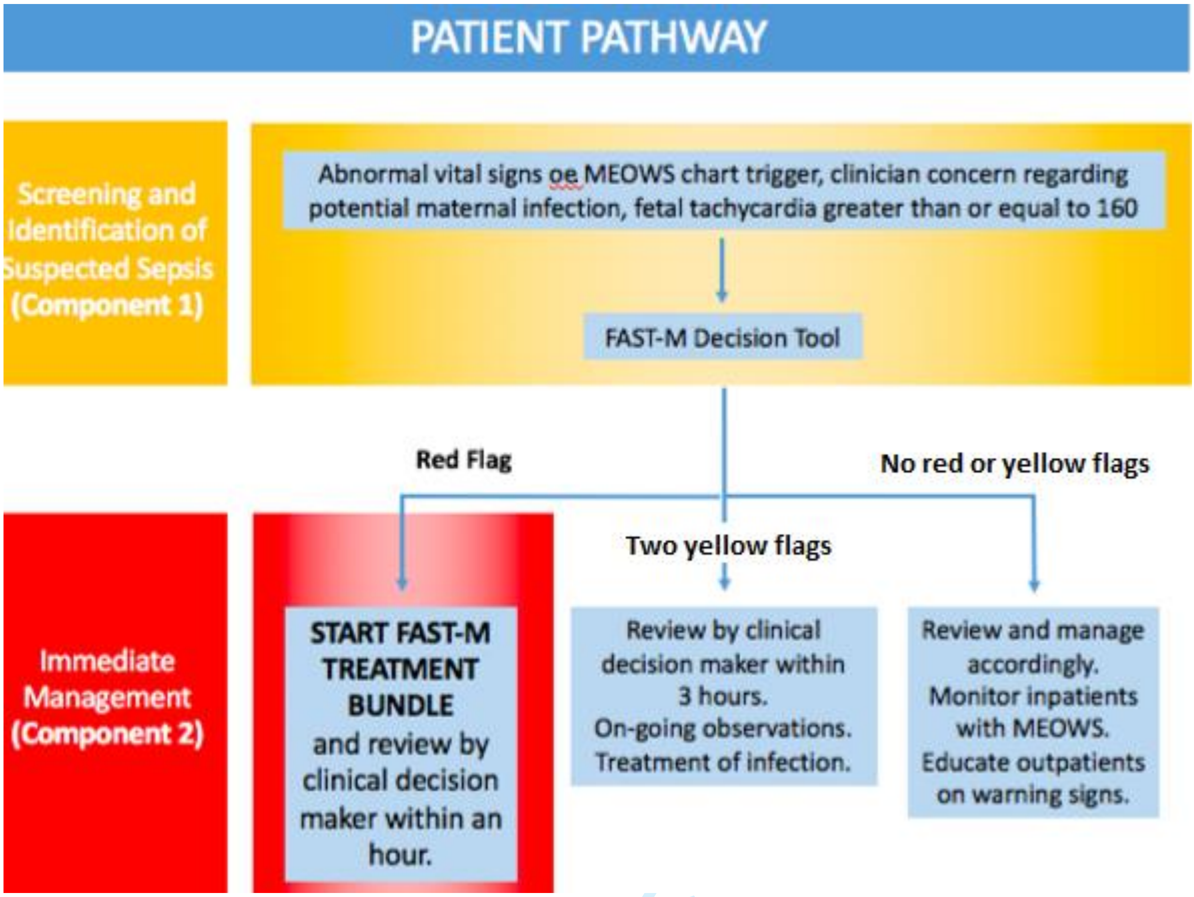
PATIENT PATHWAY
Screening and Identification of Suspected Sepsis (Component 1)
Abnormal vital signs oe MEOWS chart trigger, clinician concern regarding potential maternal infection, fetal tachycardia greater than or equal to 160
FAST-M Decision Tool
Red Flag
No red or yellow flags
Two yellow flags
Immediate Management (Component 2)
START FAST-M TREATMENT BUNDLE and review by clinical decision maker within an hour.
Review by clinical decision maker within 3 hours. On-going observations. Treatment of infection.
Review and manage accordingly. Monitor inpatients with MEOWS. Educate outpatients on warning signs.

view only

FORM 1: FACILITY AUDIT

FACILITY VISIT ID: [ ][ ][ ] – [ ][ ][ ]

Facility ID e.g. UWK, KAB etc.

Visit ID e.g. 001, 002 etc.

Today's Date: d d – m m – y y y y

Time: [ ][ ] : [ ][ ]

Date previous form completed (or if first Visit, date study opened at this site): d d – m m – y y y y

Are you collecting the data during the baseline or intervention phase? **Baseline** ☐ Intervention ☐

3. How many of the following MATERNAL OUTCOMES have you had since the last visit?

Maternal Outcome	Number
Maternal Sepsis	
Maternal Deaths	
Post-Partum haemorrhage (>1L)	
Ante-Partum haemorrhage (>50ml)	
Severe pre-eclampsia/eclampsia (>160/110 and >2+ protein in urine)	
Blood transfusions	

4. How many of the following NEONATAL OUTCOMES have you had since the last visit?

Neonatal Outcome	Number
Live Births	
Neonatal deaths before discharge from hospital	
Babies requiring antibiotics	
Still births	

For peer review only

FORM 1: FACILITY AUDIT

FACILITY VISIT ID: ☐☐☐ – ☐☐☐

Facility ID e.g. UWK, KAB etc. *Visit ID e.g. 001, 002 etc.*

5. How many of the following RESOURCES are available today?

Resource	Availability		
	**Good ***	**Limited**	**None**
Types of IV fluid:			
A) 0.9% Saline	☐	☐	☐
B) Ringers Lactate	☐	☐	☐
Type of oral antibiotics:			
A) Amoxicillin	☐	☐	☐
B) Augmentin	☐	☐	☐
C) Cephalosporin (e.g. Cefalexin)	☐	☐	☐
D) Cefixime	☐	☐	☐
E) Ciprofloxacin	☐	☐	☐
F) Clindamycin	☐	☐	☐
G) Doxycycline	☐	☐	☐
H) Erythromycin	☐	☐	☐
I) Metronidazole	☐	☐	☐
J) Other (please state):	☐	☐	
Type of IM / IV antibiotics:			
A) Ampicillin	☐	☐	☐
B) Penicillin G	☐	☐	☐
C) Cefazolin	☐	☐	☐
D) Cephalosporin (e.g. ceftriazone etc.)	☐	☐	☐
E) Ciprofloxacin	☐	☐	☐
F) Clindamycin	☐	☐	☐

For peer review only

FORM 1: FACILITY AUDIT

FACILITY VISIT ID: ☐☐☐ – ☐☐☐

Facility ID e.g. UWK, KAB etc. *Visit ID e.g. 001, 002 etc.*

G) Gentamycin	☐	☐	☐
H) Metronidazole	☐	☐	☐
I) Vancomycin	☐	☐	☐
J) Other (please state):	☐	☐	

* Good availability defined as a supply that is unlikely to run out before the next anticipated delivery

**Resource**	**Availability**		
	**Good ***	**Limited**	**None**
Equipment for IV line (cannula, dressings etc)	☐	☐	☐
Malaria tests	☐	☐	☐
Functioning theatre and staff able to remove source of infection	☐	☐	☐
Adequate means to transport to transfer patients for specialist care	☐	☐	☐
Working thermometers	☐	☐	☐
Working BP machines	☐	☐	☐
Working O2 saturation machines	☐	☐	☐
Fetoscopes / Pinnards / fetal stethoscopes	☐	☐	☐
Clocks / watches	☐	☐	☐
Spare batteries	☐	☐	☐
FAST-M Observation charts *1	☐	☐	☐
FAST-M Decision tools *1	☐	☐	☐
FAST-M Treatment tools *1	☐	☐	☐
FAST-M Referral letter *1	☐	☐	☐

* Good availability defined as a supply that is unlikely to run out before the next anticipated delivery

*[1] only applicable if completing form during the intervention phase

For peer review only

FORM 1: FACILITY AUDIT

FACILITY VISIT ID: ☐☐☐ – ☐☐☐

Facility ID e.g. UWK, KAB etc.

Visit ID e.g. 001, 002 etc.

Completed by: ______________________

Role: ______________________

Signature: ______________________ Date: DD / MMM / YYYY

You must have signed the Site Signature & Delegation Log

For peer review only

MODIFIED EARLY OBSTETRIC WARNING CHART (MEOWS CHART)

FAST-M

Contact clinical decision maker if patient triggers ONE RED or TWO YELLOW flags at any one time.

Patient		Patient ID		DOB/Age	

WRITE VALUES IN BOXES PROVIDED

		Date															
		Time															
		Initials															
Respiratory rate (breaths per minute)	25 or more	RED															
	21 - 24	YELLOW															
	11 - 20	NORMAL															
	10 or less	RED															
Oxygen saturations (%)	95 or more	NORMAL															
	94 or less OR needing oxygen	RED															
Temperature (°C)	38 or more	YELLOW															
	36.0 to 37.9	NORMAL															
	35.9 or less	YELLOW															
Heart rate (beats per minute)	120 or more	RED															
	100 - 119	YELLOW															
	50 - 99	NORMAL															
	40 - 49	YELLOW															
	39 or less	RED															
Systolic blood pressure (mmHg)	160 or more	RED															
	140 -159	YELLOW															
	100 -139	NORMAL															
	90 - 99	YELLOW															
	89 or less	RED															
Diastolic blood pressure (mmHg)	110 or more	RED															
	90 -109	YELLOW															
	40 - 89	NORMAL															
	39 or less	RED															
Urine output Hours since patient passed urine (tick box)	12 hours or less	NORMAL															
	12 - 18 hours	YELLOW															
	18 hours or more OR less than 0.5 mL/kg/hour	RED															
Mental State (tick box)	Alert	NORMAL															
	Not Alert	RED															
Looks unwell (tick box)	No	NORMAL															
	Yes	YELLOW															
TOTAL YELLOW FLAGS																	
TOTAL RED FLAGS																	
ACTION TAKEN (IF REQUIRED) Yes (Y) / No (N)																	

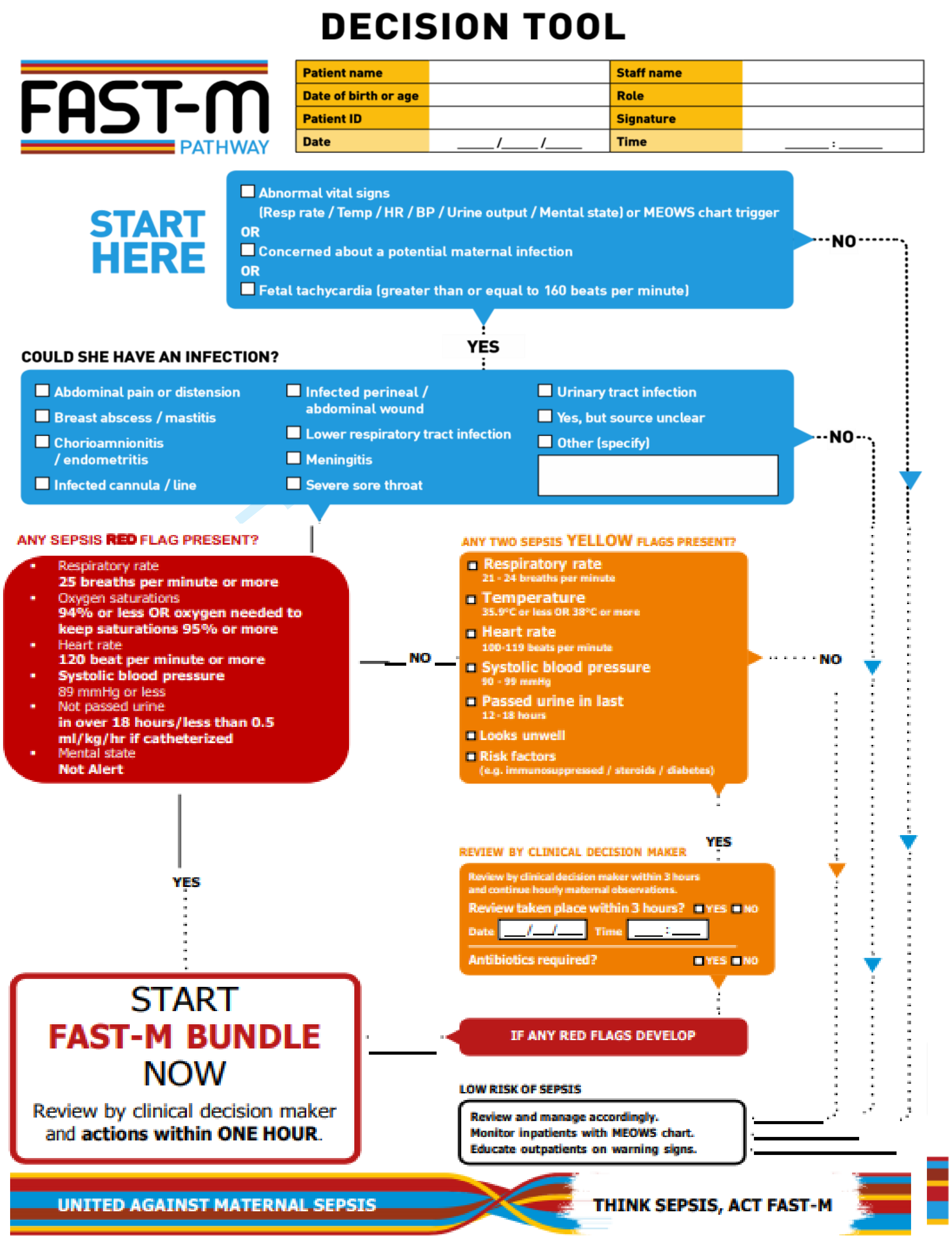
DECISION TOOL
FAST-M PATHWAY
Patient name
Date of birth or age
Patient ID
Date ___/___/___
Staff name
Role
Signature
Time ___:___
START HERE
Abnormal vital signs (Resp rate / Temp / HR / BP / Urine output / Mental state) or MEOWS chart trigger
OR
Concerned about a potential maternal infection
OR
Fetal tachycardia (greater than or equal to 160 beats per minute)
NO
YES
COULD SHE HAVE AN INFECTION?
Abdominal pain or distension
Breast abscess / mastitis
Chorioamnionitis / endometritis
Infected cannula / line
Infected perineal / abdominal wound
Lower respiratory tract infection
Meningitis
Severe sore throat
Urinary tract infection
Yes, but source unclear
Other (specify)
NO
ANY SEPSIS RED FLAG PRESENT?
Respiratory rate
25 breaths per minute or more
Oxygen saturations
94% or less OR oxygen needed to keep saturations 95% or more
Heart rate
120 beat per minute or more
Systolic blood pressure
89 mmHg or less
Not passed urine
in over 18 hours/less than 0.5 ml/kg/hr if catheterized
Mental state
Not Alert
NO
ANY TWO SEPSIS YELLOW FLAGS PRESENT?
Respiratory rate
21 - 24 breaths per minute
Temperature
35.9°C or less OR 38°C or more
Heart rate
100-119 beats per minute
Systolic blood pressure
90 - 99 mmHg
Passed urine in last
12 - 18 hours
Looks unwell
Risk factors
(e.g. immunosuppressed / steroids / diabetes)
NO
YES
REVIEW BY CLINICAL DECISION MAKER
Review by clinical decision maker within 3 hours and continue hourly maternal observations.
Review taken place within 3 hours? YES NO
Date ___/___/___ Time ___:___
Antibiotics required? YES NO
IF ANY RED FLAGS DEVELOP
YES
START
FAST-M BUNDLE
NOW
Review by clinical decision maker and actions within ONE HOUR.
LOW RISK OF SEPSIS
Review and manage accordingly.
Monitor inpatients with MEOWS chart.
Educate outpatients on warning signs.
UNITED AGAINST MATERNAL SEPSIS
THINK SEPSIS, ACT FAST-M

TREATMENT BUNDLE

FAST-M

REMEMBER TO COMPLETE THESE ACTIONS WITHIN ONE HOUR

1HR

Patient name		Staff name			
D.O.B or age		Role / Cadre			
Patient ID		Signature			
Date & time of red flag observation	__/__/__ __:__	Date & time bundle started	__/__/__ __:__	Date & time of review by clinical decision maker	__/__/__ __:__

F

FLUIDS (caution in pre-eclampsia, severe anaemia and pulmonary oedema)

Date	__/__/__	Time fluids initiated	__:__	Initials		Give 500 ml crystalloid immediately. Repeat 500 ml boluses to a maximum of 30 ml/kg if hypotension persists.
Details / reason not completed						

A

ANTIBIOTICS

Date	__/__/__	Time started	__:__	Initials		See antibiotic guidelines below
Details / reason not completed						

S

SOURCE – identify and treat the source of infection

Date	__/__/__	Time considered	__:__	Initials		See source identification and treatment boxes below
Details / reason not completed						

T

TRANSPORT (to higher level hospital or location within hospital, if required)

Date & time transport considered	__/__/__ __:__	Initials		Transport Required	☐ YES ☐ NO
Date & time transport requested	__/__/__ __:__	Initials		☐ N/A	
Date & time patient left facility	__/__/__ __:__	Initials			
Destination					
Reason for any delay					

M

MONITORING (start MEOWS chart if not already started. Repeat observations every 30 minutes until otherwise decided by clinical decision maker)

Date & time monitoring commenced	__/__/__ __:__		Details / reason not completed
Maternal / fetal monitoring should include	• Respiratory rate<br>• Oxygen Saturations<br>• Temperature<br>• Heart rate<br>• Blood pressure	• Urine output<br>• Mental state<br>• Fetal heart rate	
Neonatal monitoring and review commenced	☐ YES ☐ NO ☐ N/A		

ANTIBIOTIC GUIDELINES

Insert local guidance here

Immediate treatment for Maternal Sepsis:

- Ceftriaxone 2 g IV once daily (if no IV access this can be given as 2 IM injections of 1 g in different sites).
- If possible intra-abdominal source add Metronidazole 500 mg IV three times daily or 400 mg PO three times daily.

If above antibiotic regime is not available then give:

- Tazobactam 4.5 g IV daily two time a day
- Meropenem 1 g IV daily two times a day

IDENTIFY THE SOURCE

Consider

- Clinical history
- Clinical examination
- Blood tests (if available) (FBC, U&Es, LFTs, CRP, clotting)
- Blood cultures
- HIV and Malaria testing
- Urine sample
- Swabs (wound, vagina, throat)
- Sputum sample
- Imaging (abdominal, chest)
- Lumbar puncture
- Other ____________

REMOVE / TREAT THE SOURCE

Consider

- Malaria treatment
- Consider delivery of baby
- Removal of retained products of conception
- Debridement of wound / drainage of collection
- Removal of infected cannula / line
- Hysterectomy
- Targeted antibiotics once source known

UNITED AGAINST MATERNAL SEPSIS

THINK SEPSIS, ACT FAST-M
